# Randomized, crossover clinical trial on the safety, feasibility, and usability of the ABLE exoskeleton: A comparative study with knee-ankle-foot orthoses

**DOI:** 10.1371/journal.pone.0318039

**Published:** 2025-05-27

**Authors:** Antonio Rodríguez-Fernández, Joan Lobo-Prat, Mariona Tolrà-Campanyà, Florentina Pérez-Cañabate, Josep M. Font-Llagunes, Lluis Guirao-Cano

**Affiliations:** 1 Biomechanical Engineering Lab, Department of Mechanical Engineering and Research Center for Biomedical Engineering, Universitat Politècnica de Catalunya, Barcelona, Spain; 2 Institut de Recerca Sant Joan de Déu, Esplugues de Llobregat, Spain; 3 ABLE Human Motion, Barcelona, Spain; 4 Rehabilitation Service, Asepeyo Hospital Barcelona, Barcelona, Spain; 5 Rehabilitation Service, Hospital Universitario Mútua Terrassa, Barcelona, Spain; University rehabilitation institute, SLOVENIA

## Abstract

Wearable exoskeletons are emerging as a new tool for gait training. However, comparisons between exoskeletons and conventional orthoses in terms of safety and feasibility are scarce. This study assessed the safety, feasibility, usability, and learning process of using the ABLE Exoskeleton in people with spinal cord injury (SCI) while comparing it with knee-ankle-foot orthoses (KAFOs). In this randomized, crossover clinical trial, 10 patients with chronic complete SCI (T4-T12) conducted a 10-session training and assessment protocol with each device: KAFOs and the ABLE Exoskeleton. Outcomes on safety (adverse events), and feasibility and usability (level of assistance, donning/doffing, therapy activities) were recorded for both devices. Evaluation sessions included standard clinical tests (Timed Up and Go, 10-Meter Walk Test, and 6-Minute Walk Test) to assess gait performance. The therapy metrics (number of steps, distance, gait speed, and standing and walking time) were recorded at each session for the robotic device. Participants quickly learned how to use the ABLE Exoskeleton, showing improvements in all therapy metrics (p<0.05) and the 6-Minute Walk Test (p<0.05). Participants reported less adverse events with the robotic device than KAFOs (17 and 31, respectively). Total donning and doffing time was 43 s faster with the robotic device using comparable levels of assistance. The time to complete the therapy activities was very similar between devices. Overall, participants needed 1 to 4 training sessions to perform essential therapy activities (sit/stand transitions, walking 10 meters, turning around) with both devices using minimum assistance or less. The results of this study show that it is feasible and safe for people with motor complete paraplegia due to SCI (T4-T12) to use the ABLE Exoskeleton for gait training in a rehabilitation hospital setting. The ABLE Exoskeleton proved to be as practical and easy to use as conventional orthoses, with fewer AEs reported when using the exoskeleton versus the KAFOs.

## Introduction

Spinal cord injury (SCI) is a life-changing condition that results in sensory and motor impairments that are often associated with permanent paralysis of the lower limbs [1]. Therefore, recovering the ability to stand and walk independently has a significant impact on participation in social and professional activities [2,3], along with general health and well-being [4–6]. For many years passive orthoses, such as knee-ankle-foot orthoses (KAFOs) –which are the current standard of care for verticalization and gait ambulation in people with SCI–, have served that purpose. However, using these orthoses can often be challenging and inconvenient for the user [7–10].

Gait training using rehabilitation robotic technology has expanded rapidly in the last years due to its advantages over conventional therapy: it increases the duration and intensity of sessions while performing more accurate and continuous physiological movements, and reduces the physical loads of the therapists [11–14]. Since their first appearance in the clinical setting 25 years ago [15], several rehabilitation robots for gait training have been developed, which can broadly be classified into grounded exoskeletons (i.e., large and heavy ground-mounted devices, frequently accompanied by body-weight support systems and treadmills), grounded end-effectors (i.e., similar to the grounded exoskeletons, but the device is attached to the feet rather than to the entire leg), and wearable exoskeletons (i.e., a wearable structure supporting and assisting walking) [16]. In the last years, robotic gait rehabilitation for people with SCI is evolving principally towards wearable exoskeletons, since they promote more active participation of the user than grounded robots and allow ambulation in the community setting [6].

The safety and feasibility of wearable exoskeletons for gait rehabilitation after SCI have been evaluated in previous studies [3,17–24]. In fact, a number of exoskeletons have already been certified for use in the clinical setting [6] or even at home [25,26]. However, comparisons supporting their superiority over conventional passive orthoses to assist locomotion are still scarce. Only a few studies aimed to compare wearable exoskeletons and passive orthoses; however, their main focus was on the comparison of functional performance, energy consumption, and/or patient satisfaction using each of the two systems [27–33]. Thus, the safety, feasibility, and usability of wearable exoskeletons have never been compared to those of passive orthoses, such as KAFOs. Consequently, it is unknown how far wearable exoskeletons differ from conventional passive orthoses in these regards.

In like manner, despite the technological development and the satisfactory acceptance that wearable gait-assistive exoskeletons are having, intensive and long training is necessary to learn to use these devices independently [34–36]. In addition, the number of sessions and the skill needed to control a wearable exoskeleton differs among users and it depends on different aspects such as level of injury (LOI), body mass index (BMI), age, and lifestyle [37]. Various studies tested the use of wearable exoskeletons in people with SCI to perform different tasks [34–40]. However, all the preceding studies assessed hip-knee-powered exoskeletons, and only one used a knee-powered exoskeleton [24]. The latter examined the use of the ABLE Exoskeleton in people with SCI, both with motor complete and incomplete injuries, and primarily in the acute or subacute phase (i.e., onset of paralysis injury within the last year). As a result, little is known about how difficult it is for people with SCI in the chronic phase to operate and learn to use a knee-powered exoskeleton.

We conducted a randomized, crossover clinical trial (NCT04855916) comparing the use of conventional KAFOs against a robotic knee-powered lower limb exoskeleton (i.e., the ABLE Exoskeleton) for gait training in people with SCI. The primary outcome measure of the clinical trial was the metabolic cost of walking using both devices, which was already assessed in [33]. This study presents the secondary analysis of the trial aimed at assessing the safety, feasibility, and usability of the ABLE Exoskeleton within a rehabilitation clinical setting. The investigation includes a comparative analysis with conventional Knee-Ankle-Foot Orthoses (KAFOs) in those regards, while concurrently aiming to gain insight into the learning process associated with the use of the ABLE Exoskeleton.

## Materials and methods

### Study design

This study was a randomized, single-center, crossover clinical trial that compared walking with a knee-powered bilateral lower limb exoskeleton (i.e., the ABLE Exoskeleton) against walking with KAFOs (i.e., the standard of care for verticalization and ambulation outside the clinical setting in people with SCI). The clinical trial was performed at Asepeyo Sant Cugat Hospital (Barcelona, Spain), a center specialized in SCI, from February to August 2021. The time to complete the study for each patient was approximately 12 weeks. All the participants were randomized to one of the groups: KAFO or ABLE. The principal investigator blindly chose one of the 20,000 lines of a book of random numbers [41]. The 10 first numbers of the chosen line (from left to right) were used to allocate each of the participants to one of the two groups following the order of enrollment: even numbers assigned participants to the ABLE group and odd numbers assigned participants to the KAFO group. Lastly, blinding was not possible because group membership was obvious.

The clinical trial (study code: 2020/157-REH-ASEPEYO) was approved by the responsible ethics committee (CEIm Grupo Hospitalario Quirónsalud-Catalunya) and the national competent authority (Spanish Agency of Medicines and Medical Devices (AEMPS), EUDAMED: CIV-ES-21-01-035724). The study conformed to the principles of the Declaration of Helsinki (revised version 2013), the ISO 14155:2011, and the European Regulation MDR 2017/745 on medical devices. The study protocol was first registered at ClinicalTrials.gov on 25/03/2021 (NCT04855916). Note that due to organizational issues, the investigation team failed to register the study before the first participant enrollment. As a result, five participants had been enrolled before registration. Nonetheless, S1 File shows the Clinical Research Ethics Committee’s resolution of the trial protocol, which was accepted before the study’s start date and has not been modified since then; demonstrating the clinical trial’s prospective nature.

### Participants

**Fig 1:**
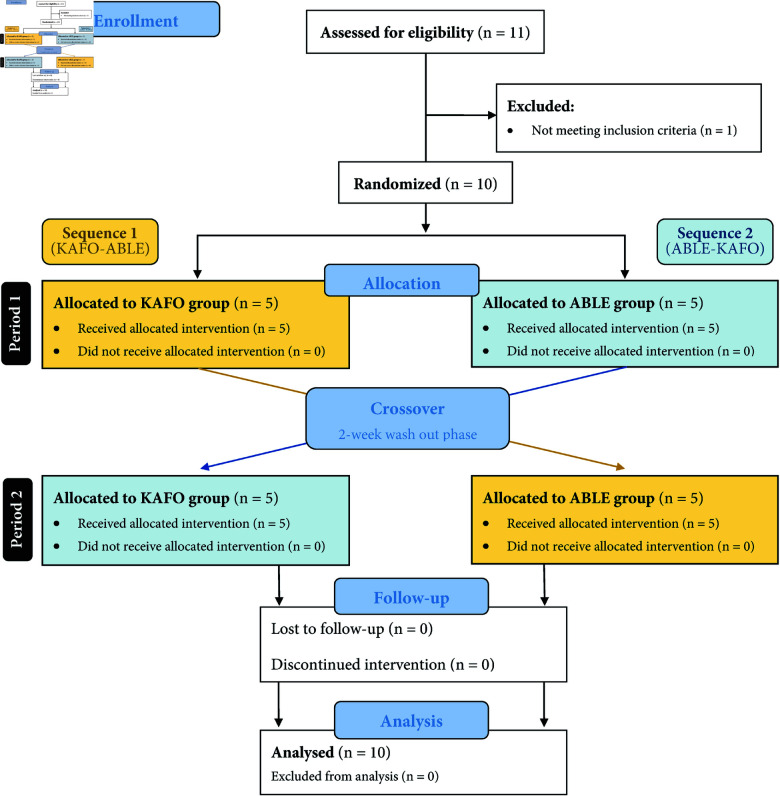
CONSORT flow diagram.

The inclusion/exclusion criteria for this study (Table 1) were designed to recruit patients with SCI at the investigational site. Eleven outpatients with chronic (i.e., time since injury more than one year ago) motor-complete SCI (AIS grade A/B) were assessed for eligibility in this study. One patient was excluded for not meeting the inclusion/exclusion criteria and 10 were enrolled (Fig 1) and completed the entire protocol (Table 2). The neurological LOI of the enrolled participants ranged from T4 to T12. Participants were 44.10 ± 5.93 years old and mostly male (9 out of 10). Seven participants had a traumatic SCI, while three had a non-traumatic SCI. All participants had previous experience using KAFOs, and three of them had previously used other lower-extremity wearable exoskeletons. Prior to data collection, patients provided written informed consent to take part in the study. During the clinical Trial, the medical team, whose members are co-authors of the present publication, had access to the participants’ information.

**Table 1:** Inclusion/exclusion criteria.

Inclusion Criteria	
•	Between 18 and 70 years old
•	Chronic or subacute SCI
•	Currently as an inpatient or outpatient at the participating hospital
•	AIS A to C
•	Previous experience walking with KAFO
•	Capable to give personal consent
**Exclusion Criteria**	
•	CWISCII >16 without exoskeleton
•	C5 or more risk factors of bone fragility [42]
•	Fragility fractures of the lower limbs in the last 2 years
•	Deterioration >3 in the International Standards for Neurological Classification of SCI (ISNCSCI) score in the last 4 weeks
•	Spinal instability
•	Modified Ashworth Scale (MAS) >3 in lower limbs
•	Inability to tolerate 30 minutes of standing without clinical symptoms of orthostatic hypotension
•	Inability to walk 5 meters with KAFO and the help of a walker
•	Psychological or cognitive issues that do not allow a participant to follow study procedures
•	Any neurological condition other than SCI
•	Medically unstable
•	Severe comorbidities, including any condition that a physician deems inappropriate for completing study participation
•	Skin problems
•	Height, width, weight, or other anatomical limitations (such as differences in leg length) incompatible with the device
•	Insufficient joint range of motion (ROM) for the device
•	Known pregnancy or lactation

**Table 2:** Patient demographics.

	Gender	Age	Level of injury	Time since injury	AIS [43]	Height (cm)	Weight (kg)	Sequence
**P1**	M	39	T4	11	A	176	80	KA
**P2**	F	46	T4	7	A	168	70	KA
**P3**	M	44	T4	12	A	170	74	AK
**P4**	M	40	T6	21	A	174	72	AK
**P5**	M	55	T8	5	A	170	80	AK
**P6**	M	47	T11	2	A	183	77	KA
**P7**	M	33	T12	8	A	169	84	KA
**P8**	M	48	T8	10	B	173	75	AK
**P9**	M	46	T11	23	A	185	98	AK
**P10**	M	43	T10	6	B	173	71	KA

AIS: ASIA (American Spinal Injury Association) Impairment Scale; M: Male, F: Female, K: KAFOs, A: ABLE Exoskeleton.

### Study protocol

Participants in both sequences (Fig 1) attended training sessions of 90 minutes duration twice a week for five consecutive weeks in each period, which resulted in a total of 10 sessions with each device: eight overground gait training sessions (sessions 1 to 4 and 6 to 9) and two evaluation sessions (sessions 5 and 10). That is, once all the sessions were completed with one of the devices (Period 1), and after a two-week resting period (Wash-out), participants repeated the same process with the crossed-over device (Period 2). The decision to apply a 2-week washout period was based on similar studies, taking into account the time of device use during the training period [27,31], and the knowledge and experience of the hospital’s clinical staff. Additionally, participants in this study have chronic SCI and are experienced users of KAFOs, regularly utilizing this technology. This familiarity with the KAFOs already mitigates potential carryover effects in this crossover study, as the participants are not starting with two entirely new devices but are already accustomed to using assistive technologies.

The training sessions were carried out by at least one trained therapist plus an additional therapist or assistant if required. During each of the overground gait training sessions, participants spent a minimum of 30 minutes doing sit-to-stand and stand-to-sit transitions, and standing and walking exercises using one of the two devices and the aid of a walker. The training sessions were scheduled for 90 minutes to also include adjustments, donning and doffing time, and data collection time. Moreover, participants performed different therapy activities–grouped by balance abilities, walking abilities, and advanced abilities–that increased in difficulty (Table 3) and were adapted from [36].

**Table 3:** Therapy activities.

Ability	Number	Activity description
Balance	1	Sit-to-stand
	2	Weight shifting
	3	Manipulate remote controller while standing (only for ABLE Exoskeleton)
	4	Touch the head while standing
	5	Stand-to-sit
Walking	6	Walk 10m (with stops)
	7	Start and stop walking with dominant leg
	8	Start and stop walking with non-dominant leg
	9	Walk 10m (without stops)
	10	Turn around
Advanced	11	Walk close to a chair, turn around and sit down
	12	Walk a 90° turn to the right (with stops)
	13	Walk a 90° turn to the right (without stops)
	14	Walk a 90° turn to the left (with stops)
	15	Walk a 90° turn to the left (without stops)
	16	Walk close to an object, stop and manipulate it
	17	Walk in an arrow area
	18	Stop in front of a door, open it (outward), and continue walking
	19	Stop in front of a door, open it (inward), and continue walking
	20	Stop near a wall, turn, and lean against it
	21	Walk on different surfaces (e.g., yoga mat, uneven terrain)
	22	Walk a slalom

In the evaluation sessions, participants performed three standardized clinical tests using each of the two devices (according to the corresponding sequence and period) and the help of a walker: the Timed Up and Go (TUG), the 6-Minute Walk Test (6MWT), and the 10-Meter Walk Test (10MWT). Participants started with the TUG, which consisted of walking back and forth in a three-meter pathway, starting and finishing seated on a chair. Before and after completing the TUG, participants remained in a sitting position for three minutes. This test was repeated twice with a five-minute break in between to recover from fatigue. The 6MWT was conducted after a resting period of 15-20 minutes after the second TUG. Before the 6MWT, participants were asked to rest in a sitting position for three minutes, and, 30 seconds before the end of this resting period, they were asked to stand up and get ready to start. During the test, participants walked in a 10-meter pathway at a comfortable speed selected by themselves. Participants were allowed to stop and rest in a standing position if needed at any time during the test, but the timer continued. The test was completed and finished only when participants reached the 6-minute mark, requested finishing, or sat in a chair due to exhaustion. Finally, the 10MWT was measured during the first 10 meters of the 6MWT.

After the final evaluation session (i.e., session 10), participants answered the Quebec User Evaluation of Satisfaction with Assistive Technology (QUEST 2.0) [44] and the Psychosocial Impact of Assistive Devices Scale (PIADS) [45] questionnaires to evaluate users’ satisfaction and psychosocial impact, respectively, that the training with the corresponding device may have had. Results from the questionnaires were presented previously in [33]. Finally, four weeks after the final evaluation session, a follow-up phone call was conducted to monitor any issues that may have occurred.

The Clinical Investigational Plan (translated to English) can be found in S3 File.

### Gait assistive devices

#### ABLE exoskeleton.

**Fig 2:**
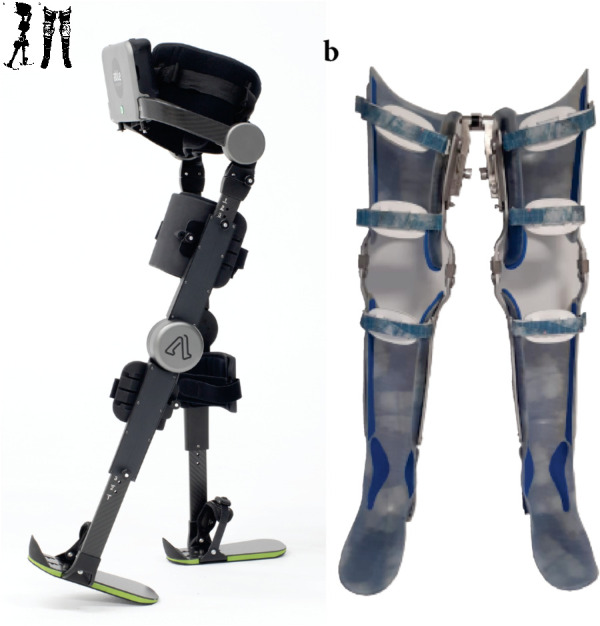
Gait assistive devices. (**a**) Knee-powered lower limb exoskeleton; i.e., the ABLE exoskeleton (ABLE Human Motion S.L., Barcelona, Spain). (**b**) Knee-ankle-foot orthosis with walkabout.

The ABLE Exoskeleton (ABLE Human Motion S.L., Barcelona, Spain) is a knee-powered lower limb exoskeleton that actively assists a person to stand up, walk, and sit down (Fig 2a). It consists of a rigid brace that attaches to the user’s lumbar region, thighs, shanks, and feet via textile straps and rigid supports. The exoskeleton assists with knee flexion-extension movements through battery-powered actuators and allows free hip flexion-extension movements via a passive hinge joint. The exoskeleton size can be adjusted on the length and width of the shank and thigh segments and the hip-width to fit users with a height of 150-190 cm and a maximum weight of 100 kg. The battery is placed on the lumbar module and the total mass of the device is 9.80 kg.

The initiation of each step can be activated manually by the therapist (push-buttons at the lumbar module) or automatically by the patient detecting their intention to step forward, the latter by measuring a change in the thigh angular velocity with an inertial measurement unit (IMU). An automatic step is started by propelling the center of mass forward (i.e., step intention) toward the leading leg. Because there is no hip actuation, the extent of propulsion directly influences the step length. Likewise, the user activates the first step using the same method.

The device comes with a smartphone with the pre-installed software application ABLE Care (ABLE Human Motion S.L., Barcelona, Spain) that allows the therapist to configure the step parameters and monitor the exoskeleton. In addition, the ABLE Exoskeleton presents two operation modes to transition between states (i.e., sitting-to-standing, standing-to-walking, walking-to-standing, and standing-to-sitting) based on the user’s expertise. For beginners, the exoskeleton can be controlled by the therapist using the lumbar buttons or ABLE Care. For more advanced users, state transitions can be controlled by the users themselves through a remote controller attached to the walker.

#### Knee-ankle-foot orthoses.

Knee-ankle-foot orthoses are passive leg braces personalized to each individual that provide stability by locking the knee joint at full extension and the ankle at the neutral position [8]. In general, it consists of an orthopedic thermoplastic cast of the thigh and shank segments attached to metal bars that are connected to a foot insole (Fig 2b).

All the participants in this study used their own KAFOs made of thermoplastic, similar to the ones shown in Fig 2b, that had been prescribed from the investigational site. Note that eight out of the 10 participants used the KAFOs in combination with a multiaxial subperineal hip joint (also known as Walkabout joint [46,47]), that stabilizes stance and provides a reciprocating gait. Additionally, participants wore their own sporty shoes. The average mass of the KAFOs including the Walkabout unit was 3.16 kg (ranging from 2.5 to 3.7 kg).

### Data collection

#### Safety.

The primary endpoints for evaluating the safety of the devices (i.e., the ABLE Exoskeleton and KAFOs) were the number of adverse events (AEs), serious adverse events (SAEs), and study drop-outs related to the use of the device. SAEs and AEs were classified systematically according to ISO 14155:2011, MEDDEV 2.7/3, and MDCG 2020-10/1. AEs were defined in four categories: (1) device-related (AEs that have occurred as a direct result of the device itself), (2) procedure-related (AEs that occurred as a result of the activities performed in training, but were not caused by the device), (3) disease-related (AEs that occurred as a result of the underlying health conditions), and (4) other causes or undetermined relation. Finally, device deficiencies without a medical occurrence were also registered.

Additionally, specific AEs monitoring included: (1) checks for skin lesions pre- and post-session following the European Pressure Ulcers Advisory Panel (EPUAP) scale [48], (2) assessment pre- and post-session of presence, location, and severity of pain using a 0-10 visual analogue scale (VAS), and (3) documentation of falls or any event that required medical intervention. This specific monitoring is also noted in previous studies involving exoskeletons [3,17,18,20–24,34,38]. Furthermore, a (4) medication register was kept, as were any changes to the present medications (at the moment of enrolling in the study).

#### Feasibility and usability.

The primary endpoints for assessing the feasibility and usability of the ABLE Exoskeleton were chosen based on their direct relevance to understanding how well participants can integrate the device into therapy and manage its use independently. Specifically, the primary outcomes were (1) the therapy metrics and (2) the time (number of sessions) and level of assistance (LOA) required for effective device use. These metrics include both the donning/doffing process and the activities performed during training sessions. Previous studies have shown that these factors are essential in determining whether an assistive device is practical and efficient for clinical use [17,20,22,24,32,36].

The secondary endpoints focused on the participants’ gait performance assessed by conducting the following standardized walking assessments: Timed Up and Go (TUG), 6-Minute Walk Test (6MWT), and 10-Meter Walk Test (10MWT); assessed at sessions 5 and 10. The clinical relevance of these metrics is well-supported in the literature, where they are widely used as indicators of gait quality and endurance in individuals with SCI undergoing rehabilitation [3,5,18,20–24,30,38,39]. Additionally, we included step initiation mode as a secondary outcome to explore participants’ adaptation to the device in terms of gait initiation, a critical aspect in using the ABLE Exoskeleton independently.

The donning period began when the participant was ready to transfer from the wheelchair to the stretcher/chair where the device was placed and ended when the device was completely adjusted. The doffing period started when the participant was ready to take off the device until transferred back to the wheelchair. The LOA was recorded in each session for donning/doffing the device, and in sessions 5 and 10 for each of the clinical tests. The LOA was reported by the clinical staff using a rating scale adapted from the Functional Independence Measure (FIM) [49]. The only difference with the FIM was that the modified independence score (i.e., the patient requires the use of a device, but no physical assistance) was removed since all the participants in this study used an assistive device for walking (Table 4). Lastly, note that therapy metrics started to be recorded when the therapist initialized the training session through the mobile app ABLE Care (i.e., generally after donning the exoskeleton) and finished when the therapist ended the session (i.e., generally before doffing the exoskeleton).

**Table 4:** Level of assistance (LOA) definitions for the process of donning/doffing the device and for the therapy activity tasks and standardized clinical tests.

Level of assistance	Donning/doffing	Therapy activity task
Total assistance	Participant performs 0-25% of the effort to don/doff the device. Participant is essentially reliant on the trainer to perform all aspects of the donning/doffing	Participant performs 0-25% of the effort to use the device. Two therapists are required to support the participant in the device at all times
Maximum assistance	Participant performs 25-50% of the effort to don/doff the device. Participant needs maximum assistance to transfer to device and position legs, but may be able to adjust the thigh straps	Participant performs 25-50% of the effort to use the device. Participant needs maximum assistance from the therapist to remain balanced
Moderate assistance	Participant performs 50-75% of the effort to don/doff the device. Participant needs moderate assistance to transfer to device and position legs, but may be able to adjust the thigh and shin straps	Participant performs 50-75% of the effort to use the device. The therapist has both hands on the participant or device at all times to provide occasional guidance or balance support
Minimal assistance	Participant performs >75% of the effort to don/doff the device. Participant can transfer to device and adjust straps, but may need help to position legs	Participant performs >75% of the effort to use the device. The therapist has one hand on the participant or device for infrequent guidance or balance
Supervision	The therapist is not touching the participant, but may provide verbal prompts or contact guarding to ensure safety	The therapist is not touching the participant, but is close enough to provide support for balance or guidance as needed
Independence	Participant is fully independent donning/doffing device	Participant is fully independent while using the device and the therapist does not provide any assistance

The time to complete the TUG was measured from the start of the test until the participant sat down on the chair. For the 6MWT, the distance covered was recorded from the start of the test until the time the test finished or the participant ceased the test and sat down on the chair. The gait speed and cadence during the 10MWT were calculated from the middle-placed six meters of the 10-meter pathway (the first and last two meters were taken for acceleration and deceleration, respectively).

The number of sessions needed to accomplish each of the therapy activities for the first time was recorded by the therapists. The activity was considered as successfully completed if the participant performed the activity with a LOA of *Minimum assistance*, *Supervision*, or *Independence*. The list of the therapy activities was ordered in increasing complexity as shown in Table 3.

#### Comparative analysis and learning process.

For the comparative analysis between devices, we recorded the same primary endpoint metrics mentioned earlier for the KAFOs and compared them to those of the ABLE Exoskeleton. However, certain therapy metrics (i.e., number of steps, distance walked, and standing and walking time) were not recorded for the KAFOs and, therefore, not included in the comparison. Finally, to better understand the learning process of using the ABLE Exoskeleton, we examined, in greater detail, the evolution of the therapy metrics recorded by the device.

### Data and statistical analysis

The sample size of the present clinical study (i.e., 10 patients) was selected taking into account previous clinical investigations that also had the purpose of analyzing the performance of a robotic exoskeleton against passive orthoses (e.g., KAFOs) [27–29,31], and assessing safety, feasibility, and/or usability of wearable exoskeletons for SCI rehabilitation [3,19].

The AEs and SAEs were described using group sizes and frequencies. Quantitative variables were summarized using standard descriptive statistics (i.e., mean, standard deviation (SD), minimum, and maximum).

Statistical analysis of feasibility and usability metrics considered all the study participants. Before we examined the treatment effects between devices, we conducted a preliminary test to check the assumption of negligible carryover effects [50] for all outcome metrics that were feasible for assessment (i.e., quantifiable data in both periods). To this end, the sum of the values measured in the two periods (i.e., period 1 and period 2; see Fig 1) is calculated for each subject and compared across the two sequence groups (i.e., the group that started the study using KAFOs compared to the group that started using the ABLE Exoskeleton) using the Wilcoxon rank-sum test. If this test yields a statistically significant result (i.e., in case of significant carryover effects), only the first period was taken into account for determining the difference between treatments [50]. The treatment effect was tested using the Wilcoxon rank-sum test for the within-subject difference in metric outcome between the two study periods [50]. Note that P10 missed the first training session with the ABLE Exoskeleton and, therefore, we considered the second training session of P10 as if it was the first one for baseline comparisons. Additionally, pairwise comparisons using paired two-tailed t-tests or Wilcoxon signed-rank tests based on the distribution’s normality quantified by the Shapiro-Wilk’s normality test, and supported by visual inspection, were used to assess standardized clinical test improvements between sessions 5 and 10 for each device.

Repeated measures metrics–except for the standardized clinical tests–were analyzed using Generalized Linear Mixed-Effects Model (GLMM) for each device, with training session as the within-subject factor (i.e., fixed effect) and participants as the random effect. Thus, no treatment effects were examined for these metrics. Given the nature of the data—positive values with long tails or variance significantly greater than the mean—GLMM were applied. Gamma distributions were used for continuous data, while Poisson distributions were used for discrete data [51]. When model assumptions were violated, Lognormal and Negative Binomial distributions were applied for continuous and discrete data, respectively [51]. GLMM were built using *lme4* and *glmmTMB* pacakges in R. Model diagnostics for the GLMMs—including normality of random effects, homogeneity of variance, uniformity of residuals, overdispersion, and zero-inflation—were assessed using the *performance* package in R and their functions to check model assumptions and model quality [52]. To identify influential observations, we measured Cook’s distance and DFbetas [53]. We also conducted significance tests to evaluate how the removal of these influential values affected the level of statistical significance. Detailed descriptions of the computational models and their diagnostics can be found in S2 File. Finally, when the models showed a significance for sessions, pairwise comparisons between session 1 and 10 were analyzed using paired two-tailed t-tests or Wilcoxon signed-rank tests based on the distribution’s normality quantified by the Shapiro-Wilk’s normality test, and supported by visual inspection, to assess if participants improved during the training.

For the analysis of metrics assessing the learning process of using the ABLE Exoskeleton evaluation sessions (i.e., sessions 5 and 10) were not included in the statistical analysis due to the differences in the protocol with respect to the gait training sessions. Finally, the LOA and the therapy activities metrics were qualitatively compared.

The level of significance was set to α = 0.05. Statistical analyses were carried out using R version 4.4.0 [54].

## Results

The 10 study participants completed the protocol without deviations, with only P10 missing one training session (session 1 with the ABLE Exoskeleton). The GLMM statistical results are shown in the log scale, as the log link function was used for all the models, except for the metric therapy time where the identity link was used.

### Safety

A total of 48 AEs (KAFO: n = 31, ABLE: n = 17) were registered during the clinical study, from which only six (KAFO: n = 4, ABLE: n = 2) were reported as device-related by the clinical staff. All the AEs were low severity; and no falls, fractures, or device-related SAEs occurred. Other reported events without a medical occurrence were classified as device deficiencies according to ISO 14155:2011. Table 5 shows all the different types of AEs reported during the study classified by device (i.e., KAFO and ABLE) and their cause (i.e., device-related, procedure-related, underlying disease-related, and other causes or undetermined relation).

**Table 5:** Type of adverse events (AEs) that occurred during the clinical investigation.

Type of AE	Device	Number (% out of total AEs)	Device-related^*^	Procedure-related^*^	Underlying disease-related^*^	Other causes or undetermined relation
Skin	KAFO	7 (22.6)	4	2	1	3
	ABLE	7 (41.2)	1	0	0	6
Pain	KAFO	18 (58.1)	0	0	0	18
	ABLE	5 (29.4)	1	1	0	4
Neuropathic pain	KAFO	4 (12.9)	0	0	4	0
	ABLE	3 (17.6)	0	0	3	0
Urinary tract	KAFO	1 (3.2)	0	0	1	0
	ABLE	0	0	0	0	0
Spasticity	KAFO	0	0	0	0	0
	ABLE	2 (11.8)	0	0	2	0
Hypotension	KAFO	1 (3.2)	0	0	1	0
	ABLE	0	0	0	0	0
**Total**	**KAFO**	**31 (100)**	**4**	**2**	**7**	**21**
	**ABLE**	**17 (100)**	**2**	**1**	**5**	**10**

^*^Only AEs rated as “Related” are shown. Otherwise, they are classified as “Other causes or undetermined relation”. The Safety subsection (Results section) contains a detailed explanation of the latter AEs. In addition, note that one AE can be rated in more than one cause-related.

The most reported type of AE for both devices was pain (KAFO = 18; ABLE = 5). Only one of all the AE of pain (n = 23) was considered to be device-related and occurred when using the ABLE Exoskeleton. This AE consisted of pain in the left hand caused by rhizarthrosis in one of the participants (diagnosed before the study), which was intensified by the use of the walker when using the exoskeleton. The issue was resolved by applying a bandage to the affected area of the hand to immobilize the fingers. From the other four AEs of pain reported when walking with the robotic device, two were rated as likely underlying disease-related, which were resolved with resting and thermotherapy, and the other two were low levels of pain in the shoulder that reduced or disappeared during the same training session. Regarding the use of KAFOs, all 18 reported AEs of pain were assigned to other or undetermined causes. Eight of them, classified as likely procedure-related, were from the same participant, the one with rhizharthrosis, who was very skilled at controlling the KAFOs and asked therapists for more intensive training; causing pain in both hands and arms. To mitigate the pain, additional stretching after the training sessions was applied in the affected areas. From the other 10 AEs of pain issues when walking with KAFOs, four were rated as likely underlying disease-related and affecting the lower back; and the rest were one-time pains without major complications rated as unlikely procedure-related and affecting the arms, neck, and back for causes such as participating in sports the day before the session.

In addition to the AEs mentioned above related to pain, there were two participants with neuropathic pain (one in the dorso-lumbar area and one in the supralesional spinal cord) that experienced pain in all the training sessions and with both devices. However, while one of them kept the same level of pain in the affected area across sessions and devices, the other participant kept the pain constant only when using the robotic device and it increased across sessions when using the KAFOs (2-3 values in the VAS between the beginning and end of the session). This last participant also commented that neuropathic pain decreased during the following days after walking with the ABLE Exoskeleton. In addition, there was a third participant with neuropathic pain in the right lower leg who felt foot pain in almost all the sessions during the training with the ABLE Exoskeleton, and only in one session during KAFOs training (foot and ankle pain). All the AEs for neuropathic pain were rated as underlying disease-related.

The total number of AEs due to skin lesions was lower (KAFO = 7; ABLE = 7), though five of them were considered device-related (KAFO = 4; ABLE = 1). One mild pressure injury (grade I according to the EPUAP scale) was reported for the ABLE Exoskeleton and it was due to misuse of the device. The participant wore shorts in one of the training sessions that produced chafing on the skin in contact with the right thigh support. The participant stopped wearing shorts and the skin recovered after two sessions. Device-related skin lesions while using KAFOs were more severe. One of the participants experienced skin irritation in the penis due to friction with the KAFOs during the last evaluation session (i.e., session 10). Moreover, the urine collector intensified the pain and irritation, developing into a little wound. The wound was still under recovery by the end of the study but healing adequately. Another participant suffered grade II skin damage in both ankles during the second training session with KAFOs. For the following sessions, gauze bandages were used to protect the affected zones and the wounds were completely recovered by the follow-up session. Finally, another participant experienced a grade I wound on the right ankle due to chafing with the KAFO (their end device) during session 9. The wound was still recovering by the end of the study.

Other AEs with minor complications and not related to the devices were reported during the study. One of the participants reported having been hospitalized over the weekend due to a urinary tract infection (UTI) during KAFO training. The participant received treatment and recovered without problems. Another participant suffered an isolated episode of orthostatic hypotension at the start of the first evaluation session (i.e., session 5) with the KAFOs, which was classified as likely-related to the underlying disease. Finally, one of the participants experienced irregular spasticity in the abdomen and legs during the first two sessions with the ABLE Exoskeleton. In the first session, clinicians administered a punctual bolus (30 μg in 30 minutes) in an intrathecal baclofen pump. However, during the second session, the patient commented that at home (i.e., outside the sessions) he was also suffering from spasms, and the clinicians decided to increase the basal dose of their baclofen regimen. After that, the appearance of spasticity was reduced and the participant was able to continue the study protocol without further issues. The new basal dose was kept constant until the end of the study.

Lastly, two device deficiencies (KAFO: n = 1, ABLE: n = 1) were reported during the clinical investigation. Regarding the robotic device, a wrong length adjustment of both shank and thigh supports that was considered as “use error” made it impossible for the exoskeleton to fully extend the knees, resulting in an excessive motor torque demand that the exoskeleton could not reach. This made both the patient and the therapist feel the knee joint of the device was not exerting enough force. However, the exoskeleton successfully helped the participant to sit down and, after a proper size configuration of the device, the issue was solved without complications. Concerning the KAFO deficiency, one of the participant’s KAFOs were not rigid enough at the knee joint, producing a flexion of around five degrees while the participant remained upright. The KAFOs were fixed and the participant could continue the study without problems.

### Feasibility and usability

#### Carryover effects.

The first step in our analysis was to look at carryover effects for all of the metrics that were quantitatively evaluated. The analysis found no carryover effects for any of the outcome parameters (Table 6).

**Table 6:** Feasibility and usability metrics.

	KAFO	ABLE	Carryover effect			Treatment effect		
	Median [Q1, Q3]	Median [Q1, Q3]	Estimate [95% CI]	Effect size	p-value	Estimate [95% CI]	Effect size	p-value
**Therapy metrics (Session 9)**								
Therapy time (min)	57.50 [50, 60]	59.00 [54, 70]	-5 [-21, 8]	0.328	0.389	6.73 [-18, 45]	0.328	0.389
Standing time (min)	-	28 [22.50, 43.75]	NA			NA		
Walking time (min)	-	14.50 [9, 19.75]	NA			NA		
Distance (m)	-	105.53 [62.40, 255.15]	NA			NA		
Steps (number)	-	309 [223, 613]	NA			NA		
Average gait speed (m/s)	-	0.14 [0.11, 0.23]	NA			NA		
**Donning/Doffing (Session 10)**								
Donning time (seconds)	284.50 [260, 365]	221.50 [173.75, 337.25]	139 [-153, 509]	0.297	0.421	-73 [-231, 34]	0.561	0.095
Doffing time (seconds)	111.50 [70, 162.50]	129 [79.50, 139.5]	70 [-46, 236]	0.363	0.310	14 [-68, 60]	0.165	0.690
Donning + Doffing (seconds)	392.50 [334.25, 532.50]	358 [230, 498]	216 [-383, 721]	0.245	0.556	-98 [-291, 67]	0.245	0.556
**Clinical outcomes (Session 5)**								
Distance 6MWT (m)	32.10 [17.73, 52.75]	25.85 [16, 42]	0.25 [-95, 123]	0.033	1	-6 [-24, 13]	0.297	0.421
Gait speed 10MWT (m/s)	0.13 [0.06, 0.18]	0.10 [0.05, 0.19]	-0.00 [-0.34, 0.34]	0	1	-0.00 [-0.60, 0.05]	0.033	1
Cadence 10MWT (step/min)	22.22 [16.51, 36.38]	16.42 [12.76, 28.93]	3.86 [-38.5, 54.9]	0.165	0.690	-2.09 [-29.60, 12.90]	0.099	0.841
Time TUG (seconds)	92.75 [68.25, 134.88]	110 [96.50, 157.50]	-42 [-310, 168]	0.033	1	6 [-136, 108]	0.099	0.841
**Clinical outcomes (Session 10)**								
Distance 6MWT (m)	38.50 [29.25, 68.50]	36.6 [24.80, 63]	-5 [-119, 118]	0.099	0.841	6 [-21, 23]	0.033	1
Gait speed 10MWT (m/s)	0.15 [0.09, 0.22]	0.16 [0.09, 0.23]	-0.09 [-0.47, 0.34]	0.495	0.151	-0.04 [-0.12, 0.17]	0.165	0.690
Cadence 10MWT (step/min)	25.21 [18.51, 42.49]	22 [15.93, 37.22]	-13.7 [-53.60, 42.80]	0.231	0.548	-3.66 [-34.20, 23.60]	0.231	0.548
Time TUG (seconds)	80 [60.25, 107.13]	84 [70.13, 137]	41.5 [-140, 150]	0.099	0.841	15 [-130, 144]	0.231	0.548
Therapy Activities^*^								
Sit-to-stand (session)	1 [1, 1]	1 [1, 1]	NA			NA		
Stand-to-sit (session)	1 [1, 1]	1 [1, 1]	NA			NA		
Walking 10 m - without stopping (session)	2.50 [1.25, 3.75]	3 [3, 5.25]	NA			NA		
Turn 180° (session)	2 [1, 2.75]	3 [2, 4]	NA			NA		
Balance abilities (session)	1 [1, 1]	1 [1, 2]	NA			NA		
Walking abilities (session)	2 [1.25, 3]	3 [2, 4]	NA			NA		
Advanced abilities (session)	4 [2.25, 7]	6 [3, 8]	NA			NA		

^*^ Only essential therapy activities (i.e, sit-to-stand transitions, walking 10 meters, and turning around) are shown, along with the values for each ability category (i.e., balance, walking, and advanced; see Table 3).

#### Therapy metrics.

The median therapy time per session for all the participants was 59.00 [54, 70] minutes for the ABLE Exoskeleton and 57.50 [50, 60] minutes for the KAFOs (Table 6). The remaining therapy metrics could only be recorded for the ABLE Exoskeleton and are examined deeper in the *Learning process of using the ABLE Exoskeleton* section. Regarding the KAFOs, participants were able to increase the duration of the training significantly throughout the study (p = 0.009, mean difference = -15.5 minutes, 95% CI = [-26.09, -4.91]).

#### Donning and doffing.

**Fig 3:**
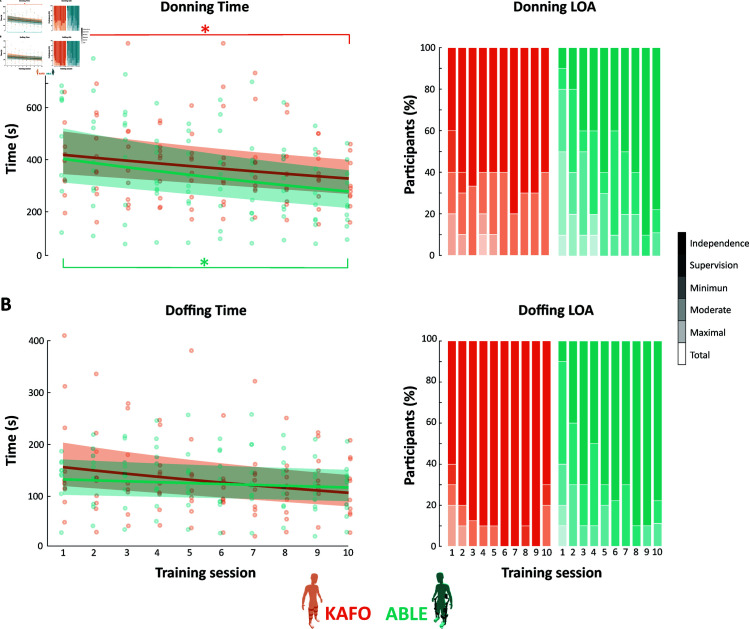
Donning and doffing. (**a**) Time needed and LOA required for donning both the KAFOs and the ABLE exoskeleton for all the sessions of the study. (**b**) Time needed and LOA required for doffing both the KAFOs and the ABLE exoskeleton for all the sessions of the study. Dots indicate the participants’ real time. Solid lines show the general model of the metrics and the faded surfaces indicate the 95% CI of the general models. Stars (*) indicate statistically significant differences (p < 0.05).

Fig 3 shows the average time and the LOA needed to don and doff the devices in each of the study sessions (i.e., gait training sessions plus evaluation sessions).

A GLMM with a lognormal distribution was used to examine the relationship between donning time and training sessions for the two devices (Table 7). For KAFO group, the intercept ($\beta_{0}$) was estimated at 418.59 (95% CI = [344.79, 508.20]) seconds, showing a significant overall effect ($\beta_{0}$ = 6.04, SE = 0.10, z = 61.00, p < 0.000); with a significant negative association between training sessions and donning time ($\beta_{1}$ = -0.03, SE = 0.01, z = -4.07, p < 0.000), meaning donning time decreased by about 2.69% (95% CI = [1,40, 3.96]) for each additional session. Similarly, for ABLE group, the intercept was 402.92 (95% CI = [312.47, 519.56]) seconds, also showing a significant overall effect ($\beta_{0}$ = 6.00, SE = 0.01, z = 46.24, p < 0.000). The session factor ($\beta_{1}$ = -0.04, SE = 0.01, z = -4.50, p < 0.000) indicated a slight, but significant, decrease in donning time (by about 4.05%, 95% CI = [2.30, 5.76]) as the number of sessions increased. Finally, and only for the donning time, we observed a significant reduction from session 1 to 10 for both devices (KAFO: p = 0.017, mean difference = 126.9, 95% CI = [29.05, 224.75]; ABLE: p = 0.027, pseudo-median difference = 242.5, 95% CI = [21 466]).

**Table 7:** Summary of the statistical models.

Model				Model Results						
				Group	Random effects					
Device	Response variable	Regression model	Link function		Std. Dev	Estimate	95% CI	t value	z value	p-value
ABLE	Distance	GLMM	Gamma - Log link*	Intercept	0.63	3.69	[2.93, 4.46]	9.42	-	< 0.000
				Session	0.47	0.15	[0.11, 0.19]	7.76	-	< 0.000
ABLE	Steps	GLMM	Negative binomial - Log link*	Intercept	0.9	5.07	[4.49, 5.65]	17.23	-	< 0.000
				Session	-	0.11	[0.08, 0.15]	6.93	-	< 0.000
ABLE	Walking Time	GLMM	Gamma - Log link*	Intercept	0.36	1.99	[1.51, 2.46]	8.15	-	< 0.000
				Session	0.39	0.09	[0.06, 0.12]	5.38	-	< 0.000
ABLE	Standing time	GLMM	Lognormal - Log link*	Intercept	0.28	3.44	[3.25, 3.63]	-	35.45	< 0.000
				Session	-	0.00	[-0.02, 0.01]	-	-0.13	0.900
ABLE	Session time	LMM	Linear - Identity	Intercept	6.99	52.18	[46.39, 57.96]	17.92	-	< 0.000
				Session	9.38	0.46	[-0.30, 1.23]	1.19	-	0.238
ABLE	Gait speed	GLMM	Lognormal - Log link*	Intercept	0.52	-2.30	[-2.64, -1.96]	-	-13.34	< 0.000
				Session	-	0.04	[0.02, 0.06]	-	4.3	< 0.000
KAFO	Donning time	GLMM	Lognormal - Log link*	Intercept	0.52	6.04	[5.84, 6.23]	-	61.00	< 0.000
				Session	-	-0.03	[-0.04, -0.01]	-	-4.07	< 0.000
ABLE	Donning Time	GLMM	Lognormal - Log link*	Intercept	0.38	6.00	[5.74, 6.25]	-	46.24	< 0.000
				Session	-	-0.04	[-0.06, -0.02]	-	-4.5	< 0.000
KAFO	Doffing time	GLMM	Lognormal - Log link*	Intercept	0.4	5.09	[4.84, 5.35]	-	38.92	< 0.000
				Session	-	-0.04	[-0.06, -0.01]	-	-3.2	0.001
ABLE	Doffing time	GLMM	Lognormal - Log link*	Intercept	0.39	4.89	[4.63, 5.14]	-	37.67	< 0.000
				Session	-	-0.01	[-0.03, -0.00]	-	-2.30	0.022

^*^Log link function provides results on the log scale.

A more detailed table can be found in S2 File.

Similar results were found for the doffing time, where a lognormal distribution was also used for both devices (Table 7). KAFO grouped showed an intercept of 162.81 (95% CI = [125.98, 210.40]) seconds, with a significant overall effect ($\beta_{0}$ = 5.09, SE = 0.13, z = 38.92, p < 0.000); and a significant negative association with session factor ($\beta_{1}$ = -0.04, SE = 0.01, z = -3.20, p = 0.001), meaning doffing time decreased by about 3.62% (95% CI = [1.42, 5.76]) for each additional session. In the ABLE group, the intercept was estimated at 132.66 (95% CI = [102.87, 171.08]) seconds, also showing a significant overall effect ($\beta_{0}$ = 4.89, SE = 0.13, z = 37.67, p < 0.000). The session factor ($\beta_{1}$ = -0.01, SE = 0.01, z = -2.30, p = 0.022) showed a slight and significant reduction (about 1.38%, 95% CI = [0.20, 2.54]) across sessions.

Participants showed improved LOA with the ABLE Exoskeleton troughout the sessions, while maintaining similar levels with KAFOs. Initially, only 50% of the participants could don the exoskeleton with minimal assistance or less, but this improved greatly. By session 5, all participants could don the robotic device with minimal help, and by the end of training, 80% managed to don the device independently. In contrast, 80% of participants could don the KAFOs with minimal assistance from the start, with 60% achieving full independence by the last session and 40% requiring only supervision.

A similar pattern was observed for doffing. Most participants could doff the KAFOs independently or with supervision in nearly all sessions. In the ABLE group, 40% needed minimum assistance or more help during the first session, but by session 6, almost all could doff the exoskeleton with supervision or independently.

#### Step initiation.

Already in session 2, participants walked using the automatic step initiation mode for more than half of the session time (average: 62.40 ± 25.48 % of the session time); and from session 7 to session 10, it was practically for the whole session (more than 90% of the session time, on average). In addition, in the second evaluation session (session 10), nine out of the 10 participants completed the standardized clinical tests (i.e., TUG, 10MWT, and 6MWT) with the automatic step initiation mode and used the remote controller to control state transitions themselves.

#### Therapy activities.

**Fig 4:**
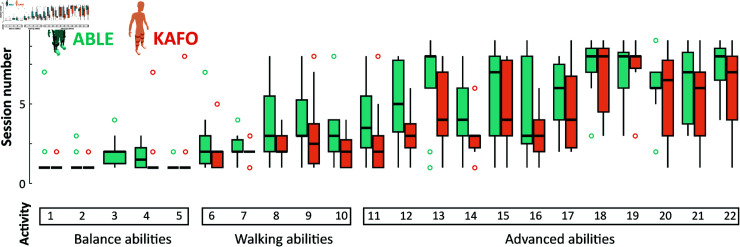
Boxplots showing the number of sessions needed to complete each of the therapy activities with minimum assistance or less.

Table 6 and Fig 4 show the average number of sessions that participants needed to perform each of the therapy activities with minimum assistance or less. When using the ABLE Exoskeleton, all the participants were able to perform *sit-to-stand* and *stand-to-sit* transitions with minimum assistance or less within the first two training sessions; except for one participant, who needed 7 sessions to perform *sit-to-stand* with minimum assistance. Highly similar results were found for the KAFO group, for which all the participants completed both activities within the first two sessions with minimum assistance or less, except for one participant that required 8 training sessions to perform a *stand-to-sit* transition with minimum assistance.

*Walking 10 meters without stopping* with the ABLE Exoskeleton took a median of three sessions, and 70% of the participants did it between sessions 1 and 3. The rest completed the activity in sessions 6 (20%) and 8 (10%). Again, similar results were observed when walking with KAFOs.

Lastly, slightly differences between devices were noticed in the *turning around* activity. When using the ABLE Exoskeleton, all the participants were able to turn 180° between sessions 1 and 4 with minimum assistance or less, except for one participant who completed the activity in session 8. With KAFOs, only one participant required 4 training sessions to complete the activity, while the rest accomplished it within the first three sessions.

Even having previous experience with the KAFOs, the median number of sessions needed to complete all the balance, walking, and advanced therapy activities was very similar between devices, and with no more than 2 sessions of difference (Table 6 and Fig 4).

**Fig 5:**
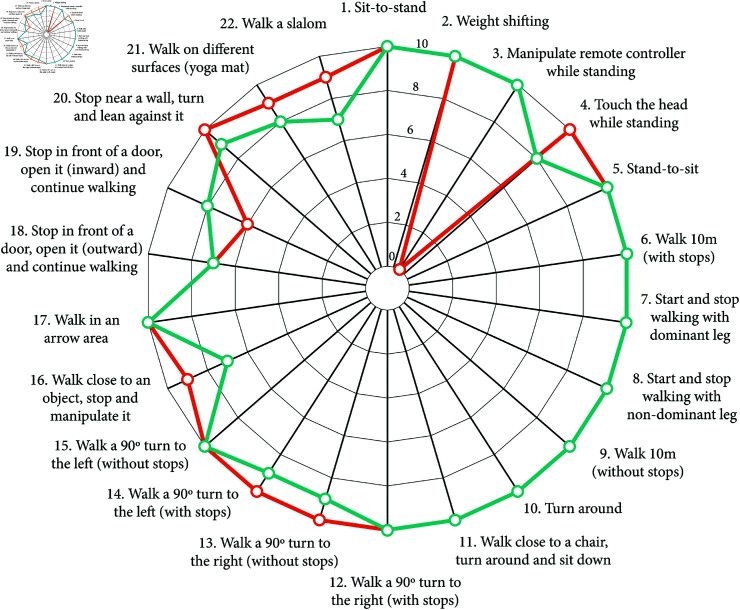
Number of participants that completed each of the ability activities with no more than minimum assistance for both devices.

Finally, note that, despite slight differences, not all the participants completed all the therapy activities with both devices (Fig 5); either because they did not accomplish the activity with minimum assistance or less, or because the therapist considered that the patient did not have the skill to perform the activity yet.

#### Clinical outcomes.

**Fig 6:**
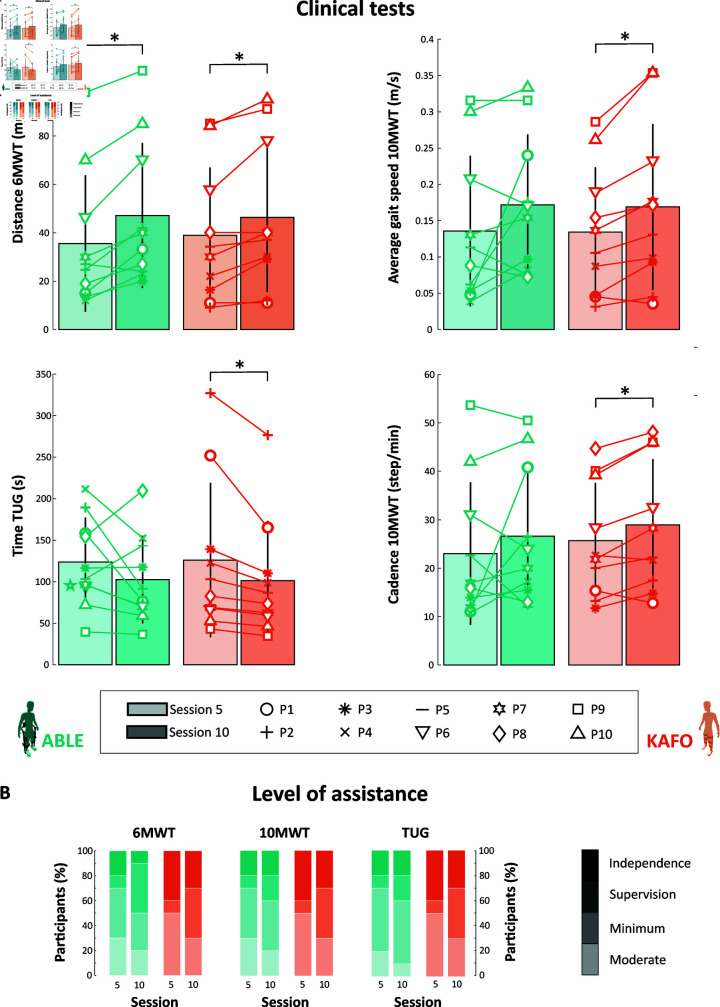
Standardized walking tests. (**a**) Distance covered during the 6MWT, time needed to complete the TUG, gait speed during the 10MWT, and cadence during the 10MWT. Bar plots show the mean and standard deviation from all the participants. (**b**) LOA required to complete the 6MWT, the 10MWT, and the TUG. Stars (*) indicate statistically significant differences (p < 0.05).

Fig 6 shows the results for the three standardized clinical tests. On average, participants improved from the first evaluation session (i.e., session 5) to the final evaluation session (i.e., session 10) in all the tests and for both devices. Nonetheless, significant differences, comparing the first and the final evaluation session, were found only for: distance covered during the 6MWT for both KAFO (p = 0.014, pseudo-median difference = -8.62 meters, 95% CI = [-15.50, -4.40]) and ABLE (p = 0.004, pseudo-median difference = -11.9 meters, 95% CI = [-17, -7.19]); time needed to complete the TUG when using KAFOs (p = 0.006, pseudo-median difference = 17.75 seconds, 95% CI = [7.75, 47.50]); gait speed during the 10MWT when walking with KAFOs (p = 0.009, pseudo-median difference = -0.03 m/s, 95% CI = [-0.05, -0.01]); and cadence during the 10MWT when using KAFOs (p = 0.014, pseudo-median difference = -3.62 step/min, 95% CI = [-5.35, -0.86]).

The LOA required to perform the clinical tests was similar between devices. In session 5, 70% of the participants walked with the robotic exoskeleton with minimum assistance or less during the 10MWT and the 6MWT; and 80% during the TUG. Changes occurred in session 10, where 80% of the participants required minimum assistance or less for the 10MWT and the 6MWT, and 90% for the TUG. The rest of the participants needed moderate assistance to perform the tests. When walking with KAFOs, all the participants were able to complete the three tests with minimum assistance or less in both session 5 and session 10. In session 10, only 30% of the participants walked with minimum assistance, and the others completed the tests with independence (30%) or supervision (40%).

### Learning process of using the ABLE exoskeleton

Note that for this section, the evaluation sessions were not considered in the statistical analysis as they had notable differences with respect to the gait training sessions. Therefore, sessions 1 to 4 and 6 to 9 were considered in this regard.

#### Therapy time.

**Fig 7:**
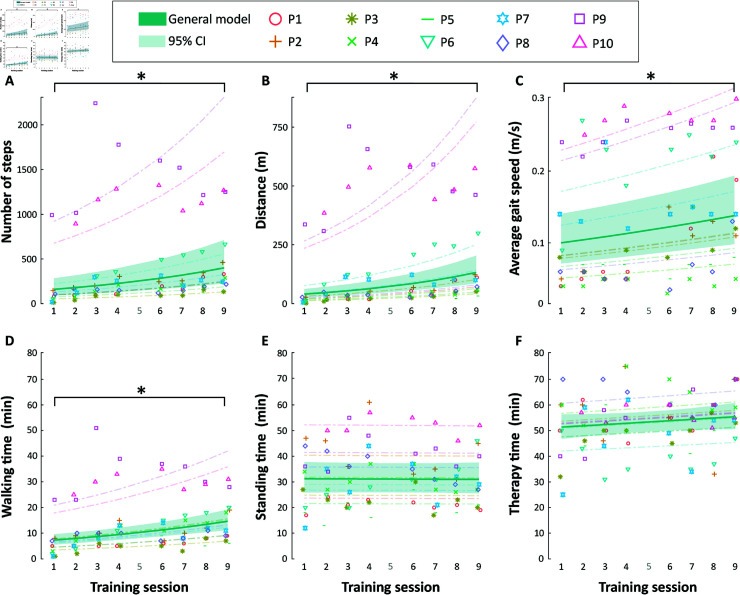
Change of the therapy metrics recorded by the ABLE Exoskeleton during all the training sessions (i.e., sessions 1 to 4 and 6 to 9): (a) number of steps, (b) distance, (c) average gait speed, (d) walking time, (e) standing time, and (f) training session time. Dots indicate the participants’ real time. Solid lines show the general model of the metrics and the faded surfaces indicate the 95% CI of the general models. Dashed lines show the tendency of the individual models for each subject. Stars (*) indicate statistically significant differences (p < 0.05).

The therapy time was almost constant across sessions (Fig 7), keeping an average time per session between 50 and 60 minutes, where 59.26% of this time was spent standing (32:10 ± 11:60 min:s) and 24.07% walking (13:00 ± 10:40 min:s). The standing time also remained constant during the study. However, participants gradually increased their walking time over the training sessions by 9.11% (95% CI = [5.70, 12.64]) per session ($\beta_{1}$ = 0.09, SE = 0.02, t = 5.38, p < 0.000), showing a significant difference between the first and the final training session (p = 0.004, pseudo-median difference = -8 minutes, 95% CI = [-13, -3]). The walking time at session 0 was estimated at 7.29 (95% CI = [4.52, 11.75]) minutes, with significant effect ($\beta_{0}$ = 1.99, SE = 0.25, t = 8.15, p < 0.000).

#### Number of steps.

The number of steps progression was modeled using a negative binomial distribution (Fig 7 and Table 7). The intercept was estimated at 159.30 (95% CI = [89.49, 283.60]) steps, showing a significant effect ($\beta_{0}$ = 5.07, SE = 0.29, t = 17.23, p < 0.000). The session factor also showed a significant effect ($\beta_{1}$ = 0.11, SE = 0.02, t = 6.93, p < 0.000), meaning that participants increased the number of steps by 12.18% (95% CI = [8.59, 15.89]) for each additional session. In fact, participants showed a significant increase between the first and the final training session (p = 0.004, pseudo-median difference = -234.50 steps, 95% CI = [-418.50, -121.00]).

#### Distance.

The distance walked during the training sessions was modeled using a gamma distribution and was highly correlated with the number of steps, showing a similar trend (Fig 7 and Table 7). The intercept was estimated at 40.23 (95% CI = [18.65, 86.79]) meters, showing a significant effect ($\beta_{0}$ = 3.69, SE = 0.39, t = 9.42, p < 0.000). The session factor also showed a significant effect ($\beta_{1}$ = 0.15, SE = 0.02, t = 7.76, p < 0.000), indicating that participants increased the distance walked by 16.04% (95% CI = [11.76, 20.48]) for each additional session. In addition, participants showed a significant increase between the first and the final training session (p = 0.004, pseudo-median difference = -73.39 meters, 95% CI = [-167.61, -46.80]).

#### Average gait speed.

The average gait speed was assessed using a lognormal distribution (Fig 7 and Table 7). The estimated intercept was found to be 0.10 (95% CI = [0.07, 0.14]) m/s, which indicates a statistically significant effect ($\beta_{0}$ = -2.30, SE = 0.17, z = -13.34, p < 0.000). The session variable also demonstrated a significant impact ($\beta_{1}$ = 0.04, SE = 0.01, z = 4.30, p < 0.000), suggesting that for each additional session, participants increased their average gait speed by 4.07% (95% CI = [2.20, 5.98]). Furthermore, a significant improvement was observed between the first and final training sessions (p = 0.014, pseudo-median difference = -0.06 m/s, 95% CI = [-0.11, -0.02]).

## Discussion

This randomized, crossover clinical trial is, to the best of our knowledge the first one that compares the use of a knee-powered lower limb exoskeleton against conventional KAFOs in individuals with motor-complete SCI in terms of safety, feasibility, and usability. Our findings indicated that the ABLE Exoskeleton presents comparable feasibility and ease of use to traditional KAFOs, while showing some advantages in terms of safety.

### Safety

Understanding better the AEs and risks of using wearable exoskeletons through comprehensive reporting is needed to develop safe devices [6,55]. To this end, we performed a detailed and extensive safety assessment of the ABLE Exoskeleton throughout the clinical trial. The same assessment was done for the KAFOs to be able to carry out a comparison in terms of safety between both devices.

In this study, a total of 17 AEs were reported for the ABLE Exoskeleton, while 31 were reported for KAFOs; across 100 gait training sessions each. These AEs were primarily related to skin damage and pain, two well-known risks of using gait assistive devices [6,56].

Skin damage in areas that are in contact with the device is the most common risk of using wearable exoskeletons [6,24,57], even in those with FDA and CE approval: Rewalk [18], Indego [20], and Ekso [3,17]. In this study, only one low-severity AE (out of seven) related to skin damage was reported as device-related when walking with the ABLE Exoskeleton due to skin redness. This result is consistent with the frequency of reported episodes of skin damage in comparable studies, which documented a range of 2 to 27 episodes throughout 6 to 25 sessions, with a sample size ranging from 4 to 32 participants [3,17,18,20,24,57]. Regarding the use of KAFOs, only four AEs (out of seven) of skin type were device-related. Although this result is similar to that of wearable exoskeletons, comparing it to other studies involving KAFOs is difficult because adverse events, such as skin damage, were not systematically reported [58]. However, skin damage appears to be one of the most common reasons for KAFO user dissatisfaction [56], and proper design and collocation to ensure optimal contact between the orthosis and the body is critical [59]. The latter could explain why the skin damage-related AEs reported in this study were more severe when the KAFOs were used, particularly in sensitive areas (e.g., the ankle bones and the groin). In this regard, wearable exoskeletons greatly reduce direct contact with the user’s sensitive areas and may significantly reduce the risk of skin damage when compared to KAFOs.

The use of robotic exoskeletons often leads to pain, particularly in the shoulder, arms, or back, as noted in previous studies [3,6,22,24,34]. This pain is commonly associated with the excessive use of walking aids for balance maintenance during walking [60,61], along with atypical gait patterns [9,33]. Likewise, the observed disparity in the occurrence of pain adverse events between devices (KAFO = 18, ABLE = 5) may be attributed to the more natural gait pattern facilitated by the ABLE Exoskeleton, which allows users to reduce their reliance on walking aids, a topic previously discussed in [33].

Finally, a few underlying-disease-related AEs occurred during the study (e.g., orthostatic hypotension, spasticity, and UTIs), as expected due to the proneness of these issues in people with SCI, which have also been reported in other exoskeleton trials [21,22].

All these findings suggest that the safety of the ABLE Exoskeleton is similar to that of KAFOs and other exoskeletons studied in prior research. Yet, comparing the safety among robotic exoskeletons, as well as exoskeletons versus KAFOs, is challenging since studies tend to omit relevant details and, sometimes, do not explicitly report whether adverse events occurred [55,58].

### Feasibility and usability

Participants, in general, met the recommended standing time of at least 30 minutes per session with the ABLE Exoskeleton, aligning with guidelines for individuals with SCI drawn by Paleg & Livingstone [62]. Walking time gradually increased among sessions, representing approximately half of the standing time by the end of training, with a significant 78.61% increase from the first to the last session. Users typically mastered the automatic step initiation mode within five sessions, with the majority of therapy time spent in this mode from session 7 onwards. These results show the participants’ high adaptability to the device, which is crucial for delivering intensive training from the very beginning of the rehabilitation process.

Throughout the study, participants improved in all gait parameters. Participants increased the average distance covered (74%), the number of steps (71%), and the gait speed (50%) during the eight training sessions. Unfortunately, these metrics cannot be compared with the KAFOs since this type of data was not collected. Likewise, due to the lack of information provided in similar studies and the divergence in the clinical protocols and/or population, direct comparisons with other exoskeletons are difficult to do [3,17,21,22]. Nonetheless, the feasibility and usability of the ABLE Exoskeleton were also assessed in [24]. The latter study reported improvements of 290%, 300%, and 180% in the distance covered, the number of steps, and the gait speed, respectively; which are higher than the ones found in our work. Such variation can be explained by the fact that the LOI (from C5 to L3), the severity of the injury (AIS A to D), and the time since injury (mainly subacute: <1 year after injury) of the studied population in [24] differed from the ones in our study. This hinders the direct comparison of gait parameters’ improvement between studies. Nevertheless, this comparison reveals how population-reliant wearable exoskeletons can be.

Furthermore, participants showed a decrease in the LOA and time needed for both donning and doffing the device. By the end of the training, nearly all participants (8 out of 10) could independently don and doff the ABLE Exoskeleton. The mean average time for donning and doffing in the last session was 06:17 minutes, with doffing being notably easier and quicker than donning. These findings indicate an improvement in donning and doffing times compared to those reported for other commercially available exoskeletons used in clinical settings (i.e., ReWalk, Ekso, and Indego exoskeletons), where previous studies have shown average donning times ranging from 5 to 18 minutes and doffing times ranging from 2:44 to 5 minutes, with assistance levels varying from moderate assistance to independence [17,20,34,39].

Compared to the KAFOs, participants did not show significant differences in the time or LOA required to don/doff the ABLE Exoskeleton. Initially, participants demonstrated better proficiency with donning/doffing KAFOs, mainly due to prior experience. However, this trend changed over the study, with the ABLE Exoskeleton showing faster donning times by the end. This suggests that participants adapted quickly to the ABLE Exoskeleton, as supported by [24], where the average time to don and doff the ABLE Exoskeleton in the last session was 06:50 min:s in a population of predominantly acute and subacute SCI individuals (20 out of 24). The complexity to don and doff robotic exoskeletons is often cited as a barrier to clinical adoption [63]. Nonetheless, our findings suggest that donning/doffing the ABLE Exoskeleton is comparable in time and LOA to KAFOs, which are more widely accepted.

Regarding the therapy activities, the number of sessions required to complete them was similar between devices - despite the prior participants’ experience with KAFOs—with slightly more sessions needed for the exoskeleton. By the end of the training, participants could perform around 22 activities of variant difficulty with minimal assistance with both devices. This suggests that using the ABLE Exoskeleton may be intuitive, as participants learned therapy activities almost as quickly as with KAFOs. These findings contrast with studies on other exoskeletons, where participants required more sessions to achieve similar outcomes. In the study of Kozlowsky et al. [34], participants (mostly in the subacute phase, 43% with motor incomplete SCI) needed a median of 8 sessions to both walk and stand/sit with minimum assistance or less while using a wearable lower limb exoskeleton. In the study of Gagnon et al. [22], where the population examined was more similar to that of this study (mostly chronic with motor complete SCI), 15 sessions (plus two familiarization sessions) were needed to see all the participants walking with minimum assistance or less; and all of them needed moderate to maximal assistance for all sit-to-stand and stand-to-sit transitions throughout the training sessions.

Overall, participants became more skilled over time with both devices, as evidenced by enhancements in the standardized clinical tests (i.e., 10MWT, 6MWT, and TUG). Significant improvements were observed in the distance covered during the 6MWT with the ABLE Exoskeleton. Although significant improvements in the TUG test were not observed, the achieved difference exceeded the minimal clinically important difference [64] (MCID: 10.8 s; ABLE difference: 21.15 s). Possibly, the large variability noted among participants and the limited number of participants reduced the statistical power to get significant differences in the TUG test. Participants using KAFOs also demonstrated significant improvements in the clinical test metrics between evaluation sessions and achieved the MCID for the TUG (KAFO difference: 24.55 s), indicating room for enhancement despite prior experience. This suggests that 8 training sessions may have not been sufficient to fully observe the learning process with the ABLE Exoskeleton, and further improvements could occur with more extensive training. Likewise, these improvements observed with both devices were achieved at the same time that participants reduced the necessary LOA to perform the tests, with most completing them with minimal assistance or less.

In the final evaluation session, participants demonstrated comparable performance metrics between the ABLE Exoskeleton and KAFOs. Yet, these values are still distant from the values reported for other commercially available wearable exoskeletons for overground walking [65,66]. Nonetheless, it is important to interpret the comparison cautiously due to several factors. Firstly, the exoskeletons evaluated in previous studies provided higher levels of walking assistance through hip and knee actuators. Additionally, the characteristics of the study population, such as including individuals with motor incomplete paraplegia, differed from the current study. Furthermore, participants underwent an average of 21 ± 13 training sessions, which may have influenced the outcomes.

In summary, the ABLE Exoskeleton, because is solely actuated at the knee joint, closely resembles the KAFOs. This similarity brings several advantages in some aspects above noted, e.g., the simplicity and speed of donning/doffing the exoskeleton, as well as in the ease of learning to use it. This represents an improvement over exoskeletons actuated at both the knee and hip joints, which tend to be more challenging to control and require a longer learning process with additional training sessions [17,20,22,34,39]. Likewise, compared to KAFOs, the knee actuation of the ABLE Exoskeleton offers improvements in other areas, particularly in improving the gait pattern in terms of kinematics [33]. As previously stated, this more natural gait pattern could have been one of the reasons for the lower number of AEs observed with the ABLE Exoskeleton compared to KAFOs. In contrast, wearable exoskeletons including hip actuation provide other advantages compared to knee actuation only. Given that hip actuation aids in movement facilitation and reduces energy expenditure [27–29,31,67,68], these exoskeletons outperform those with knee-only assistance–and KAFOs as well–in terms of energy efficiency [33], ultimately benefiting therapy metrics and spatiotemporal parameters [33,65,66].

### Learning process

Participants quickly mastered the process of doffing the ABLE Exoskeleton, achieving minimal assistance or less by session 2, with a faster time compared to other exoskeletons (range: 02:44 to 05:00 min:s) [17,20,34,39].

The rapidity with which participants completed therapy activities using the ABLE Exoskeleton also highlights its ease of use. Within just 4 sessions, participants achieved independent control during fundamental tasks like sit-to-stand, stand-to-sit, walking 10 meters, and turning around, often with minimal assistance or less. Moreover, participants quickly progressed to more complex activities. This stands in contrast to findings from Wright et al. (2023) [24], where participants required more sessions and higher levels of assistance for similar activities. Differences in participant populations, particularly between acute/subacute and chronic individuals, may account for these variations. This comparison points out again the relation between exoskeleton performance and population.

In therapy metrics, we observed a slow but progressive improvement throughout the sessions, particularly in steps, distance, gait speed, and walking time. Nonetheless, they did not reach a plateau by the study’s end and, since the time for training with the robotic device was rather short, we speculate that participants mainly remained in the first cognitive stage of motor learning [69], indicating further sessions could enhance performance.

In general, the ABLE Exoskeleton appears to be an easy-to-use wearable exoskeleton, as individuals with complete SCI can achieve a high level of independence with it after about 5 training sessions. Participants quickly adapted to using the ABLE Exoskeleton and demonstrated comparable performance in clinical tests (i.e., 10MWT, 6MWT, and TUG), compared to the KAFOs. Yet, being previous users of KAFOs may have influenced the learning process, potentially benefiting from the skill transfer (i.e., the application of a learned skill in a new task or context) [70] to better use of the ABLE Exoskeleton.

## Limitations and future work

There were some limitations in the present study. The small sample size (10 participants) may have prevented us from achieving enough statistical power. Likewise, while having a diverse range of injury levels among study participants to evaluate the assistive devices is interesting, this diversity may have added more variability to the data, limiting the statistical significance of the results. Furthermore, using the exoskeleton and the KAFOs as intervention devices prevented single or double blinding. Another limitation is that the participants were already used to walking with KAFOs, which may have distorted the comparison. However, comparing subjects who are new to KAFOs and new to the ABLE Exoskeleton is complicated since KAFOs are usually prescribed by the hospital once the patient is discharged at home. Also, the inability to measure therapy metrics such as the number of steps or standing/walking time when using the KAFOs limited the comparison between conventional passive orthoses and robotic gait therapy during training. In like manner, measuring the progress of the LOA needed to complete the therapy activities, instead of only the first session when the activity was completed, could have given a better understanding of the learning process to perform exoskeleton skills. In consonance with that, the method used to evaluate the therapy activities’ progression may have confounded its analysis, as the participants were not required to follow the established order, although the difficulty of the activities gradually increased. Furthermore, we have seen that a larger number of training sessions would be needed to capture the complete learning process of using the ABLE Exoskeleton. Finally, taking into account the low number of sessions that participants needed to learn to control the ABLE Exoskeleton, further studies should consider conducting the baseline session earlier in the training period to obtain more realistic baseline values.

## Conclusion

The results of this clinical study showed that the ABLE Exoskeleton is safe, feasible, and easy to use for gait training in people with motor complete SCI (AIS A and B) with neurological levels ranging from T4 to T12 in a rehabilitation hospital setting. Only 17 low-severe AEs were reported for the robotic device during the study, being only 2 of them device-related. Donning and doffing the device was feasible for all the participants, who needed minimum assistance or less. Overall, participants rapidly improved their skills with the exoskeleton throughout the training, demonstrating improvements in therapy metrics and progressing with the therapy activities. Furthermore, the present study was the first to compare the use of KAFOs (standard of care) against a wearable lower limb exoskeleton (i.e., the ABLE Exoskeleton) for assisting gait in people with SCI in terms of safety, feasibility, and usability. Fewer AEs overall were reported while using the ABLE Exoskeleton compared to while using the KAFOs. Furthermore, the exoskeleton demonstrated to be as practical and easy to use as the conventional passive orthoses. Finally, the insights gained from this clinical trial have been critical in helping the engineers at ABLE Human Motion develop the next version of the ABLE Exoskeleton that includes powered actuation in the hip joints. Clinical trials will be conducted in the future using the new design of the ABLE Exoskeleton.

## Supporting information

S1 File Clinical Research Ethics Committee’s resolution. (PDF)

S2 File Model results. Data sheet with detailed results of the regression model presented in the manuscript.
(XLSX)

S3 File Clinical investigational plan. (PDF)
