# Supplementary material for: Randomized, crossover clinical trial on the safety, feasibility, and usability of the ABLE exoskeleton: A comparative study with knee-ankle-foot orthoses
Source: PLoS One. 2025 May 27;20(5):e0318039. doi: 10.1371/journal.pone.0318039 (PMC12112281; doi:10.1371/journal.pone.0318039)
Supplement: S1 File — (PDF) [file pone.0318039.s001.pdf]

quirónsalud
La salud persona a persona

DICTAMEN DEL COMITÉ ÉTICO DE INVESTIGACION CLÍNICA

MONTSERRAT GRANADOS PLAZA, SECRETARIA DEL **COMITÉ ÉTICO DE INVESTIGACIÓN con Medicamentos (CEIm) GRUPO HOSPITALARIO QUIRÓNSALUD-CATALUNYA,** sito en c/ Pedro i Pons 1, 08195 SANT CUGAT DEL VALLÈS (Barcelona)

C E R T I F I C A:

Que este Comité ha **EVALUADO** en fecha **26/01/2021 (acta nº02/2021)** la propuesta del promotor **ABLE Human Motion, S.L.,** correspondiente al estudio:

Título: ABLE Exoskeleton vs órtesis tipo KAFO: estudio comparativo de la cinemática y la eficiencia energética de la marcha en pacientes con lesión medular.
Código protocolo: ABLEexovsKAFO
Código Interno: 2020/157-REH-ASEPEYO
Protocolo: Versión 4.0, 13/01/2021
Hoja de Información al Paciente/Consentimiento Informado: Versión 3.0 de 22 de enero de 2021

Tomando en consideración que:

- Se cumplen los requisitos necesarios de idoneidad del protocolo en relación con los objetivos del estudio y están justificados los riesgos y molestias previsibles para el sujeto.
- La capacidad del investigador y los medios disponibles son apropiados para llevar a cabo el estudio.
- Son adecuados los procedimientos previstos para obtener el Consentimiento Informado.

Este comité emite **DICTAMEN FAVORABLE** para su realización por el **Dr. Lluis Guirao Cano** como Investigador Principal del servicio de Rehabilitación en **Hospital ASEPEYO.**

Firmado en Sant Cugat del Vallès, a 28 de enero 2021.

GRANADOS PLAZA MONTSERRAT AMELIA - 45474810S

Firmado digitalmente por GRANADOS PLAZA MONTSERRAT AMELIA - 45474810S
Nombre de reconocimiento (DN): c=ES, serialNumber=IDCES-45474810S, givenName=MONTSERRAT AMELIA, sn=GRANADOS PLAZA, cn=GRANADOS PLAZA MONTSERRAT AMELIA - 45474810S
Fecha: 2021.01.28 10:08:29 +01'00'

Montserrat Granados Plaza
Secretaria CEIm Grupo Hospitalario Quirónsalud-Catalunya

Título: ABLE Exoskeleton vs órtesis tipo KAFO: estudio comparativo de la cinemática y la eficiencia energética de la marcha en pacientes con lesión medular.
Código protocolo: ABLEexovsKAFO
Código Interno: 2020/157-REH-ASEPEYO
Investigador Principal: Dr. Lluis Guirao Cano
Servicio: Rehabilitación
Centro: Hospital ASEPEYO

MONTSERRAT GRANADOS PLAZA, SECRETARIA DEL **CEIm GRUPO HOSPITALARIO QUIRÓNSALUD-CATALUNYA** HACE CONSTAR QUE:

1º En la reunión celebrada el día **26 de enero 2021**, se decidió emitir el informe correspondiente al estudio de referencia.
2º En dicha reunión se cumplieron los requisitos establecidos en la legislación vigente para que la decisión del citado CEIm sea válida.
3º El CEIm Grupo Hospitalario Quirónsalud-Catalunya, tanto en composición como en sus **PNTs,** cumple con las normas de **BPC.**
4º Los miembros participantes en la evaluación del estudio son:

Presidente	Dr. Fernando Cereto Castro. Medicina Interna
Vicepresidente	Dr. Rafael Azagra Ledesma. Farmacólogo Clínico
Secretaria	Sra. Montse Granados Plaza. Enfermera
Vocales	Sra. Beatriz Benito Robles. No Sanitario
	Sra. Montserrat Bielsa. Jurista
	Sr. Isidro Díaz de Bustamante Terminel. Jurista
	Sra. Mercedes Gozalbo Mestres. No Sanitario
	Sra. Olga Manrique Rodríguez. Farmacia Atención Primaria
	Sra. M. Mar Martí Ejarque. Enfermera
	Dr. Jordi Peláez de Loño. Farmacia Atención Primaria
	Sr. Iván Ribera Jiménez. No Sanitario
	Sr. Ignacio Rodríguez García. Epidemiología y Estadística
	Dr. Josep Rodiera Anestesiología.
	Sra. Cristina Sagrera Felip. Enfermera
	Sra. Blanca Ricart Cano. Representante Paciente
	Dra. Mª Ángeles Rivas Fernández. Médico Pediatra

En Sant Cugat del Vallés, a 28 de enero de 2020.

GRANADOS PLAZA MONTSERRAT AMELIA - 45474810S

Firmado digitalmente por GRANADOS PLAZA MONTSERRAT AMELIA - 45474810S
Nombre de reconocimiento (DN): c=ES, serialNumber=IDCES-45474810S, givenName=MONTSERRAT AMELIA, sn=GRANADOS PLAZA, cn=GRANADOS PLAZA MONTSERRAT AMELIA - 45474810S
Fecha: 2021.01.28 10:09:06 +01'00'

Montserrat Granados Plaza
Secretaria CEIm Grupo Hospitalario Quirónsalud-Catalunya
