# Supplementary material for: Randomized, crossover clinical trial on the safety, feasibility, and usability of the ABLE exoskeleton: A comparative study with knee-ankle-foot orthoses
Source: PLoS One. 2025 May 27;20(5):e0318039. doi: 10.1371/journal.pone.0318039 (PMC12112281; doi:10.1371/journal.pone.0318039)
Supplement: S3 File — (PDF) [file pone.0318039.s003.pdf]

PLAN DE INVESTIGACIÓN CLÍNICA (PIC)

**Título:** ABLE Exoskeleton vs órtesis tipo KAFO: estudio comparativo de la cinemática y la eficiencia energética de la marcha en pacientes con lesión medular	
**Producto en estudio**	ABLE Exoskeleton
**Promotor**	ABLE Human Motion, S.L.<br>Avda. Diagonal 647, Planta 4 (CREB), Despacho 4.30<br>08028 Barcelona<br>España
**Versión Documento**	4.0
**Investigador/es principal/es**	Dr. Lluis Guirao Cano (Hospital Asepeyo Sant Cugat, Barcelona)
**Centro/s**	Hospital Asepeyo Sant Cugat, Barcelona, España
**Colaboradores académicos del promotor**	Laboratorio de Ingeniería Biomecánica (BIOMEC): grupo acreditado del Centro de Investigación en Ingeniería Biomédica (CREB) de la Universitat Politècnica de Catalunya (UPC)<br><br>Institut Nacional d’Educació Física de Catalunya (INEFC)

Contenidos

1. Historial de versiones 6
2. Resumen del estudio 7
3. Abreviaciones 10
4. Motivación 12
4.1 Antecedentes y estado actual 12
5. Información del producto sanitario 14
5.1 Descripción del producto sanitario en investigación y su uso previsto 14
5.2 Detalles del fabricante 15
5.3 Nombre y/o número de modelo, incluyendo versión de software y accesorios que permita la completa identificación del producto 15
5.4 Trazabilidad (durante y después de la investigación clínica) 16
5.4.1 Suministro de los dispositivos en investigación 16
5.4.2 Almacenaje y manipulación del producto sanitario en investigación 16
5.4.3 Procedimientos de devolución del producto sanitario 17
5.4.4 Requerimientos de desecho del producto sanitario 17
5.5 Uso previsto e indicaciones para el uso del producto sanitario en la investigación clínica propuesta 17
5.6 Descripción del producto sanitario en investigación 17
5.6.1 Materiales 18
5.7 Entrenamiento y experiencia necesaria para utilizar el producto sanitario en investigación 19
5.8 Procedimiento médico o quirúrgico específico 20
5.9 Comparadores 20
5.10 Recepción, distribución y uso 22
6. Riesgo y beneficio del producto sanitario en investigación y de la investigación clínica 22
6.1 Beneficios clínicos esperados 22
6.2 Efectos adversos esperados 23
6.3 Riesgos residuales asociados con el producto sanitario en investigación 23
6.4 Riesgos asociados con la participación en la investigación clínica 24
6.5 Posibles interacciones con tratamientos médicos concomitantes 24
6.6 Estrategias de control o mitigación de riesgos 24
6.7 Justificación del balance positivo riesgo/beneficio 24
6.8 Consideraciones especiales respecto a la pandemia de COVID-19 25
7. Objetivos del estudio e hipótesis 26
7.1 Objetivos 26
7.1.1 Objetivo principal 26
7.1.2 Objetivos secundarios 26

7.1.3 Endpoints 26
7.1.4 Hipótesis 26
7.2 Aplicaciones y beneficios previstos del producto sanitario en investigación a verificar 27
8. Diseño del estudio 27
8.1 Diseño / Plan experimental 27
8.2 Productos en investigación y controles 28
8.2.1 Descripción de la exposición al producto sanitario en investigación 28
8.2.2 Justificación de la selección de los controles 28
8.2.3 Lista de otros dispositivos médicos o medicamentos que se utilizarán durante la investigación clínica 28
8.3 Medidas que se llevan a cabo para minimizar el sesgo 29
8.3.1 Aleatorización 29
8.4 Procedimientos del estudio 29
8.4.1 Reclutamiento de pacientes 29
8.4.2 Procedimiento de selección 30
8.4.3 Procedimiento preliminar / Sesión 0 30
8.4.4 Procedimientos de entrenamiento 31
8.4.4.1 Primera sesión de entrenamiento 33
8.4.4.2 Sesiones 5 y 10 33
8.4.4.3 Sesiones perdidas 34
8.4.5 Procedimientos post-entrenamiento 34
8.4.6 Procedimiento de descanso 34
8.4.7 Procedimientos de seguimiento 35
8.4.8 Medidas finales 35
8.4.9 Diagrama de flujo del estudio 35
9. Medidas de resultados 35
9.1 Recogida de datos generales 35
9.2 Medidas de resultados 37
9.2.1 Objetivos principales del estudio 37
9.2.2 Objetivos secundarios del estudio 37
9.3 Calendario para la valoración, el registro y el análisis de las variables 41
10. Población de pacientes 43
10.1 Número de pacientes a reclutar 43
10.2 Duración de la participación individual en el estudio 43
10.3 Criterios de inclusión y exclusión 43
10.3.1 Criterios de inclusión 43
10.3.2 Criterios de exclusión 43

10.4 Criterios para la retirada de sujetos 44
10.4.1 Suspensión del tratamiento 44
10.4.2 Retirada de la investigación clínica 44
10.5 Duración total prevista de la investigación clínica 44
10.6 Duración prevista del reclutamiento de pacientes 44
10.7 Apoyo médico a los participantes de la investigación clínica 44
11. Estadística 45
11.1 Análisis estadístico previsto 45
11.2 Tamaño de muestra 45
11.3 Gestión de datos ausentes, inutilizados o falsos 46
11.4 Tasas de abandono previstas 46
12. Gestión de eventos adversos 46
12.1 Definiciones 46
12.2 Descripción de posibles eventos adversos y efectos adversos del producto en investigación 48
12.3 Detección y registro 48
12.4 Eventos adversos que requieren notificación 48
12.5 Características de eventos adversos 49
12.6 Notificación del promotor a las autoridades nacionales competentes (ANCs) 50
13. Monitorización 51
13.1 Acceso directo a datos / documentos fuente 52
13.2 Documentos Fuente y Datos Fuente (De acuerdo con la norma ISO 14155:2011) 52
14. Colaboradores académicos del promotor 52
15. Gestión de datos 53
15.1 Procedimientos para la recopilación de datos 53
15.2 Datos fuente 53
15.3 Cuadernos de Recogida de Datos (CRDs) 57
15.4 Revisión y procesamiento de datos 57
15.5 Periodo de retención previsto 58
15.6 Derechos de los pacientes en materia de protección de datos 58
16. Documentación y administración 59
16.1 Manual del investigador (MI) 59
16.2 Informe final 59
16.3 Desviaciones del PIC 59
16.4 Enmiendas al PIC 59
17. Suspensión, Interrupción y Finalización de la Investigación Clínica 60
17.1 Suspensión, interrupción o finalización de un centro de investigación 60
17.2 Suspensión o interrupción anticipada de la Investigación Clínica 60

17.3 Requisitos para el seguimiento de los pacientes 60
18. Consideraciones éticas 60
18.1 Comité de Ética independiente 60
18.2 Cumplimiento de normativa 61
18.3 Hoja de Información al Paciente y el Consentimiento Informado (HIP/CI) 61
18.4 Póliza de seguro 61
18.5 Confidencialidad 62
19. Divulgación de datos y política de publicación 62
20. Referencias 63

1. Historial de versiones

Fecha	Referencia / Número de edición	Contenidos
2020/11/12	v1.0	Primera edición del Plan de Investigación Clínica.
2020/12/02	v2.0	Modificaciones solicitadas por el Comité de ética de investigación clínica (CEIm Grupo Hospitalario Quirónsalud-Catalunya).
2020/12/18	v3.0	Modificaciones solicitadas por la Agencia Española de Medicamentos y Productos Sanitarios (AEMPS).
2021/01/13	v4.0	- Cambio de modelo del ergómetro de brazos utilizado en el estudio.<br>- Procedimiento de Selección: se ha añadido una prueba de levantarse con el exoesqueleto.<br>- Exposición al producto sanitario: Se ha añadido el tiempo de exposición de la prueba de levantarse con el exoesqueleto

2. Resumen del estudio

**Promotor**	ABLE Human Motion, S.L.
**Título del estudio**	ABLE Exoskeleton vs órtesis tipo KAFO: estudio comparativo de la cinemática y la eficiencia energética de la marcha en pacientes con lesión medular
**Código de protocolo**	ABLEexovsKAFO
**Objetivo**	Comparar el estándar de asistencia para verticalizar y caminar en pacientes con lesión medular (órtesis tipo KAFO) con el dispositivo robótico ABLE Exoskeleton mediante el análisis de la cinemática y la eficiencia energética.
**Diseño**	Estudio cruzado aleatorio
**Producto Sanitario**	ABLE Exoskeleton
**Objetivo principal**	Comparar la eficiencia energética en la deambulación con unas órtesis tipo KAFO vs el dispositivo ABLE Exoskeleton en pacientes con lesión medular
**Objetivos secundarios**	▪ Comparar órtesis tipo KAFO vs dispositivo ABLE Exoskeleton:<br>● Análisis cinemático de la marcha:<br>○ Rangos articulares<br>○ Simetría del movimiento<br>○ Normalidad del patrón de marcha<br>● Funcionalidad y usabilidad de los dispositivos:<br>○ Nivel de asistencia y tiempo requeridos para completar las actividades del estudio.<br>● Impacto de la actividad física<br>○ Respuesta cardiorrespiratoria (Ergoespirometría).<br>○ Escalas clínicas<br>● Satisfacción de usuarios (participantes y terapeutas):<br>○ Evaluación de Quebec de la Satisfacción de Usuarios con Tecnología de Asistencia (QUEST 2.0).<br>● Impacto psicosocial en los participantes:<br>○ Escala del Impacto Psicosocial de Productos de Apoyo (PIADS).<br>▪ Evaluar la seguridad de uso del dispositivo ABLE Exoskeleton a través de la evaluación y notificación del número de eventos adversos graves (EAG), eventos adversos (EA) y abandonos causados por el producto.
**Seguimiento clínico**	El estudio incluye las siguientes visitas y seguimiento clínico:<br>● Procedimiento de preselección.

	● Valoración preliminar.<br>● Valoraciones regulares en cada sesión de entrenamiento.<br>● Valoración post-entrenamiento.<br>● Medidas finales<br>○ Al final del ensayo los terapeutas participantes también completarán el QUEST 2.0 para conocer sus puntos de vista sobre el uso del dispositivo en un entorno hospitalario.<br>● Seguimiento
**Número de pacientes**	10 pacientes con una tasa de abandono esperada del 20%.
**Criterios de inclusión**	● 18 a 70 años de edad.<br>● Lesión medular crónica o subaguda.<br>● Actualmente en tratamiento como paciente hospitalizado o ambulatorio en el centro de investigación.<br>● De AIS A a AIS D con suficiente fuerza en los brazos para soportar el peso corporal en un andador.<br>● Experiencia previa caminando con órtesis tipo KAFO (deben tolerar la bipedestación).<br>● Capacidad de dar su consentimiento informado.
**Criterios de exclusión**	● WISCI II >16 sin exoesqueleto.<br>● 5 o más factores de riesgo de fragilidad ósea según lo declarado por Craven et al[1].<br>● Historia de fracturas por fragilidad de los miembros inferiores en los últimos 2 años.<br>● Deterioro > 3 puntos del total en la puntuación motora de las Normas Internacionales para la Clasificación Neurológica de la Lesión Medular (ISNCSCI) en las últimas 4 semanas.<br>● Inestabilidad espinal.<br>● Escala Ashworth modificada (EAM) > 3 en extremidades inferiores.<br>● Incapacidad de tolerar 30 minutos de pie sin síntomas clínicos de hipotensión ortostática.<br>● Incapacidad para caminar 5 metros con órtesis tipo KAFO y la ayuda de un andador con ruedas.<br>● Cuestiones psicológicas o cognitivas que no permitan a un participante seguir los procedimientos del estudio.<br>● Cualquier condición neurológica que no sea LM.<br>● Médicamente inestable.<br>● Comorbilidades severas, incluyendo cualquier condición que un médico considere no apropiada para completar la participación en el estudio.<br>● Problemas en la piel.

	• Altura, anchura, peso u otras limitaciones anatómicas (como diferencias en la longitud de las piernas) incompatibles con el dispositivo.<br>• Rango de movimiento articular (ROM) insuficiente para el dispositivo.<br>• Embarazo o lactancia conocidos.
**Duración estimada**	01.01.2021 - 31.08.2021
**Investigador Principal**	Dr. Lluis Guirao Cano (Hospital Asepeyo Sant Cugat, Barcelona)
**Centros**	Hospital Asepeyo Sant Cugat, Barcelona, España
**Colaboradores académicos del promotor**	Laboratorio de Ingeniería Biomecánica (BIOMEC): grupo acreditado del Centro de Investigación en Ingeniería Biomédica (CREB) de la Universitat Politècnica de Catalunya (UPC)<br><br>Institut Nacional d'Educació Física de Catalunya (INEFC)

3. Abreviaciones

10MWT	Test de marcha de 10 metros
6MWT	Test de marcha de 6 minutos
30MWT	Test de marcha de 30 minutos
AD	Disreflexia autonómica no controlada
AEMPS	Agencia Española del Medicamento y Producto Sanitario
AIS	Escala de discapacidad ASIA
AIT	Análisis por intención de tratar
ANC	Autoridad nacional competente
BPC	Normas de Buena Práctica Clínica
CE	Criterios de exclusión
CEIm	Comité Ético de Investigación con medicamentos
CI	Criterios de inclusión
CO2	Dióxido de carbono
CRD	Cuaderno de Recogida de Datos
DS	Desviación estándar
EA	Evento adverso
EAG	Evento adverso grave
EAGEP	Efecto adverso grave esperado del producto
EAGIP	Efecto adverso grave inesperado del producto
EAGP	Efecto adverso grave del producto
EAM	Escala Ashworth modificada
EAP	Efecto adverso del producto
eCRD	Cuaderno de Recogida de datos electrónico
ECU	Unidad Central Electrónica
EE	Gasto energético

EPUAP	European Pressure Ulcers Advisory Panel
EVA	Escala Visual Analógica
FC	Frecuencia cardíaca
GXT	Prueba de ejercicio máximo gradual
HIP	Hoja de Información al Paciente
IMC	Índice de Masa Corporal
IMU	Sensor Inercial
INEFC	Institut Nacional d'Educació Física de Catalunya
IRGO	Órtesis Recíproca Isocéntrica
ISCoS	Base de datos internacional de lesión medular 2.0
ISNCSCI	Normas Internacionales para la Clasificación Neurológica de la Lesión Medular
KAFO	Knee-Ankle-Foot Orthosis (ortesis de rodilla, tobillo y pie)
LM	Lesión medular
LoA	Nivel de asistencia
MI	Manual del Investigador
NPUAP	National Pressure Ulcer Advisory Panel
O2	Oxígeno
PCI	Índice de coste fisiológico
PIADS	Escala del Impacto Psicosocial de Productos de Apoyo
PIC	Plan de Investigación Clínica
PS	Presión sanguínea
PSD	Presión sanguínea diastólica
PSS	Presión sanguínea sistólica
QUEST 2.0	Evaluación de Quebec de la Satisfacción de Usuarios con Tecnología de Asistencia
RE	Reglamento
RER	Ratio de intercambio respiratorio

RPE	Índice de Esfuerzo Percibido
RGPD	Reglamento General de Protección de Datos
ROM	Rango de movimiento articular
SCIM III	Medida de Independencia en Lesión Medular
TUG	Test "Levántate y anda"
TVP	Trombosis venosa profunda
UPC	Universitat Politècnica de Catalunya
VO2	Consumo de oxígeno
VO2peak	Consumo de oxígeno máximo
WISCI II	Índice de la Marcha en Lesión Medular

4. Motivación

4.1 Antecedentes y estado actual

La lesión medular (LM), es una lesión que cambia la vida de quienes la sufren y que afecta a múltiples sistemas del cuerpo. Los daños en la médula espinal a menudo resultan en impedimentos sensoriales y motores duraderos que pueden causar inmovilidad a largo plazo[2,3,4].

La incidencia de la LM en Europa oscila entre 23.5 nuevos casos por millón cada año en España y 58 por millón en Portugal[5,6]. La recuperación de la marcha se ha identificado como una de las mayores prioridades para los pacientes con LM[2,7], sin embargo, se ha reportado que el nivel de recuperación posible depende del nivel neurológico de la lesión y de si la lesión es completa o incompleta[3,6].

Un número considerable de lesionados medulares mantienen cierto control de la articulación de la cadera (lesiones a nivel torácico o lumbar), pero no de rodilla ni tobillo. Estos pacientes son capaces de caminar utilizando muletas o caminador y unas ortesis pasivas de miembro inferior, también llamadas bitutores o ortesis de rodilla, tobillo y pie (Knee-Ankle-Foot Orthosis, KAFO), que bloquean el giro de rodilla y limitan la flexión plantar del tobillo. Las órtesis tipo KAFO se utilizan con frecuencia en la práctica clínica, siendo el estándar actual para la asistencia de la marcha después de una lesión medular. Estos dispositivos se utilizan durante la rehabilitación con el fin de mejorar la movilidad y obtener beneficios terapéuticos[8,9]. La prescripción de los mismos para la deambulación después de la fase inicial de rehabilitación está más cuestionada[8,9], aunque en países como España siguen siendo la principal solución de asistencia de la marcha que el profesional clínico prescribe al paciente parapléjico cuando abandona el hospital.

El uso de órtesis de miembro inferior para la movilidad funcional es controvertido y difícil de lograr. Es por ello, que muchas personas con LM utilizan este tipo de dispositivos principalmente como elemento deportivo[10–12]. El problema de este tipo de marcha con KAFO es que, al tener siempre la rodilla en extensión total, es necesario levantar anormalmente la cadera para poder dar el paso, lo que supone un coste energético muy elevado[8], ya que se requiere un esfuerzo considerable de las extremidades superiores, tronco y cadera por encima del nivel de la lesión[8,13–17]. Esto lleva a los pacientes a utilizar más tiempo la silla de ruedas. Además, durante la marcha con órtesis los pacientes adoptan estrategias inusuales para lanzar la pierna hacia delante principalmente a

través de movimientos compensatorios de la cadera, como una excesiva oblicuidad pélvica (hip hiking) y abducción de cadera (circumduction)[8,15,17–19]. El excesivo consumo de energía durante la marcha con órtesis ha sido identificado como uno de los problemas principales para su abandono[14,20,21], que es habitual en la mayoría de los pacientes, lo que podría conllevar a una falta de actividad física que predispone a los pacientes a complicaciones de salud.

En los últimos años, la tecnología ha evolucionado como un elemento importante dentro de los programas de entrenamiento de la marcha[22]. Uno de los avances tecnológicos más notables ha sido el desarrollo de exoesqueletos robóticos, con el objetivo de proporcionar a los pacientes la capacidad de realizar múltiples repeticiones de la tarea locomotora con una carga física mínima para los terapeutas[3]. Los exoesqueletos robóticos pueden aumentar la eficiencia energética al caminar comparado con las órtesis mecánicas en personas con LM[23,24]. Además, ofrecen la posibilidad de llevar a cabo actividades de la vida diaria como caminar en la calle y socializar más fácilmente con el entorno[25–27]. En los últimos 10 años, un número reducido de exoesqueletos se ha certificado y fabricado para su uso en hospitales de todo el mundo, mientras que hay muchos otros que se encuentran en la fase inicial de desarrollo o que aún no están certificados para su uso masivo[28]. Existen diferencias sustanciales entre estos exoesqueletos, en cuanto a su peso, tamaño, diseño ortésico y método de control[9]. Actualmente, no hay evidencias claras que surgieran la superioridad de un exoesqueleto sobre los otros. Esto se debe a que se trata de dispositivos nuevos y al hecho de que los ensayos existentes presentan diseños heterogéneos que no permiten una comparación directa de los resultados[28,29,30].

A pesar del rápido crecimiento de los exoesqueletos robóticos en los últimos años y el beneficio que pueden ofrecer frente a las órtesis mecánicas, pocos estudios se han centrado en comparar ambas tecnologías[16,23,31,32]. Uno de los primeros estudios (si no el primero) en comparar la eficiencia energética de la marcha entre órtesis mecánicas y exoesqueletos robóticos en personas con LM se llevó a cabo en 2013 por Arazpour et al.[16]. En este estudio se comparó la marcha con órtesis mecánicas y órtesis motorizadas en 5 personas con LM con una lesión torácica entre T6 y T12 que previamente habían utilizado una órtesis tipo KAFO pero ninguna órtesis motorizada. Los sujetos entrenaron 3 sesiones por semana, de 2 horas cada sesión, durante 8 semanas para cada uno de los dispositivos: (1) KAFO, (2) órtesis recíproca isocéntrica (IRGO) y (3) órtesis motorizada. Al final de cada periodo de entrenamiento, los sujetos realizaban un test para medir el índice de coste fisiológico (PCI) - para el cual es necesario medir la frecuencia cardíaca - seguido de un periodo de descanso de un mes para que el aprendizaje de la órtesis previa no afectara a la siguiente. El test consistió en medir el PCI durante 5 minutos sentado de completo reposo, 5 minutos de pie, test de marcha de los 6 minutos (6MWT) y 2 minutos de recuperación sentado. Los resultados mostraron un aumento en la velocidad de la marcha y la distancia recorrida junto a una reducción del PCI cuando se utilizó una órtesis motorizada en comparación con las órtesis mecánicas.

Farris et al. (2014)[23] compararon el uso de KAFOs frente al exoesqueleto Vanderbilt (actualmente Indego) en una persona con LM completa, tanto sensorial como motora. El sujeto disponía de una experiencia previa de 9 años caminando con órtesis bitutores, normalmente entre 1 y 3 veces por semana para realizar ejercicio. Además, el sujeto había utilizado el exoesqueleto Vanderbilt previamente, aproximadamente 20 veces. En este estudio, además del PCI y el 6MWT, valoraron otras pruebas clínicas como el test de la marcha de 10 metros (10MWT) y el test “levántate y anda” (TUG). Antes de iniciar cada una de las pruebas, el sujeto debía permanecer en reposo hasta que la frecuencia cardíaca estuviera en una tasa de descanso. Las pruebas se realizaron 3 veces cada una y la frecuencia cardíaca se monitorizó durante la realización de las mismas y hasta 30 segundos después de finalizar cada intento, una vez el sujeto se ha sentado. Las 3 pruebas fueron realizadas más rápidamente cuando el sujeto utilizó el exoesqueleto Vanderbilt. Las medidas del esfuerzo indicaron que caminar con el exoesqueleto Vanderbilt requiere entre 1.6 y 3.2 veces menos esfuerzo que caminar con órtesis bitutores.

Un estudio similar, con otro exoesqueleto comercial, fue el que realizaron Yatsuya et al. (2018)[31] con el exoesqueleto WPAL, de sus siglas en inglés *Wearable Power-Assist Locomotor*. En este estudio compararon el exoesqueleto WPAL con un KAFO en 6 personas con una LM completa. Los sujetos tuvieron un entreno previo de entre 1 y 3 meses para cada dispositivo. Igual que en los estudios anteriores se valoró el PCI durante el 6MWT, aunque en este caso también se valoró el esfuerzo percibido (RPE) a través de una medida subjetiva con la calificación de Borg. Además, se realizó un test adicional en el cual los sujetos tenían que caminar continuamente a una velocidad cómoda hasta la fatiga. Como en los casos anteriores, el uso de WPAL llevó a un aumento de la eficiencia energética y, por tanto, a una menor demanda durante la marcha.

Hasta ahora todos los estudios han utilizado medidas indirectas para determinar la demanda energética al caminar (PCI y/o RPE). Kwon et al. (2019)[32], sin embargo, presentó un estudio más completo en el que se utilizó un equipo de medición de gases para medir, entre otras cosas, el consumo de oxígeno (VO2). En este estudio cruzado aleatorio, 10 personas con LM se separaron en 2 grupos y a cada grupo le fue asignado un dispositivo de asistencia a la marcha: órtesis bitutores o exoesqueleto ReWalk. Cada grupo realizó un periodo de entrenamiento de 4 semanas (20 sesiones). Durante el periodo de entrenamiento se realizaron el 6MWT y el test de la marcha de 30 minutos (30MWT) en las sesiones 10 y 20. Una vez finalizado el periodo de entrenamiento hubo un periodo de descanso de 2 semanas, después del cual se intercambiaron los grupos. Para cada test se valoró el PCI, la frecuencia cardíaca máxima, el VO2, el metabolismo equivalente (MET) y el gasto energético (EE). Medidas espacio-temporales de la marcha y datos cinemáticos también fueron estudiados durante el estudio. Además, los sujetos completaron un cuestionario para valorar cada uno de los dispositivos en función de la seguridad, la efectividad, la eficiencia y la satisfacción. Aunque el exoesqueleto ReWalk demostró un consumo más bajo de energía comparado con la órtesis bitutores, el exoesqueleto ReWalk no fue superior en término de satisfacción.

En la marcha natural, la flexión de rodilla permite bajar la cadera durante la fase de balanceo, lo que reduce las oscilaciones del centro de masas, mejorando la eficiencia energética de la marcha. Se espera que el uso de un dispositivo como ABLE Exoskeleton que facilita dicha flexión de rodilla haga que la eficiencia energética de la marcha mejore, promoviendo que los lesionados medulares tiendan más a caminar en lugar de usar la silla de ruedas, con los beneficios que esto implicaría en su rehabilitación y en su salud. La motivación de este estudio es poder comparar la marcha con el estándar de asistencia actual (órtesis pasivas tipo KAFO) con el innovador dispositivo ABLE Exoskeleton.

El objetivo principal de este estudio es determinar la eficiencia energética a través de ergoespirometría y un análisis cinemático de la marcha comparando entre el estándar de asistencia (órtesis tipo KAFO) y el dispositivo ABLE Exoskeleton, un dispositivo robótico para la asistencia a la marcha, en pacientes con LM en un entorno hospitalario. Los objetivos secundarios consisten en evaluar el tiempo y esfuerzo necesarios para aprender a utilizar el dispositivo ABLE Exoskeleton, el impacto que la actividad física tiene en la salud de los pacientes participantes y el grado de satisfacción de los mismos respecto a cada dispositivo.

5. Información del producto sanitario

5.1 Descripción del producto sanitario en investigación y su uso previsto

El dispositivo ABLE Exoskeleton es un exoesqueleto robótico que asiste activamente a levantarse, caminar y sentarse a individuos con problemas de movilidad. Consiste en una órtesis que se acopla al torso, piernas y pies del usuario a través de cintas y soportes rígidos. El exoesqueleto está compuesto por cinco componentes modulares (módulo lumbar, módulos de las piernas derecha e izquierda, y módulos del pie derecho e izquierdo) que se conectan entre sí pesando un total de 9 kg. El módulo lumbar alberga una batería recargable e intercambiable, la Unidad Central Electrónica (ECU, por sus siglas en inglés), un sensor inercial (IMU, por sus siglas en inglés) y módulos Wi-Fi y Bluetooth, mientras que cada módulo de la pierna alberga un actuador

eléctrico (motor más reductor) en la rodilla, un servo-controlador y un sensor inercial (IMU). El módulo del pie consiste en una articulación que bloquea el movimiento del tobillo.

Mientras el usuario está de pie, el actuador aplica el par necesario para mantener sus piernas estiradas. Para detectar la intención del usuario, el ECU en la sección lumbar recibe los datos de movimiento de los sensores del IMU en las secciones de las piernas causadas por los movimientos de la parte superior del cuerpo, analiza los datos e identifica el instante de tiempo en el que se debe iniciar un ciclo de flexión-extensión de la rodilla para lanzar la pierna hacia delante, imitando la trayectoria de una marcha natural. Cada uno de los pasos se desencadena a partir de la detección e interpretación del movimiento mínimo que el paciente aún conserva por encima del nivel neurológico de la lesión. Feedback auditivo y señales visuales de las luces LED en la sección lumbar informan tanto al terapeuta como al usuario del estado del sistema y del estado de operación activo.

El dispositivo ABLE Exoskeleton está diseñado para ser usado con un bastón, muletas o andador para dar soporte a la estabilidad del usuario y siempre bajo la supervisión de un terapeuta entrenado. Si es necesario, el terapeuta puede ayudar al usuario a mantener el equilibrio sujetándolo por la sección lumbar del exoesqueleto, desde donde puede pulsar botones para ejecutar las transiciones entre estados de operación manualmente (levantarse, accionar los pasos manualmente, sentarse).

El exoesqueleto se entrega conjuntamente con un dispositivo Android con una aplicación de software móvil preinstalada (ABLE Care). ABLE Care permite al terapeuta configurar (ajustar el exoesqueleto al usuario correctamente, conocer en todo momento el estado del sistema), operar (ejecutar transiciones entre modos de operación, cambiar en tiempo real los parámetros de la marcha como la flexión de la rodilla o el tiempo de paso) y monitorizar (utilización en tiempo real, seguimiento del progreso del usuario, grabar datos de las sesiones) el exoesqueleto durante la sesión de rehabilitación. ABLE Care se comunica de forma inalámbrica con el exoesqueleto a través de una conexión Bluetooth.

El exoesqueleto incorpora un accesorio para los usuarios avanzados: un mando a distancia que se puede acoplar al bastón, muleta o caminador y permite a los usuarios pasar de un modo de operación a otro de forma autónoma. El mando a distancia se comunica con el exoesqueleto de forma inalámbrica a través de Bluetooth, y proporciona señales visuales y auditivas del estado del sistema. Así, el usuario puede levantarse, caminar y sentarse por sí mismo, siempre con la supervisión de un terapeuta.

En la **Sección 5.6** se incluyen más detalles sobre los elementos funcionales clave del dispositivo.

5.2 Detalles del fabricante

El producto sanitario en investigación es el dispositivo ABLE Exoskeleton fabricado por:

ABLE Human Motion S.L.

Avenida Diagonal 647, 4ª Planta (CREB), Despacho 4.30

08028 Barcelona (España)

Att: Anna Mas Vinyals, PhD (Project Manager)

support@ablehumanmotion.com

5.3 Nombre y/o número de modelo, incluyendo versión de software y accesorios que permita la completa identificación del producto

El ABLE Exoskeleton se entregará conjuntamente con el teléfono móvil con la aplicación ABLE Care instalada, el mando a distancia y las ayudas técnicas para caminar (andador y muletas).

Cada uno de los dispositivos ABLE Exoskeleton en investigación de identificarán con un número de serie en el formato YYXXXX, donde:

- YY es un campo que identifica el modelo de producto. En el caso del producto en investigación, este campo es A1 (ya que ABLE Exoskeleton es el primer producto de ABLE Human Motion S.L.). XXXX es un campo numérico que identifica el número del exoesqueleto. Ejemplo: 0001, 0002, 0003…

Después del número de serie, se añaden dos letras que identifican una parte específica de ese exoesqueleto, siendo:

  - BK: módulo lumbar (“BacK”).
  - RL: pierna derecha (“Right Leg”).
  - LL: pierna izquierda (“Left Leg”).
  - RF: pie derecho (Right Foot”).
  - LF: pie izquierdo (“Left Foot”).
  - RC: mando a distancia (“Remote Controller”).
  - WK: caminador (“WalKer”).
  - CT: muletas (“CruTches”).
  - MP: teléfono móvil (“Mobile Phone”)

Las versiones de software utilizadas para el dispositivo en investigación son las siguientes:

- Aplicación ABLE Care: Versión 1.1.0
- Software integrado en el ABLE Exoskeleton: Versión 1.1.0

Durante el estudio, el promotor puede liberar actualizaciones de menores de software, siempre que no afecten a las funciones principales del producto en investigación ni a los procedimientos clínicos descritos en este documento. Estas modificaciones menores se indican cambiando el último dígito del número de versión.

5.4 Trazabilidad (durante y después de la investigación clínica)

5.4.1 Suministro de los dispositivos en investigación

ABLE Human Motion S.L. facilitará de forma gratuita y debidamente identificado (mediante una identificación o número de serie) el producto sanitario en investigación al centro donde se lleva a cabo la investigación clínica.

5.4.2 Almacenaje y manipulación del producto sanitario en investigación

El producto sanitario en investigación debe ser almacenado en un área segura. El método de almacenamiento debe evitar el uso del producto en investigación para otras aplicaciones que no sean las mencionadas en el presente Plan de Investigación Clínica. El producto sanitario en investigación estará claramente etiquetado como “Exclusivo para investigación clínica”. Toda la información para el uso, almacenamiento y manipulación del producto en investigación se incluye en el manual del investigador y en las instrucciones de uso del dispositivo. Además, para garantizar una mayor seguridad en su manipulación, el producto sanitario en investigación únicamente puede usarse con un teléfono móvil con la aplicación ABLE Care instalada (requiere de un usuario y contraseña individual que se facilita a aquellos profesionales clínicos que han completado con éxito el programa de formación para utilizar el dispositivo).

5.4.3 Procedimientos de devolución del producto sanitario

Los productos no funcionales (dañados) deben ser devueltos a ABLE Human Motion S.L. Antes de devolver el producto, el investigador principal del centro se pondrá en contacto con ABLE Human Motion S.L., quién facilitará las instrucciones para proceder con la devolución.

Al final de la investigación clínica, el producto en investigación debe ser devuelto a ABLE Human Motion S.L. El investigador principal es responsable del mantenimiento de un registro del producto sanitario, donde se documente el recibo, devolución y desecho del producto sanitario en investigación. Al final de la investigación clínica el investigador principal deberá firmar y poner la fecha en el registro.

5.4.4 Requerimientos de desecho del producto sanitario

El promotor mantendrá registros para documentar la ubicación física del dispositivo de investigación desde el envío del mismo al centro de investigación hasta su devolución. Se debe contactar a ABLE Human Motion S.L. en caso de querer desechar un producto sanitario.

5.5 Uso previsto e indicaciones para el uso del producto sanitario en la investigación clínica propuesta

El uso previsto del dispositivo ABLE Exoskeleton es realizar funciones ambulatorias en un centro de rehabilitación bajo la supervisión de un terapeuta entrenado para la siguiente población con lesión medular:

- AIS A a AIS D con suficiente fuerza en los brazos para soportar el peso corporal en un caminador.

El terapeuta debe completar un programa de formación antes de utilizar el dispositivo. El producto sanitario no está diseñado para practicar deporte o subir escaleras.

En las instrucciones de uso y el manual del investigador del producto sanitario en investigación puede encontrarse información detallada sobre el uso previsto del dispositivo, indicaciones y contraindicaciones, así como una lista completa de advertencias, precauciones y eventos adversos posibles.

5.6 Descripción del producto sanitario en investigación

En el Manual del Investigador puede encontrarse una descripción detallada del producto sanitario en investigación y de sus estados de operación. A modo de resumen, el dispositivo ABLE Exoskeleton está compuesto de los siguientes elementos funcionales:

- **Estructura mecánica:** Las partes estructurales del exoesqueleto están compuestas por plástico impreso en 3D, fibra de carbono, y aluminio de grado aeroespacial, para garantizar propiedades mecánicas robustas y un diseño ligero y compacto.

- **Actuador en la rodilla:** se utiliza un actuador eléctrico como músculo artificial para flexionar y extender la pierna. Consiste de un motor eléctrico de alta eficiencia acoplado a un reductor de precisión que incrementa el par motor (40Nm de pico).

- **Sensor inercial (IMU):** La orientación y aceleración de cada pierna se miden con un giróscopo de 3 ejes, un acelerómetro de 3 ejes, y un magnetómetro de 3 ejes.

- **Batería:** Batería recargable de iones de litio certificada (cumple con normativa CE y FCC).

- **Algoritmos de control:** Una máquina de estados identifica, basándose en el movimiento de las piernas, el instante de tiempo en el que el ciclo de flexión-extensión de la rodilla debe ser iniciado para mover la pierna hacia delante durante la marcha. Los algoritmos internos del software mantienen la

pierna estirada cuando el pie está en contacto con el suelo y lanzan una trayectoria en la rodilla que imita el movimiento natural humano cuando se detecta el evento previo.

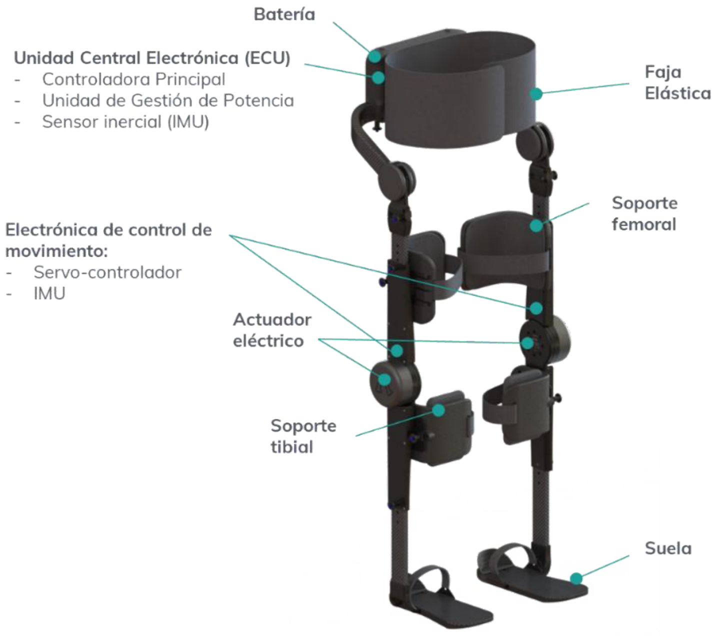

Figura 1: Elementos funcionales clave del dispositivo ABLE Exoskeleton

5.6.1 Materiales

Todos los materiales utilizados en la fabricación del dispositivo ABLE Exoskeleton son biocompatibles con un historial de uso seguro.

En el Manual del Investigador se incluye una descripción detallada de los materiales utilizados para la fabricación de ABLE Exoskeleton y de su biocompatibilidad. También se incluyen los resultados de la evaluación de seguridad biológica de acuerdo con lo establecido en la norma ISO 10993-1:2018.

El producto sanitario debe utilizarse encima de la ropa. Sin embargo, el contacto con la piel de las manos tiene lugar durante su manipulación, ajuste y colocación. Por este motivo, diferenciamos entre los materiales que estarán en contacto con el usuario mientras este utilice el dispositivo, de aquellos que únicamente entran en contacto con las manos durante la manipulación.

La parte estructural del exoesqueleto puede entrar en contacto con las manos durante la manipulación. Para la fabricación de la estructura del dispositivo ABLE Exoskeleton se han utilizado los siguientes materiales (**Figura 2**):

- **Fibra de Carbono (amarillo):** Se utiliza un composite formado por fibra de carbono sarga 2x2 con resina epoxi. Este material tiene la mayor relación rigidez/peso entre todos los materiales disponibles y tiene una alta resistencia.
- **Aluminio (azul):** Este material se ha elegido porque es el material con la mayor relación rigidez/peso entre los metales que se pueden mecanizar. Se han utilizado 4 aleaciones diferentes, dependiendo del rendimiento requerido de las diferentes piezas mecanizadas. En cuanto a la biocompatibilidad, se han analizado en conjunto, ya que todas presentan el mismo tratamiento superficial, y consecuentemente, las mismas propiedades superficiales.

  - Aluminio 7075 T6: Usado en los componentes estructurales críticos, tiene el límite elástico mayor.
  - Aluminio 6061 T6: Usado en los componentes mecanizados no críticos. El coste de esta aleación es más competitivo.
  - Aluminio 5052: Utilizado en componentes no críticos cortados por láser. Esta aleación es perfecta para esta tecnología de fabricación.
- **HP PA 12 (verde):** En este caso, el material se ha elegido debido a la libertad que proporciona la tecnología de impresión 3D, permitiendo obtener piezas con geometrías complejas a un bajo coste.

El exoesqueleto se acopla al torso, piernas y pies del usuario a través de cintas y soportes rígidos. Las cintas de los muslos y pantorrillas están acolchadas y hechas de tejido de nylon común, mientras que para el torso se utiliza una faja comercial elástica y acolchada hecha de neopreno perforado (Bionic Back, Compex). Las partes del producto en contacto con el cuerpo están acolchadas y recubiertas de tejido de nylon común. Los acolchados de la articulación de la cadera y del soporte de la pantorrilla son de la marca Exgel®, mientras que el del muslo lo suministra Handy Free Solutions, ambos proveedores de material ortopédico.

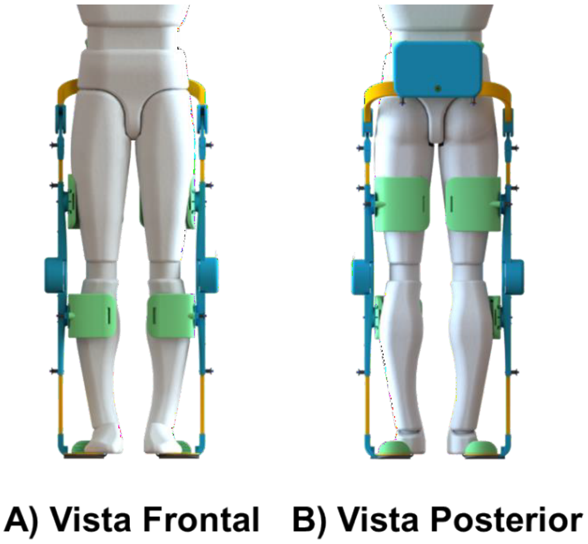

Figura 2: Materiales del dispositivo ABLE Exoskeleton: A) Vista frontal del exoesqueleto; B) Vista posterior del exoesqueleto. Los distintos materiales utilizados se destacan en diferentes colores del modo siguiente: la fibra de carbono aparece de color amarillo, el aluminio de color azul, y el Nylon PA 12 aparece de color verde.

5.7 Entrenamiento y experiencia necesaria para utilizar el producto sanitario en investigación

Antes de la activación del sitio de investigación y la realización de las actividades del estudio clínico, ABLE Human Motion S.L. realizará un programa de formación al personal del centro que llevará a cabo actividades clínicas.

Se facilitará formación específica sobre el uso del dispositivo ABLE Exoskeleton al personal clínico involucrado en su uso durante la validación clínica (ver Sección 3.10 Plan de Formación en el Uso del Producto Sanitario, en el Manual del Investigador (MI)). Además, se suministrarán las instrucciones de uso del producto ABLE Exoskeleton al centro de investigación conjuntamente con el producto sanitario.

Durante la visita de iniciación, el personal será formado en la implementación y documentación de la investigación clínica por el monitor del estudio clínico y el investigador principal.

Las actividades de formación realizadas serán debidamente documentadas.

5.8 Procedimiento médico o quirúrgico específico

No se requiere ningún procedimiento médico o quirúrgico para el uso del exoesqueleto.

5.9 Comparadores

Para esta investigación clínica, el dispositivo en investigación será comparado con el dispositivo de asistencia más utilizado en el tratamiento habitual de la bipedestación y deambulación de pacientes con lesión medular: las órtesis tipo KAFO (**Figura *3***). Cada sujeto utilizará su propio KAFO.

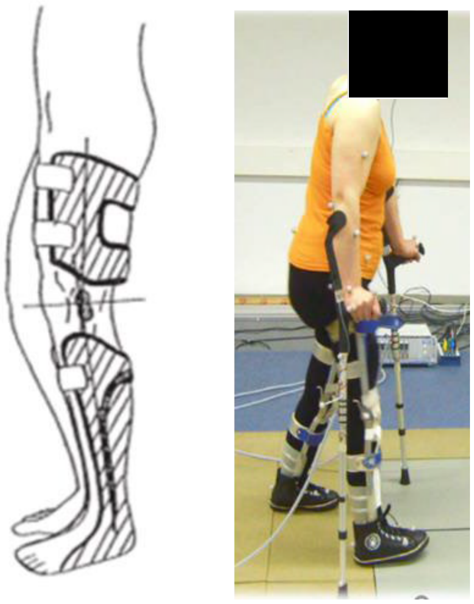

Figura 3: Ejemplo de órtesis tipo KAFO[33] (izquierda) y mujer con lesión medular caminando con muletas y órtesis tipo KAFO[34] (derecha)

Las órtesis tipo KAFO son productos sanitarios a medida y por lo tanto no requieren de marcado CE (Artículo 17(1) Directiva 93/42/CEE del Consejo de 14 de junio de 1993 relativa a los productos sanitarios). Su prestación está regulada por el Decreto 41/2009, de 26 de junio, por el cual se regula la prestación ortoprotésica. El artículo 8 de la Ley 16/2003, de 28 de mayo, de cohesión y calidad del Sistema Nacional de Salud, diferencia dentro de la cartera común de servicios del Sistema Nacional de Salud una cartera común suplementaria en la que se incluye la prestación ortoprotésica realizada mediante dispensación ambulatoria (ortoprótesis externas). El Real Decreto 1030/2006, de 15 de septiembre, recoge en su anexo VI el contenido de la prestación de las ortoprótesis externas (prótesis externas, sillas de ruedas, ortesis y ortoprótesis especiales). Determina los grupos y subgrupos que la integran y en el caso de las ortoprótesis externas, también los códigos homologados que identifican las respectivas categorías de productos. Además, especifica otros aspectos relativos al acceso a la prestación, al procedimiento de obtención y establece los requisitos generales aplicables a dicha prestación.

Una órtesis tipo KAFO es un dispositivo que se incluye en el catálogo de prestaciones ortoprotésicas del Ministerio de Sanidad, Consumo y Bienestar Social[33]: Guía descriptiva de ortoprótesis, Tomo II, Código del subgrupo 06 12 18 Ortesis de cadera, rodilla, tobillo y pie, concretamente el código homologado 06 12 18 004 Órtesis femoral TPV.

Ortesis de miembro inferior 227

Código homologado

06 12 18 004

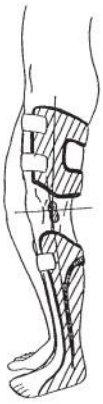

Denominación

Ortesis femoral TPV

Descripción

Ortesis fabricada en polipropileno termoconformado al vacio (TPV) sobre un bitutor de aluminio, con encaje cuadrangular de apoyo isquiático, articulación de rodilla de cierre de anillas o suizo y una férula posterior antiequino dinámica. Cierre anterior.

Mecanismo de acción

La estructura rígida de todos los elementos de la ortesis permite la estabilización-alineación de las articulaciones del miembro inferior para conseguir la bipedestación.

Función

Estabilizar la extremidad inferior, controlar las deformidades y facilitar la marcha.

Indicaciones

Enfermedades que cursan con debilidad muscular de miembros inferiores, como lesiones medulares, mielomeningoceles, secuelas poliomielíticas, etc.
En algunos casos como tratamiento complementario de fracturas, lesiones de partes blandas en las que interesa una descarga para su curación, o tras el tratamiento quirúrgico de lesiones óseas, ligamentosas, etc.

Precauciones de uso

Contraindicaciones

Anestesia de la zona isquiática.

Efectos secundarios

Posibles alergias al plástico.

Recomendaciones de uso

Uso diurno, durante la marcha.

Financiación

Con ayuda económica del Sistema Nacional de Salud.

Vida media

Un año.

Figura 4: Órtesis femoral TPV

Está formada por dos barras verticales (media y lateral) unidas entre sí mediante barras posteriores cuyo objetivo es estabilizar la pierna entera o parte de ella, en función del nivel de lesión. El diseño y material de las órtesis varía en función del fabricante, y se confeccionan a medida para cada usuario.

Estos dispositivos bloquean la rodilla en una posición de máxima extensión (impidiendo tanto la flexión como la hiperextensión) durante el ciclo de la marcha, para prevenir que la pierna colapse durante la fase de apoyo, permitiendo la bipedestación y la marcha. Las barras laterales controlan y estabilizan las desviaciones en el plano frontal de la rodilla. La articulación del tobillo puede ser fija o móvil. En general, estos dispositivos están fabricados de duraluminio, aunque también pueden ser de aluminio, acero inoxidable, termoplástico, titanio y/o fibra de carbono (**Figura *5***).

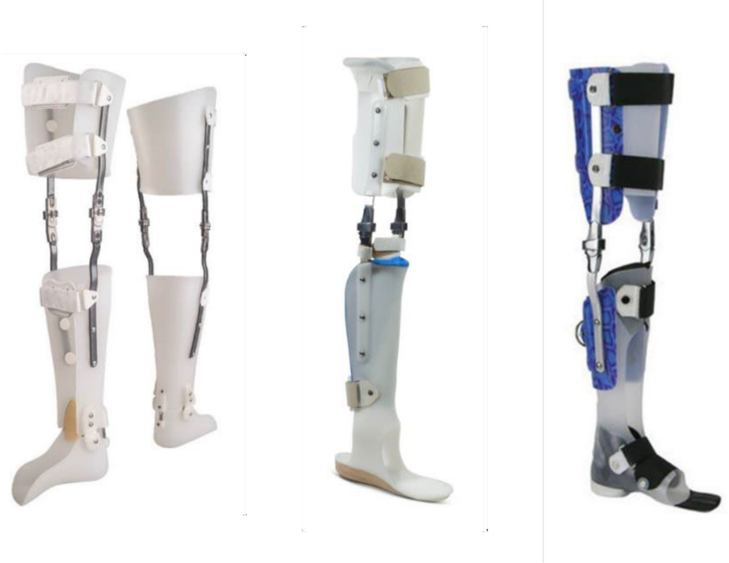

Figura 5: Ejemplos de órtesis tipo KAFO comerciales.

Actualmente los KAFO son el estándar de asistencia y son esencialmente el único dispositivo utilizado durante la fase inicial de rehabilitación después de una LM[9,35]. Además, y aunque de forma más controvertida, los KAFO son altamente prescritos para la movilidad después de la rehabilitación hospitalaria[8,9].

Para más detalles de la presetación de los KAFO, consultar la Orden SCB/45/2019, de 22 de enero, por la que se modifica el anexo VI del Real Decreto 1030/2006, de 15 de septiembre, por el que se establece la cartera de servicios comunes del Sistema Nacional de Salud y el procedimiento para su actualización, se regula el procedimiento de inclusión, alteración y exclusión de la oferta de productos ortoprotésicos.

Actualmente los KAFO son el estándar de asistencia y son esencialmente el único dispositivo utilizado durante la fase inicial de rehabilitación después de una LM[8,9]. Además, y aunque de forma más controvertida, los KAFO son altamente prescritos para la movilidad después de la rehabilitación hospitalaria[8,9].

5.10 Recepción, distribución y uso

La información sobre la recepción, distribución y uso de los productos sanitarios en investigación se describe en la **Sección 5.4** de este documento.

6. Riesgo y beneficio del producto sanitario en investigación y de la investigación clínica

6.1 Beneficios clínicos esperados

Estudios previos han demostrado que el uso de exoesqueletos produce un aumento de la eficiencia energética durante la marcha cuando se comparan con órtesis tipo KAFO en pacientes con LM, así como un aumento en la velocidad de marcha y en la distancia recorrida[16,23,31,32].

Además de la eficiencia energética, se han documentado varios beneficios clínicos relacionados con el uso de exoesqueletos en estudios anteriores con pacientes con lesión medular. La mayoría de los beneficios publicados están relacionados con la mejora funcional de la marcha después del entrenamiento con el exoesqueleto. Varios estudios han reportado una mejora en la marcha al final del entrenamiento en el uso del exoesqueleto[2,3,12,15–22]. Cabe destacar, que dos estudios documentaron mejoras en la marcha al final del entrenamiento sin utilizar el exoesqueleto durante las valoraciones clínicas de la marcha[2,3,36–44]. También se han documentado otros efectos asociados al uso de exoesqueletos como herramientas de rehabilitación, como

mejoras en el estado cardiovascular, la densidad ósea, los movimientos intestinales, el equilibrio y la postura al sentarse, los patrones de sueño y la reducción del dolor y la espasticidad con una menor necesidad de administrar fármacos[2,38,39,44,45].

6.2 Efectos adversos esperados

Varios estudios han afirmado que el uso de exoesqueletos es seguro[15,16,23,46]. Sin embargo, se han notificado eventos adversos (EA) relacionados con el uso de exoesqueletos. Los EA más comúnmente reportados son problemas en la piel, dolor musculoesquelético, caídas, hipotensión ortostática, mareos e hinchazón de las articulaciones[2,3,7,36,37,41,43,47,48,49,50,51]. En algunos estudios se han reportado errores mecánicos como fallos relacionados con el dispositivo[7,36,37,47]. He Y *et al* (2017) describieron el riesgo de errores de usuario que podrían conducir a resultados diferentes de los esperados por el fabricante o usuario[29]. Los mismos autores afirman que podría existir un riesgo de efectos secundarios a largo plazo, pero todavía no se ha realizado ningún estudio al respecto.

Los únicos eventos adversos graves que se han reportado en estudios individuales son fracturas por fragilidad. Un meta-análisis realizado por Miller *et al.* (2016) reportó una incidencia del 3.4% de fracturas óseas relacionadas con la marcha asistida por exoesqueletos[22]. Sin embargo, un informe de Herpern *et al.* (2019) reportó dos incidentes de fracturas óseas a causa del uso de exoesqueletos en personas con LM crónica y osteoporosis. Esto remarca la necesidad de una detección adecuada del perfil de riesgo de fracturas y de una formación suficiente sobre la correcta alineación de las articulaciones técnicas del exoesqueleto con las articulaciones anatómicas del usuario[51]. Un estudio realizado por Gagnon et al. (2018) también reportó un participante que sufrió fracturas bilaterales del calcáneo y abandonó el programa[37]. He Y et al. (2017), remarcan la necesidad de que en los estudios futuros se analicen riesgos como caídas, fracturas, y efectos adversos a largo plazo causados por el uso de exoesqueletos[28].

En cuanto a estudios previos de la literatura que compararon dispositivos robóticos con órtesis tipo KAFO, no se ha documentado ningún efecto adverso[16,23,31,32].

6.3 Riesgos residuales asociados con el producto sanitario en investigación

Se ha llevado a cabo un análisis de riesgo del producto sanitario en investigación de acuerdo con la ISO 14971:2019. Los detalles del análisis de riesgo y sus resultados se incluyen en el Manual del Investigador (Sección 7).

A modo de resumen, todos los riesgos residuales de nivel medio que queden después de la aplicación de las estrategias de control/medición podrían dar lugar a caídas o bien a lesiones de la piel (abrasiones o úlceras por presión):

- Una caída es un riesgo implícito en cualquier dispositivo de exoesqueleto y no puede evitarse con acciones de control razonables. Sin embargo, el riesgo se reduce asegurándose de que el terapeuta supervise constantemente el funcionamiento del exoesqueleto y que el usuario utilice constantemente un dispositivo de ayuda para caminar. Además, existen ciertos riesgos de errores de uso del usuario/terapeuta que sólo pueden abordarse desde el punto de vista de la formación y la orientación. Por lo tanto, es obligatorio un entrenamiento completo para todos los terapeutas que deseen utilizar este dispositivo. En cualquier caso, no podemos asegurar la falta de ocurrencia.

- Las abrasiones de la piel o las úlceras por presión son un evento adverso frecuente del uso de exoesqueletos para la rehabilitación de pacientes con lesión medular. Por lo tanto, consideramos que el riesgo está implícito con el uso del dispositivo. El riesgo se reduce mediante la incorporación de acolchados apropiados en las superficies del exoesqueleto en contacto con el cuerpo (un gel especial en los puntos de contacto más críticos que distribuye mejor la presión aplicada) y mediante la

realización de comprobaciones de la integridad de la piel antes y después de la sesión de entrenamiento con el exoesqueleto (los terapeutas recibirán formación sobre cómo hacerlo durante el programa de formación para la certificación). Si surgiera un problema de la piel, los especialistas clínicos evaluarán las acciones a tomar teniendo en cuenta la severidad de la lesión. Aunque la aparición de problemas cutáneos relacionados con el uso del exoesqueleto no es insignificante, el beneficio potencial que podrían obtener los pacientes supera el riesgo.

6.4 Riesgos asociados con la participación en la investigación clínica

Como se ha destacado en la **Sección 6.2**, los riesgos de la participación en la investigación clínica incluyen:

- Lesiones en la piel en los puntos de contacto con el exoesqueleto.
- Dolor musculoesquelético.
- Caídas.
- Síntomas de hipotensión ortostática.
- Hinchazón en las articulaciones.
- Fracturas.

6.5 Posibles interacciones con tratamientos médicos concomitantes

En el procedimiento de preselección, se recogerá información sobre los medicamentos y tratamientos médicos relevantes para la función de la marcha, tales como espasmolíticos, medicamentos para el dolor, medicamentos cardiovasculares, y sus dosis. Cualquier cambio en la medicación se registrará semanalmente. No se esperan problemas en relación con interacciones con tratamientos médicos concomitantes.

6.6 Estrategias de control o mitigación de riesgos

Los riesgos de la participación en la investigación clínica se minimizarán mediante la restricción de los criterios de inclusión y exclusión, la obtención de la aprobación médica antes de la participación en el estudio y una estrecha vigilancia por parte de expertos clínicos durante todo el estudio. Todos los ensayos se llevarán a cabo en un entorno de rehabilitación en una superficie sólida, seca y plana. Cada participante será supervisado por personal entrenado durante cada sesión de entrenamiento. Durante cada sesión de entrenamiento se evaluará la integridad de la piel, los niveles de dolor, la presión sanguínea y la integridad de las articulaciones. Si existe algún problema con estos factores, o se tienen razones para creer que el paciente puede haber experimentado un evento adverso grave, el dispositivo será retirado y se proporcionará atención médica de inmediato. Cualquier evento adverso que ocurra se documentará tal y como se detalla en la **Sección 12** de este documento.

Se ha realizado un análisis de riesgo del producto sanitario en investigación de acuerdo con la ISO 14971:2019. Los resultados del análisis de riesgo se explican con más detalle en la **Sección 6.3** de este documento.

6.7 Justificación del balance positivo riesgo/beneficio

Por todo lo mencionado anteriormente, la relevancia de esta investigación está clínicamente justificada, ya que se centra en la mejora de la salud y estado funcional del paciente. Gracias a su simplicidad, el protocolo de la investigación clínica puede ser fácilmente reproducido en otros centros de rehabilitación de lesión medular.

Para más información consultar el Manual del Investigador (MI), sección 7.3 Riesgo residual general: análisis riesgo/beneficio.

6.8 Consideraciones especiales respecto a la pandemia de COVID-19

Tanto el promotor como los investigadores se comprometen a aplicar medidas especiales en relación con la pandemia de COVID-19:

- El riesgo general de la investigación clínica con respecto a la pandemia de COVID-19 es bajo. Además, se excluirán los pacientes mayores de 70 años y los pacientes con comorbilidades severas.
- Se documentarán los contactos entre investigadores y pacientes, a fin de tener una trazabilidad de los contactos en caso de que se produzca una infección.
- Se informa a los pacientes, a través de la hoja de información al paciente, de que la participación en la investigación clínica implica contactos adicionales con los empleados del hospital. Aunque se prestará atención al estricto cumplimiento de las normas de higiene, esto se asocia con un mayor riesgo de infección por COVID-19.

7. Objetivos del estudio e hipótesis

7.1 Objetivos

7.1.1 Objetivo principal

El objetivo principal de este estudio es comparar la eficiencia energética durante la marcha utilizando órtesis tipo KAFO (estándar de asistencia actual) con el dispositivo robótico ABLE Exoskeleton, en pacientes con lesión medular y en un entorno hospitalario.

7.1.2 Objetivos secundarios

Los objetivos secundarios son comparar el rendimiento de las órtesis tipo KAFO con el del dispositivo ABLE Exoskeleton mediante:

- Análisis cinemático de la marcha y parámetros espacio-temporales de la misma.
- Evaluación del tiempo y esfuerzo requerido para aprender a utilizar el dispositivo.
- Evaluación del impacto que la actividad física tiene en los participantes tras el entrenamiento de la marcha.
- Evaluación del nivel de satisfacción de los participantes.

Además de estos objetivos, se evaluará durante todo el estudio la seguridad del ABLE Exoskeleton como dispositivo de asistencia para la rehabilitación de la marcha en pacientes con LM.

7.1.3 Endpoints

El endpoint principal es determinar la eficiencia energética durante la marcha en pacientes con LM completa cuando se utiliza el dispositivo ABLE Exoskeleton y compararla con la eficiencia energética cuando se utilizan unas órtesis tipo KAFO.

Los endpoints secundarios consisten en comparar las órtesis tipo KAFO con el dispositivo ABLE Exoskeleton mediante la evaluación de:

- El análisis cinemático de la marcha y parámetros espacio-temporales de la misma.
- El tiempo y esfuerzo requeridos para aprender a utilizar el dispositivo.
- El impacto que la actividad física tiene en la salud de los participantes.
- La satisfacción de los usuarios con respecto al uso del dispositivo.

Estas valoraciones se realizarán durante la valoración preliminar y las sesiones de entrenamiento (ver **Sección 9** Medidas de resultados).

Además de estas valoraciones, y en relación con la valoración de la seguridad, se evaluará y notificará el número de eventos adversos graves (EAG), eventos adversos (EA) y abandonos causados por el dispositivo ABLE Exoskeleton.

7.1.4 Hipótesis

La hipótesis principal del estudio es que la marcha en pacientes con LM es más eficiente (menor consumo energético) cuando utilizan el dispositivo ABLE Exoskeleton que la marcha cuando utilizan órtesis pasivas tipo

KAFO, lo que promueve que tiendan a bipedestar y caminar períodos más largos en lugar de usar la silla de ruedas, con los beneficios que esto implicaría en su rehabilitación y en su salud.

Las hipótesis secundarias son:

- El dispositivo ABLE Exoskeleton mejora la cinemática y los parámetros espacio-temporales de la marcha (se asemejan más al patrón de marcha de un individuo sano), con respecto a las órtesis tipo KAFO.
- El dispositivo ABLE Exoskeleton tendrá un impacto más positivo en la movilidad y salud psicosocial de los participantes con LM del estudio, con respecto a las órtesis tipo KAFO.
- El dispositivo ABLE Exoskeleton es un dispositivo seguro para utilizar en un entorno hospitalario como dispositivo de asistencia durante la rehabilitación de la marcha en pacientes con LM.

7.2 Aplicaciones y beneficios previstos del producto sanitario en investigación a verificar

La aplicación prevista del producto sanitario es para la rehabilitación de la marcha de pacientes con LM en entornos clínicos. El beneficio del dispositivo es que permite una marcha más eficiente, cómoda y funcional que las órtesis tipo KAFO utilizadas actualmente en la terapia habitual de rehabilitación de esta patología. Por esto, podría ser ampliamente utilizado en hospitales y centros de rehabilitación.

8. Diseño del estudio

8.1 Diseño / Plan experimental

Se trata de un estudio unicéntrico cruzado aleatorizado. Los sujetos realizarán un periodo de entrenamiento de 5 semanas con uno de los 2 dispositivos (ABLE Exoskeleton o órtesis tipo KAFO) seleccionado aleatoriamente, seguido de una valoración post-entrenamiento que consta de 1 sesión. Una vez finalizado el test, los sujetos tendrán un periodo de descanso de 2 semanas después del cual repetirán el proceso con el otro dispositivo durante 5 semanas (seguido de 1 sesión de valoración post-entrenamiento). El estudio se realizará en el Hospital Asepeyo de Sant Cugat en España, donde se reclutará a un total de 10 pacientes.

Después de obtener el consentimiento informado, a través de la Hoja de Información al Paciente y el Consentimiento Informado, se llevará a cabo la selección de los pacientes.

Los pacientes que cumplan los criterios de inclusión y exclusión y pasen el procedimiento de selección, serán incluidos en el estudio. A continuación, los participantes se someterán a un programa de entrenamiento con ambos dispositivos. Se crearán dos grupos, cada uno de los cuales comenzará el periodo de entrenamiento con un dispositivo distinto: órtesis tipo KAFO o ABLE Exoskeleton.

Los participantes realizarán 2 sesiones de entrenamiento a la semana de una duración aproximada de 90 minutos durante 5 semanas con cada uno de los dispositivos, completando un total de 10 sesiones con ortésis tipo KAFO y 10 sesiones con ABLE Exoskeleton. Se realizarán valoraciones clínicas estandarizadas durante las sesiones 5 y 10 (sesiones de valoración). Durante las sesiones del período de entrenamiento también se realizarán distintas mediciones sobre el nivel de asistencia, variables espacio-temporales de la marcha y usabilidad. Una vez finalizado el periodo de entrenamiento se realizará una valoración post-entrenamiento que consta de 1 sesión dónde se medirá la capacidad física de los participantes. Tras finalizar el periodo de entrenamiento con uno de los dispositivos, los participantes tendrán un periodo de descanso de 2 semanas, después del cual cambiarán de dispositivo y repetirán todo el proceso. Cabe destacar que durante la valoración post-entrenamiento no se realizarán tareas con ninguno de los dispositivos y, por tanto, puede estar contenida en el periodo de descanso.

8.2 Productos en investigación y controles

8.2.1 Descripción de la exposición al producto sanitario en investigación

Los participantes utilizarán el dispositivo ABLE Exoskeleton durante la visita de selección para ponerse de pie con el objetivo de valorar si antropométricamente son aptos para poder utilizarlo. El tiempo aproximado de exposición durante esta prueba es de 10 minutos.

Además, los participantes realizarán un total de 10 sesiones de entrenamiento, cada una de 90 minutos de duración, con el dispositivo ABLE Exoskeleton.

8.2.2 Justificación de la selección de los controles

Como se trata de un estudio cruzado aleatorio, no se incluye ningún grupo de control.

8.2.3 Lista de otros dispositivos médicos o medicamentos que se utilizarán durante la investigación clínica

Según el nivel de asistencia que necesiten los participantes, se utilizarán otros dispositivos médicos como barras paralelas, andadores, andadores con ruedas o muletas mientras se camina con el dispositivo ABLE Exoskeleton o las órtesis tipo KAFO.

Se utilizará un analizador de gases portátil ($K4b^2$, COSMED Wearable Metabolic Systems) durante la valoración preliminar, las sesiones 5 y 10, y durante la sesión de post-entrenamiento. El analizador de gases portátil $K4b^2$ tiene sensores para detectar los niveles de oxígeno (O_2) y dióxido de carbono (CO_2) durante cada respiración (inhalación-exhalación), permitiendo así obtener métricas de los parámetros respiratorios y determinar el coste energético durante la actividad física. El analizador de gases portátil $K4b^2$ es un dispositivo médico de clase IIa que cuenta con el marcado CE. Además, en conjunto con el analizador portátil, se utilizará un filtro respiratorio antibacteriano (COSMED antibacterial filter A-182-300-004) para evitar la contaminación cruzada y mantener a salvo tanto a los participantes como a los operadores del sistema. El uso del analizador de gases portátil requiere de cierta experiencia y conocimientos técnicos previos. Por ello, el promotor designa al colaborador académico INEFC para llevar a cabo dicha actividad de forma adecuada (obtención de datos del analizador de gases, análisis de datos y soporte técnico), el cual aportará personal experto en el uso del dispositivo.

También se utilizará un ergómetro de brazos (Fluid E920 Medical UBE, First Degree Fitness) para realizar el GXT. El ergómetro de brazos Fluid E920 Medical UB es un dispositivo de entrenamiento y rehabilitación, tanto para atletas como para rehabilitación, principalmente para usuarios de sillas de ruedas. Los ergómetros o cicloergómetros de brazo son dispositivos similares a una bicicleta estática, pero, en este caso, el pedaleo se transmite a través de los brazos. El ergómetro de brazos Fluid E920 Medical UBE utiliza una resistencia ajustable por fluidos y permite el análisis de datos como la duración del ejercicio, la potencia o la cadencia de pedaleo. El dispositivo Fluid E920 Medical UBE es un dispositivo médico de clase I que cuenta con el marcado CE.

Como se describe en la **Sección 5.9**, se utilizarán órtesis de miembro inferior tipo KAFO como dispositivo comparador. Las órtesis tipo KAFO (órtesis de rodilla, tobillo y pie) son un producto sanitario que aparece en el catálogo común de prestación ortoprotésica del Ministerio de Sanidad, Consumo y Bienestar Social con código homologado 06 12 18 004, y se utilizan para inmovilizar la rodilla, permitiendo la bipedestación y la marcha.

8.3 Medidas que se llevan a cabo para minimizar el sesgo

Los participantes del estudio serán pacientes en tratamiento en el centro donde se desarrollará la investigación clínica. Los investigadores no pueden influir en las características de los pacientes admitidos para el tratamiento y, por lo tanto, no pueden causar un sesgo de selección. Cualquier paciente bajo tratamiento que cumpla los criterios de inclusión y exclusión, y que esté dispuesto a participar será incluido en el ensayo. La distribución de los grupos se realizará de forma aleatoria. Como no hay un grupo de control, no se requiere la inclusión de ciego en la investigación clínica.

Los participantes seleccionados para el estudio contarán con experiencia previa en el uso de órtesis tipo KAFO. Sin embargo, el tiempo desde la última vez que utilizaron una órtesis tipo KAFO es desconocido y puede variar. En cualquier caso, y para evitar sesgos, los participantes realizarán un periodo de entrenamiento, de misma duración, tanto con las órtesis tipo KAFO como con el dispositivo ABLE Exoskeleton. Los participantes realizarán el primer periodo de entrenamiento con uno de los dispositivos seleccionado de forma aleatoria. Una vez finalizado el primer periodo de entrenamiento, los participantes dispondrán de un periodo de descanso de 2 semanas que servirá para olvidar lo aprendido previamente. Es decir, habrá una pérdida en la calidad de las habilidades adquiridas. De esta forma, la habilidad inicial para deambular será similar en los dos periodos de entrenamiento.

Por último, cabe destacar que cada uno de los participantes utilizará su propio KAFO. Esto es así porque las órtesis tipo KAFO son dispositivos hechos a medida y, por tanto, no es posible adaptar un único KAFO a distintas morfologías corporales. Sin embargo, debido a los criterios de inclusión y el hecho de que los participantes serán pacientes que pertenecen al mismo centro hospitalario, las órtesis tipo KAFO serán similares y totalmente comparables (con el mismo código homologado del catálogo común de prestación ortoprotésica). De todas formas, se documentará el tipo de KAFO, su peso y su funcionalidad básica para poder justificar la similitud entre dispositivos.

8.3.1 Aleatorización

Para el presente estudio debemos asignar 10 participantes a dos alternativas de entrenamiento diferentes (ABLE Exoskeleton y órtesis tipo KAFO). El orden de entrenamiento se asignará mediante una asignación aleatoria simple. Para ello se utilizará el libro de números aleatorios producido por la Corporación RAND titulado *A Million Random Digits with 100,000 Normal Deviates*[52]. El proceso para determinar el orden de los participantes es el siguiente:

Al inicio de la investigación clínica (antes de empezar el reclutamiento), el Investigador Principal del estudio seleccionará una de las 20.000 líneas que contienen el millón de números aleatorios. Cada línea contiene 50 números aleatorios comprendidos entre el 0 y el 9, agrupados en 10 series de 5 cifras. Los 10 primeros números de la línea del libro seleccionada definirán el grupo de entrenamiento inicial para cada uno de los participantes: siendo el primer número para el primer participante, el segundo número para el segundo participante y así sucesivamente. Los números pares (0, 2, 4, 6 y 8) se destinarán al grupo que empieza el entrenamiento con el dispositivo ABLE Exoskeleton. Los números impares (1, 3, 5, 7 y 9) se destinarán al grupo que empieza el entrenamiento con la órtesis tipo KAFO.

8.4 Procedimientos del estudio

8.4.1 Reclutamiento de pacientes

Para el estudio se reclutarán 10 pacientes. Los abandonos no serán reemplazados y se asume una tasa de abandono del 20% durante la fase de entrenamiento. La tasa de abandono en la valoración de seguimiento podría ser mayor, se espera que sea de alrededor del 30%.

Los participantes serán reclutados utilizando una muestra de conveniencia. El reclutamiento debe completarse antes del 31.06.2021. Los potenciales participantes que reciben tratamiento en el centro de estudio serán identificados por el personal del estudio. Si un paciente accede a participar dando su consentimiento informado, el personal clínico será informado de la inclusión del paciente en el estudio.

8.4.2 Procedimiento de selección

El procedimiento de preselección anterior a recibir el consentimiento informado puede realizarse evaluando los registros médicos estándar para determinar si los sujetos pueden ser candidatos potenciales para el estudio. Las valoraciones realizadas exclusivamente para determinar la elegibilidad para este estudio se realizarán únicamente después de que se haya obtenido el consentimiento informado del paciente para la participación en el estudio.

Las valoraciones realizadas previamente durante la rutina clínica y documentadas en los registros de los pacientes pueden usarse como valores preliminares, aunque se hayan realizado antes de obtener el consentimiento informado. Se pedirá a los participantes en el estudio que den su consentimiento explícito para que los datos recogidos con propósito de tratamiento médico puedan usarse también para este estudio.

Se llevará a cabo un registro de selección. Los participantes que no cumplan con los criterios del estudio en el momento de la selección serán documentados como fracasos de selección. Se podría llegar a cabo otra sesión de selección posterior con pacientes en fase subaguda para comprobar si cumplen los criterios del estudio.

El procedimiento de selección será llevado a cabo por el médico o personal del estudio en el centro de investigación.

El procedimiento de selección deberá incluir los siguientes pasos:

- Consentimiento informado
- Comprobación de los criterios de inclusión/exclusión mediante la revisión de los antecedentes médicos pertinentes.
- Valoración del riesgo de fracturas por fragilidad siguiendo el criterio de Craven 2009[1].
- Documentación de datos demográficos (peso y altura).
- ISCoS Base de datos internacional de lesión medular 2.0.
- Valoración de la integridad de la piel.
- Rango de movilidad articular (ROM, siglas en inglés) de la cadera, rodillas y tobillos.
- Valoración de la espasticidad (Escala de Ashworth Modificada, EAM) en la cadera, rodillas y tobillos.
- Normas Internacionales para la Clasificación Neurológica de la Lesión Medular (ISNCSCI).
- WISCI II sin el exoesqueleto.
- Caminar 5 metros con el KAFO y la ayuda de un andador con ruedas.
- Documentar tipo de KAFO, peso y una breve descripción funcional básica del dispositivo de cada participante.
- Prueba de levantarse con el exoesqueleto, incluyendo valoración de síntomas de hipotensión ortostática

Durante este procedimiento de selección, los participantes serán introducidos en los conceptos básicos del uso del exoesqueleto y sus beneficios y riesgos relevantes, utilizando videos según sea necesario.

8.4.3 Procedimiento preliminar / Sesión 0

Los pacientes que pasen el procedimiento de selección (cribado) continuarán con el procedimiento de valoración preliminar, llevado a cabo, siempre que sea posible, durante la misma sesión. Los resultados obtenidos en el cribado respecto a ROM, EAM, ISNCSCI y WISCI II podrán ser utilizados si el tiempo entre el cribado y la valoración preliminar es inferior a una semana en los pacientes subagudos (<6 meses después de

sufrir la lesión). Si no es así, estas pruebas deberán repetirse. El procedimiento preliminar debe completarse 10 días antes de la sesión 1 como máximo.

En la valoración preliminar los participantes realizarán una prueba de ejercicio máximo gradual (GXT) utilizando un ergómetro manual (Fluid E920 Medical UBE, First Degree Fitness). Durante la prueba se analizará el intercambio de gases mediante un analizador de gases portátil ($K4b^2$, COSMED Wearable Metabolic Systems), se registrará la frecuencia cardíaca y la presión sanguínea y se valorará la fatiga percibida y el índice de esfuerzo percibido (RPE).

La **prueba de ejercicio máxima gradual** (**GXT**) es una herramienta de detección para rastrear el nivel de condición física de un individuo. La prueba evalúa la capacidad de ejercicio del participante midiendo la respuesta cardiovascular a la actividad física. A través de esta prueba se obtendrá el valor del consumo de oxígeno máximo que será utilizado para obtener las métricas para valorar la eficiencia energética durante la marcha.

La prueba se llevará a cabo de la siguiente manera[53–56]:

- Reposo en posición sentado durante 3 minutos.
- Calentamiento de 3 minutos con una resistencia de 0 vatios
- Inicio del test con una resistencia de 30 vatios.
- Aumento de la resistencia cada 2 minutos (15 vatios) hasta alcanzar el VO2peak. Los participantes deberán mantener una cadencia constante de 60-80 revoluciones/minuto
- Recuperación durante 3 minutos.

Se considerará que los pacientes han alcanzado el VO2peak cuando ocurran 2 de los siguientes 3 criterios:

- Estabilidad (plateau) en el VO2 a pesar del aumento en la resistencia del ergómetro manual.
- Ratio de intercambio respiratorio (RER) superior a 1.10
- Agotamiento voluntario cuando una cadencia mínima de 60 no se puede mantener

Una vez finalizada la prueba de ejercicio máxima gradual (GXT), los pacientes completarán el cuestionario de la Medida de Independencia en Lesión Medular (SCIM III) que permite evaluar diferentes aspectos de la vida diaria como son: actividades, coordinación, movilidad funcional, incontinencia y alimentación.

8.4.4 Procedimientos de entrenamiento

El participante utilizará el dispositivo correspondiente 2 veces por semana durante 90 minutos por sesión a lo largo de 5 semanas, completando un total de 10 sesiones con cada dispositivo. En caso de haber sesiones perdidas, el periodo de entrenamiento cuenta con una semana extra (semana 6) para poder recuperar las sesiones. Las sesiones perdidas también se pueden recuperar en cualquier momento durante las 5 semanas de entrenamiento, realizando 1 sesión a la semana de más (3 veces por semana), evitando realizar las 3 sesiones en días consecutivos. Las sesiones deben programarse para 90 minutos por sesión para permitir ajustes, tiempo para ponerse y quitarse el dispositivo y tiempo para el registro de datos/pruebas. El tiempo de terapia (sentado, de pie o caminando) debe ser de un mínimo de 30 minutos.

Cada sesión se lleva a cabo por un profesional entrenado y, si es necesario, por un terapeuta o asistente adicional.

Procedimientos de ponerse/quitarse el dispositivo

En cada sesión de entrenamiento, los participantes realizarán el procedimiento de ponerse y quitarse el dispositivo en cuestión (órtesis tipo KAFO o ABLE Exoskeleton). El terapeuta prestará asistencia sólo cuando sea necesario.

Actividades de la terapia

Durante cada sesión de entrenamiento, el participante realizará distintas actividades que irán aumentando su dificultad en función del nivel de asistencia requerido para desempeñarlas (Ver **Sección 9.2.2** Objetivos secundarios del estudio). Los participantes deberán practicar y completar las actividades en el orden establecido. Se avanzará a la siguiente actividad cuando el participante supere la actividad precedente con un nivel mínimo de asistencia (Ver **Sección 9.2.2** Objetivos secundarios del estudio).

El terapeuta documentará el nivel de asistencia (LoA; ver **Sección 9.2.2** Objetivos secundarios del estudio) tanto para los procedimientos de ponerse/quitarse el dispositivo como para las actividades a realizar. El participante irá completando las distintas actividades durante cada sesión. Se registrará el tiempo necesario (número de sesiones) para completar cada una de las actividades. Si el participante es incapaz de completar ninguna de las tareas, o el terapeuta considera que no es seguro intentar completarlas, entonces se registra como “No evaluable”.

El resto de la sesión de entrenamiento se llevará a cabo en el nivel que el terapeuta considere apropiado.

En cada sesión de entrenamiento deben registrarse los siguientes datos:

- Antes de cada sesión de entrenamiento:
  - Valoración de la integridad de la piel.
  - EVA y localización del dolor.
  - Se tomará la presión sanguínea sentado y de pie, si esto se identificó como un riesgo durante la selección, o si el participante ha mostrado síntomas de hipotensión ortostática en sesiones anteriores.
- Durante cada sesión de entrenamiento:
  - Tiempo en ponerse/quitarse el dispositivo.
  - Nivel de asistencia requerido para ponerse/quitarse el dispositivo.
  - Actividades de la terapia completadas.
  - Nivel de asistencia requerido para completar las actividades de la terapia.
- Al terminar cada sesión de entrenamiento:
  - Valoración de la integridad de la piel.
  - EVA y localización del dolor.
  - Registro de caídas o casi caídas.
  - Registro de cualquier necesidad de realizar un examen médico.
  - Registro de cualquier evento adverso.
- Será registrado automáticamente por el dispositivo ABLE Exoskeleton al final de cada sesión de entrenamiento:
  - Tiempo de pie en el dispositivo.
  - Tiempo de marcha en el dispositivo.
  - Número de pasos realizados.
  - Velocidad de la marcha
  - Distancia recorrida

Sesiones 5 y 10

Durante las sesiones 5 y 10 se registrará:

- TUG (dos veces).
- 10MWT.
- 6MWT.
- Frecuencia cardíaca.
- Presión sanguínea
- Valoración de la fatiga percibida mediante escala visual analógica (EVA)
- Valoración del RPE mediante la escala de Borg.
- Análisis de intercambio de gases
- Análisis cinemático de la marcha

8.4.4.1 Primera sesión de entrenamiento

En el día 1 del estudio, se formará a los participantes en las técnicas de ponerse/quitarse el dispositivo correspondiente. En caso de utilizar el ABLE Exoskeleton, un profesional entrenado llevará a cabo las mediciones y ajustes del exoesqueleto.

Los participantes serán formados en los modos de operación del exoesqueleto y guiados en el proceso de uso básico del dispositivo: levantarse, mantenerse de pie, transferir peso o sentarse; siempre utilizando muletas o las ayudas técnicas para caminar apropiadas para el paciente.

8.4.4.2 Sesiones 5 y 10

En las sesiones 5 y 10 de cada dispositivo se realizarán los siguientes test clínicos:

- TUG: El TUG se realizará dos veces para, posteriormente, obtener la media y desviación estándar.
- 6MWT + 10MWT: Se utilizarán los primeros 10 metros del 6MWT para realizar el test clínico 10MWT. En este caso el 6MWT se realizará una única vez.

La estructura de cada test es la siguiente:

- 3 minutos en reposo sentado
- Test correspondiente (TUG, 6MWT)
- 3 minutos de recuperación sentado

Cada test comenzará una vez los participantes hayan alcanzado los niveles de reposo y se hayan recuperado totalmente del test anterior.

Se realizará un análisis del intercambio de gases en cada uno de los test clínicos. Se obtendrá un análisis cinemático y parámetros espacio-temporales de la marcha durante los test. La frecuencia cardíaca y la presión sanguínea serán registradas durante la realización de los test. La percepción de la fatiga será valorada antes y después de cada test. El RPE se valorará al final de cada test y el PCI se calculará únicamente para el 6MWT.

Durante la realización de los test se tomarán medidas cinemáticas, se obtendrán parámetros espacio-temporales de la marcha y el coste energético de la marcha. Si el participante es incapaz de completar uno o más test, o el terapeuta considera que no es seguro intentar completarlos, entonces se registra como “No evaluable”.

La obtención de datos cinemáticos de la marcha (registro de datos y soporte técnico) corre a cargo de un colaborador académico del promotor, el Laboratorio de Ingeniería Biomecánica (BIOMEC) de la Universitat Politècnica de Catalunya (UPC), quien cuenta con una amplia experiencia y conocimiento en la biomecánica de la marcha y aportará personal experto en el uso del sistema de captura del movimiento. Para realizar las capturas del movimiento del sujeto se utilizarán cámaras infrarrojas (NaturalPoint OptiTrack Flex 3). Estas cámaras infrarrojas son capaces de detectar el movimiento de marcadores especiales, adheridos al participante bajo estudio, con gran precisión a una escala submilimétrica. Las cámaras se utilizan conjuntamente con el software Motive (también de Optitrack). Los datos obtenidos de las capturas se exportan a OpenSim (un software de modelización, simulación y de análisis biomecánico) o a Matlab (un entorno de computación numérica) para desarrollar el análisis dinámico y simulaciones.

8.4.4.3 Sesiones perdidas

En caso de haber sesiones perdidas, el periodo de entrenamiento cuenta con una semana extra (semana 6) para poder recuperarlas. Las sesiones podrán redistribuirse durante la semana extra de manera que el paciente pueda completar todas las sesiones y en el orden pertinente. Las sesiones perdidas también se pueden recuperar en cualquier momento durante las 5 semanas de entrenamiento, realizando 1 sesión a la semana de más (3 veces por semana), evitando siempre realizar las 3 sesiones en días consecutivos. Las sesiones de entrenamiento deben realizarse siempre antes de iniciar la valoración post-entrenamiento. No se considerarán como abandonos los pacientes que no puedan completar las 10 sesiones de entrenamiento previstas.

8.4.5 Procedimientos post-entrenamiento

La valoración post-entrenamiento debe completarse como máximo 10 días después de la sesión final y consiste en una única sesión de un tiempo aproximado de 45 minutos. Cabe destacar que durante la valoración post-entrenamiento no se realizarán tareas con ninguno de los dispositivos y, por tanto, puede estar contenida en el periodo de descanso.

Durante esta sesión los participantes del estudio realizarán una prueba de ejercicio máxima gradual (GXT) utilizando un ergómetro portátil, un analizador de gases clínico, monitorización de la frecuencia cardíaca y presión sanguínea para medir su respuesta cardiorrespiratoria.

La prueba se realizará siguiendo la misma estructura que durante la valoración preliminar (ver **Sección 8.4.3** Procedimiento preliminar). El intercambio de gases será medido constantemente durante la prueba. La frecuencia cardíaca (FC), la presión sanguínea (PS) y la fatiga serán valorados durante el reposo, el ejercicio y la recuperación. El RPE será valorado al final de la prueba.

Una vez finalizada la prueba de ejercicio máxima gradual (GXT), los pacientes completarán el cuestionario SCIM III con el objetivo de valorar cualquier mejora que el periodo de entrenamiento haya podido tener en la vida diaria de los pacientes. Además del SCIM III, los participantes completarán los cuestionarios QUEST 2.0 (Evaluación de Quebec de la Satisfacción de Usuarios con Tecnología de Asistencia) y PIADS (Escala del Impacto Psicosocial de Productos de Apoyo) para evaluar el grado de satisfacción del usuario y el impacto psicosocial que haya podido tener el entrenamiento con el dispositivo pertinente. Para una explicación detalla de los cuestionarios QUEST 2.0 y PIADS ver **Sección 9.2** Medidas de resultados.

8.4.6 Procedimiento de descanso

El periodo de descanso tiene lugar entre la finalización del primer periodo de entrenamiento y el inicio del segundo periodo de entrenamiento. El tiempo del periodo de descanso es de 2 semanas. Con este periodo de descanso se pretende reducir las habilidades adquiridas y competencias para la deambulación de forma que la habilidad inicial al inicio de cada uno de los periodos sea lo más similar posible.

En el periodo de descanso los participantes no podrán utilizar ninguno de los dispositivos durante la terapia. Sin embargo, los participantes sí podrán realizar la valoración post-entrenamiento dentro del periodo de descanso, ya que no requiere del uso de ninguno de los dispositivos de asistencia de la marcha (órtesis tipo KAFO y ABLE Exoskeleton)

8.4.7 Procedimientos de seguimiento

Cuatro semanas (± 1 semana) después de la finalización de la valoración post-entrenamiento del segundo periodo*, se llevará a cabo un examen de seguimiento mediante una entrevista telefónica con los participantes para evaluar los efectos a largo plazo relacionados con el uso de los dispositivos.

Esto incluye:

- PIADS
- Se anotará cualquier evento adverso notificado entre la finalización del programa de entrenamiento y la visita de seguimiento.

**El estudio consta de dos valoraciones post-entrenamiento, cada una al final del periodo de entrenamiento de cada uno de los dispositivos. Por lo tanto, el procedimiento de seguimiento se realizará una única vez cuando se hayan finalizado los dos periodos.*

8.4.8 Medidas finales

Al final del estudio los terapeutas participantes completaran el QUEST 2.0 para obtener información sobre sus opiniones respecto el uso del dispositivo ABLE Exoskeleton en un entorno hospitalario.

8.4.9 Diagrama de flujo del estudio

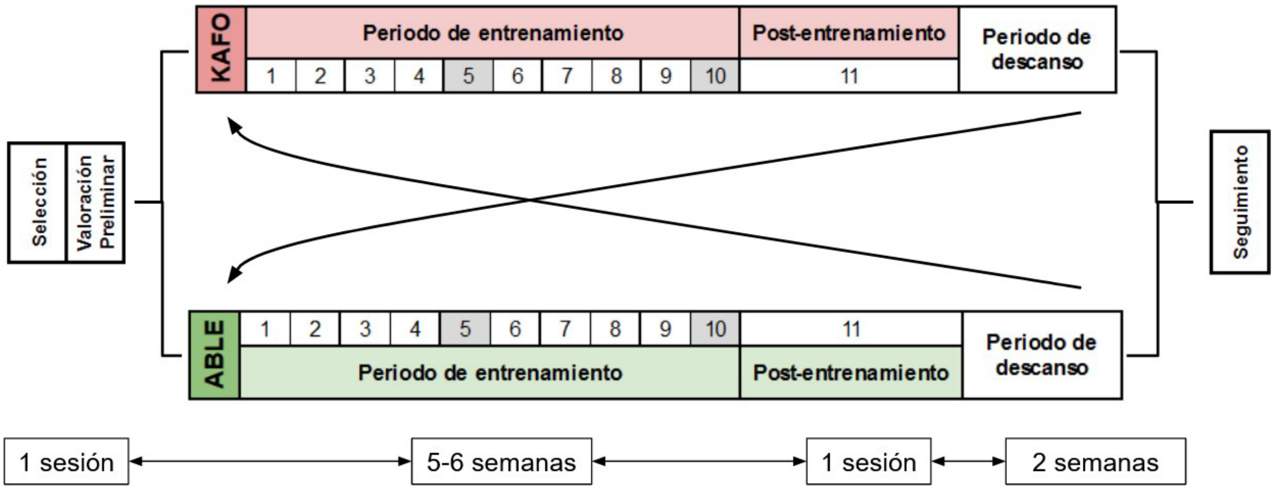

Figura 6: Diagrama de flujo de la investigación clínica

9. Medidas de resultados

9.1 Recogida de datos generales

Los datos generales del paciente incluirán datos demográficos y el historial médico del paciente.

El conjunto internacional de datos básicos sobre lesiones medulares (ISCoS 2.0) es una colección estandarizada de información de pacientes con lesión medular. Documenta datos demográficos tales como el género, fechas de nacimiento, lesión, admisiones agudas y de rehabilitación. Otra información recogida de ISCoS 2.0 son la etiología y tipo de lesión, asistencia ventilatoria, residencia actual y datos neurológicos de la admisión aguda[57].

Datos demográficos adicionales al ISCoS que se recogerán para este estudio son información sobre el peso y altura de los participantes.

Para evaluar el estado neurológico se utilizan las Normas Internacionales para la Clasificación Neurológica de la Lesión Medular (ISNCSCI). Las normas ISNCSCI incluyen un examen sensorial y motor y una clasificación llamada escala de discapacidad ASIA (AIS) [58].

Se medirá el Rango de Movimiento (ROM) de las extremidades inferiores para los abductores y aductores de la cadera, los flexores y extensores de la cadera, los flexores y extensores de la rodilla, y los dorsiflexores y plantares del tobillo.

Se analizará la movilidad funcional de la marcha a través de la valoración WISCI II, la cual valora la cantidad de asistencia física necesaria para caminar, así como las ayudas requeridas.

La escala de Ashworth modificada (EAM) se utilizará para determinar la espasticidad en los abductores y aductores de la cadera, flexores y extensores de cadera, flexores y extensores de la rodilla y flexores plantares y dorsiflexores del tobillo. La escala EAM mide la resistencia durante el estiramiento muscular pasivo en una escala con 6 categorías[59]:

- EAM 0: No hay aumento del tono muscular.
- EAM 1: Ligero aumento del tono muscular visible con la palpación o relajación, o por una mínima resistencia al final del rango de movimiento cuando la(s) parte(s) afectada(s) se mueve(n) en flexión o extensión.
- EAM +1: Aumento ligero del tono muscular, visible con la palpación, seguido de resistencia mínima en el resto (menos de la mitad) del rango de movimiento.
- EAM 2: Aumento más pronunciado del tono muscular en la mayoría del rango de movimiento, pero la parte afectada se mueve con facilidad.
- EAM 3: Aumento considerable del tono muscular, con movimiento pasivo difícil.
- EAM 4: La parte afectada está rígida en flexión o extensión.

Otra información que se documentará en relación con la **historia médica** de los pacientes son las comorbilidades existentes y los riesgos de fractura por fragilidad según Craven et al. (2009)[1]. Según Craven et al. (2009), 5 o más de los siguientes criterios determinan un alto riesgo de fracturas por fragilidad en pacientes con lesión medular:

- Consumo de alcohol > 5 vasos/día
- Índice de masa corporal (IMC) < 19
- Duración de la lesión medular >= 10 años
- Mujer

- Motora completa (AIS A-B)
- Paraplejia
- Previa fractura por fragilidad
- Historia familiar de fracturas

9.2 Medidas de resultados

9.2.1 Objetivos principales del estudio

Se tomarán las siguientes medidas en línea con el objetivo principal del estudio de evaluar la eficiencia energética durante la marcha cuando se camina con órtesis tipo KAFO y compararla con el dispositivo ABLE Exoskeleton en investigación.

Para medir el coste energético necesario durante la marcha se analizará el intercambio de gases a través de un analizador de gases clínico (K4b^2, COSMED Wearable Metabolic Systems).

El análisis del intercambio de gases se hará durante los siguientes test clínicos normalizados:

La **prueba de ejercicio máxima gradual** (**GXT**) es una herramienta de detección para rastrear el nivel de condición física de un individuo. La prueba evalúa la capacidad de ejercicio del participante midiendo la respuesta cardiovascular a la actividad física. A través de esta prueba se obtendrá el valor del consumo de oxígeno que se utilizará para obtener las métricas para valorar la eficiencia energética.

El test **"levántate y anda" (TUG)** mide el tiempo que una persona necesita para levantarse de una silla, caminar 3 metros, girar y volverse a sentar. Es un test ampliamente usado para evaluar el equilibrio y el riesgo de caídas en grupos de pacientes diferentes[60].

El test de **marcha de 10 metros (10MWT)** mide el tiempo que una persona necesita para caminar 10 metros. El test se realiza con un inicio dinámico con aceleración de 2 metros, una distancia cronometrada de 10 metros y una desaceleración de 2 metros[61,62]. Cabe destacar que el 10MWT se realizará aprovechando los primeros 10 metros del test de marcha de 6 minutos.

El test de **marcha de 6 minutos** (**6MWT**) mide la distancia que una persona puede caminar en 6 minutos. Existen distintas posibilidades para la realización del test. Para este estudio utilizaremos una pista de, como mínimo, 10 metros, donde los pacientes caminan de ida y de vuelta[61,62].

Estas medidas se aplicarán en las sesiones 5 y 10 del periodo de entrenamiento.

9.2.2 Objetivos secundarios del estudio

Se realizarán las siguientes mediciones en línea con los objetivos secundarios del estudio para evaluar la cinemática de la marcha, el tiempo y esfuerzo requeridos para aprender a utilizar los dispositivos, el impacto físico y psíquico que tiene el entrenamiento en los participantes, valorar el grado de satisfacción de los participantes con los dispositivos y evaluar la seguridad del dispositivo ABLE Exoskeleton

Para el análisis de la marcha se realizarán dos sesiones de valoración (sesión 5 y 10) en las que se realizará un estudio cinemático de la marcha. Se obtendrán métricas como la inclinación de la pelvis, la trayectoria del pie o la inclinación del tronco, entre otras. Además, durante todas las sesiones en las que se utilice el dispositivo ABLE Exoskeleton, se registrará el tiempo de pie, el tiempo de marcha y el número de pasos realizados. Estas métricas se obtienen directamente a través del dispositivo ABLE Exoskeleton.

Para medir el esfuerzo necesario para aprender a utilizar el dispositivo en investigación, se registrará la frecuencia cardíaca y la presión sanguínea en las sesiones 5 y 10 del periodo de entrenamiento mientras se realizan distintos test clínicos estandarizados. También se registrará la fatiga percibida y RPE en los distintos test clínicos. El RPE se medirá a través de la escala de BORG antes y después de cada test. La escala BORG mide el nivel subjetivo de intensidad de trabajo físico en una escala de 15 grados. Va de 6 (ningún esfuerzo) hasta 20 (esfuerzo máximo) [63].

Los test clínicos normalizadas son los siguientes:

El test **"levántate y anda" (TUG)** mide el tiempo que una persona necesita para levantarse de una silla, caminar 3 metros, girar y volverse a sentar. Es un test ampliamente usado para evaluar el equilibrio y el riesgo de caídas en grupos de pacientes diferentes[60].

El test de **marcha de 10 metros (10MWT)** mide el tiempo que una persona necesita para caminar 10 metros. El test se realiza con un inicio dinámico (volando) con aceleración de 2 metros, una distancia cronometrada de 10 metros y una desaceleración de 2 metros[61,62]. Cabe destacar que el 10MWT se realizará aprovechando los primeros 10 metros del test de marcha de 6 minutos

El test de **marcha de 6 minutos** (**6MWT**) mide la distancia que una persona puede caminar en 6 minutos. Existen distintas posibilidades para la realización del test. Para este estudio utilizaremos una pista de 10 metros, donde los pacientes caminan de ida y de vuelta[61,62].

Para medir el tiempo requerido para aprender a utilizar el dispositivo en investigación se realizarán diferentes actividades durante el periodo de entrenamiento (**Tabla 1**). La dificultad de cada actividad aumenta a medida que se consiguen completar. Para pasar a la siguiente actividad los participantes deben completar la actividad previa con un nivel mínimo de asistencia de "Asistencia Mínima (Min)" (**Tabla 2**). Se llevará un registro del número de sesiones y LoA necesario para completar cada actividad. Las diferentes actividades a realizar durante el periodo de entrenamiento se definen a continuación (**Tabla 1**):

Tabla 1: Habilidades a realizar durante el periodo de entrenamiento

Categoría	Orden	Habilidad
**Ponerse/quitarse el dispositivo**	-	Ponerse el dispositivo
	-	Quitarse el dispositivo
**Habilidades de equilibrio**	1	Levantarse
	2	Cambio de peso (adelante/atrás, izquierda/derecha)
	3	Manipular el control remoto estando de pie
	4	Tocarse la cabeza mientras está de pie
	5	Sentarse
**Habilidades para caminar**	6	Caminar 10 metros (con paradas)
	7	Empezar y parar la marcha con la pierna preferida
	8	Empezar y para la marcha con la pierna no-preferida
	9	Caminar 10 metros (sin parar)

	10	Girar sobre sí mismo
**Habilidades avanzadas**	11	Caminar cerca de una silla, girar y sentarse
	12	Caminar una curva de 90º hacia la derecha (con paradas)
	13	Caminar una curva de 90º hacia la derecha (sin paradas)
	14	Caminar una curva de 90º hacia la izquierda (con paradas)
	15	Caminar una curva de 90º hacia la izquierda (sin paradas)
	16	Para la marcha cerca de un objeto y manipularlo
	17	Caminar por una zona estrecha
	18	Parar delante de una puerta, abrirla (hacia fuera) y seguir caminando
	19	Parar delante de una puerta, abrirla (hacia dentro) y seguir caminando
	20	Parar la marcha cerca de una pared, girar y apoyarse sobre ella (poder descansar sin ayuda)
	21	Caminar en diferentes superficies (alfombra, esterilla de yoga, …)
	22	Caminar por un eslalon

Los niveles de asistencia se definen como se muestra en la tabla siguiente (**Tabla 2**):

Tabla 2: Definición de los niveles de asistencia (LoA) en el proceso de ponerse/quitarse el dispositivo y para realizar las actividades de terapia

LoA	Ponerse/Quitarse el dispositivo	Actividades de terapia
**Asistencia Total (Tot)**	El participante realiza entre 0-25% del esfuerzo requerido para ponerse/quitarse el exoesqueleto.<br><br>El participante depende esencialmente del terapeuta para llevar a cabo todos los aspectos del proceso de ponerse/quitarse el dispositivo.	El participante realiza entre el 0-25% del esfuerzo requerido para usar el exoesqueleto.<br><br>Dos terapeutas son requeridos para dar soporte al participante en el uso del dispositivo en todo momento.
**Asistencia máxima (Max)**	El participante realiza entre el 25-50% del esfuerzo requerido para ponerse/quitarse el dispositivo.<br><br>El participante necesita máxima asistencia para colocarse el dispositivo y posicionar las piernas, pero es capaz de ajustar las cintas de los muslos.	El participante realiza entre el 25-50% del esfuerzo requerido para usar el exoesqueleto.<br><br>El participante necesita asistencia máxima del terapeuta para mantener el equilibrio.

**Asistencia Moderada (Mod)**	El participante realiza entre el 50-75% del esfuerzo requerido para ponerse/quitarse el dispositivo.<br>El participante necesita asistencia moderada para colocarse el dispositivo y posicionar las piernas, pero es capaz de ajustarse las cintas de los muslos y pantorrillas.	El participante realiza entre el 50-75% del esfuerzo requerido para usar el exoesqueleto.<br><br>El terapeuta tiene las dos manos en el participante o en el dispositivo en todo momento para, de forma ocasional, guiar o dar soporte para mantener el equilibrio.
**Asistencia Mínima (Min)**	El participante realiza > 75% del esfuerzo requerido para ponerse/quitarse el dispositivo.<br><br>El participante puede colocarse el dispositivo y ajustar las cintas, pero puede necesitar ayuda para posicionar las piernas.	El participante realiza 75% o más del esfuerzo requerido para usar el exoesqueleto.<br><br>El terapeuta tiene una mano en el participante o el dispositivo para, de forma no frecuente, guiar o dar soporte para mantener el equilibrio.
**Supervisión (S)**	El terapeuta no toca al participante, pero puede dar indicaciones verbales o mantener el contacto con el paciente para garantizar la seguridad.	El terapeuta no toca al participante, pero está cerca para proporcionar soporte para mantener el equilibrio o guía según se requiera.
**Independencia (I)**	El participante es completamente independiente para ponerse/quitarse el dispositivo.	El participante es completamente independiente mientras utiliza el dispositivo y el terapeuta no proporciona ningún tipo de asistencia.

Para medir el impacto que la actividad física tiene en los participantes se realizará una prueba de ejercicio máxima gradual (GXT; ver **Sección 9.2.1** Objetivos principales del estudio) previa al periodo de entrenamiento que se comparará con la GXT que se realizará en la valoración post-entrenamiento (ver **Sección 8.4.5** Valoración post-entrenamiento) para comparar su respuesta cardiorrespiratoria. Durante las sesiones en las que se realiza el GXT se realiza también el formulario SCIM III, cuyos resultados se compararán para ver el efecto que el periodo de entrenamiento ha tenido en la independencia de la vida diaria de los pacientes.

Para evaluar el grado de satisfacción del usuario y el impacto psicosocial, se realizarán las siguientes pruebas:

La **Evaluación de Quebec de la Satisfacción de Usuarios con Tecnología de Asistencia (QUEST 2.0)** está diseñada para medir el nivel de satisfacción y el valor que las personas atribuyen a las tecnologías de asistencia. Para ello, utiliza 12 variables las cuales se puntúan en una escala de 5 puntos en términos de importancia y satisfacción percibidas. Mientras que las variables 1-8 valoran la satisfacción con el producto, las variables 9-12 se utilizan para valorar la satisfacción con el servicio. Si los usuarios no están muy satisfechos con una determinada característica, se les pide que proporcionen información específica. La puntuación final es la suma de puntuaciones promedio de todas las respuestas válidas en un rango de 1 (nada satisfecho) a 5 (muy satisfecho) [64,65].

La **Escala del Impacto Psicosocial de Productos de Apoyo (PIADS)** es un cuestionario de 26 preguntas (escala de 7 puntos), diseñado para evaluar los efectos de un dispositivo de asistencia en la independencia funcional, el bienestar y la calidad de vida. Se divide en tres subescalas: competencia, adaptabilidad y

autoestima. Para cada una de las subescalas se calculan típicamente medias que van de -3 (máximo impacto negativo) a +3 (máximo impacto positivo). Para tener solo puntuaciones positivas, el rango de las puntuaciones individuales se desplaza de 1 a 7, lo que significa que las puntuaciones sumadas van de 1*26 (26, máximo impacto negativo) a 7*26 (182, máximo impacto positivo). La escala PIADS es una medida que responde y es sensible a variables importantes como la condición clínica del usuario, el estigma y las características funcionales del dispositivo. Se ha demostrado que refleja con precisión las experiencias autodescritas de las personas que utilizan dispositivos de asistencia[66,67].

Además de las medidas presentadas, se tomarán otras medidas en línea con la seguridad de los dispositivos, guardando especial cuidado durante el uso del dispositivo ABLE Exoskeleton.

Se hará un examen de la piel antes y después de cada sesión utilizando el **Sistema Internacional de clasificación de las Úlceras por Presión NPUAP/EPUAP[68]:**

- Categoría/Estadio I: Eritema no blanqueable
- Categoría/Estadio II: Perdida parcial del grosor de la piel
- Categoría/Estadio III: Perdida total del grosor de la piel
- Categoría/Estadio IV: Pérdida total del espesor de los tejidos
- Inclasificable: Profundidad desconocida
- Sospecha de lesión en los tejidos profundos: profundidad desconocida.

Después de cada sesión, se informará de la ocurrencia de caídas o casi caídas, así como cualquier supuesto evento adverso relacionado con el dispositivo que requiera un examen médico. Además, se registrarán el cambio en el nivel de dolor utilizando una escala analógica visual (EVA) y las ubicaciones del cuerpo.

9.3 Calendario para la valoración, el registro y el análisis de las variables

Tabla 3: Calendario para la valoración, el registro y el análisis de las variables

	Selección	Valoración preliminar	Sesión 1-final	Sesiones 5 y 10	Post-entrenamiento	Medidas finales	Seguimiento
Consentimiento Informado	X						
Criterios de inclusión/exclusión	X						
ISNCSCI	X						
ISCoS 2.0	X						
Demografía	X						
Historia Médica Pasada y medicación Relevante	X						
Introducción al exoesqueleto	X						

ROM en extremidades inferiores	X						
Documentación KAFO	X						
Prueba con el KAFO	X						
Prueba de levantarse con el exoesqueleto	X						
Espasticidad (EAM) en extremidades inferiores	X						
Valoración del riesgo de fracturas por fragilidad	X						
WISCI II	X						
Revisión de la piel	X		X*	X*			
SCIM III		X			X		
EVA y localización del dolor			X*	X*			
Frecuencia de caídas o casi-caídas			X	X			
Tiempo y LoA para ponerse/quitarse el dispositivo			X	X			
LoA para las actividades de terapia			X				
Fatiga (EVA)		X		X	X		
RPE (Escala de BORG)		X		X	X		
Frecuencia cardíaca		X		X	X		
Presión sanguínea		X		X	X		
Examen médico necesario / Eventos adversos		X	X	X	X		X
Datos recogidos por el exoesqueleto			X	X			
Datos cinemáticos (captura del movimiento)				X			
GXT		X			X		
TUG				X			
10MWT				X			
6MWT				X			
PIADS					X		X

QUEST 2.0					X	X	
Análisis de gases		X		X	X		

* Evaluado antes y después de la sesión.

10. Población de pacientes

10.1 Número de pacientes a reclutar

Un total de 10 pacientes que cumplan los criterios de inclusión/exclusión serán reclutados para la investigación. Se incluirán en el estudio los participantes con LM completa o incompleta. Los participantes serán preseleccionados por el personal del estudio. Una vez se haya identificado a un potencial participante, el médico del estudio proporcionará al paciente un consentimiento informado escrito y verbal.

Se mantendrá un registro de preselección.

10.2 Duración de la participación individual en el estudio

La duración total de la participación de cada individuo será de aproximadamente 16 semanas, incluyendo el reclutamiento y el seguimiento. El estudio finalizará con la valoración del periodo de seguimiento del último paciente incluido en el estudio.

10.3 Criterios de inclusión y exclusión

10.3.1 Criterios de inclusión

Cada individuo debe cumplir los requisitos siguientes para ser incluido en el estudio:

- 18 a 70 años de edad.
- Lesión medular crónica o subaguda (puede ser de origen traumático o no traumático).
- Actualmente en tratamiento como paciente hospitalizado o ambulatorio en el centro de investigación.
- De AIS A a AIS D con suficiente fuerza en los brazos para soportar el peso corporal en un andador.
- Experiencia previa caminando con órtesis tipo KAFO (deben tolerar la bipedestación).
- Capacidad de dar su consentimiento informado

10.3.2 Criterios de exclusión

- WISCI II sin exoesqueleto > 16.
- 5 o más factores de riesgo por fragilidad según lo publicado por Craven et al[1].
- Historia de fracturas por fragilidad de los miembros inferiores en los últimos 2 años.
- Deterioro > 3 puntos del total en la puntuación motora de las Normas Internacionales para la Clasificación Neurológica de la Lesión Medular (ISNCSCI) en las últimas 4 semanas.
- Inestabilidad espinal.
- Escala de Ashworth modificada (EAM) > 3 en extremidades inferiores.
- Incapaz de tolerar 30 minutos de pie sin síntomas clínicos de hipotensión ortostática.
- Incapaz de caminar 5 metros con órtesis tipo KAFO y la ayuda de un andador con ruedas.
- Problemas psicológicos o cognitivos que no permitan seguir los procedimientos del estudio.
- Cualquier condición neurológica que no sea LM.
- Médicamente inestable.

  - Sistema Cardiovascular inestable, inestabilidad hemodinámica, hipertensión no tratada (PSS>140, PSD> 90 mmHg), trombosis venosa profunda (TVP), disreflexia autonómica no controlada (AD).
- Comorbilidades severas.
  - Cualquier condición que el médico considere no apropiada para completar la participación en el estudio.
- Problemas de la piel en curso.
  - Grado I o superior en EPUAP en áreas que vayan a estar en contacto con el exoesqueleto[33].
- Altura, anchura, peso u otras limitaciones anatómicas (como las diferencias de longitud de las piernas) incompatibles con el dispositivo.
- Rango de movimiento (ROM) insuficiente para el dispositivo ABLE Exoskeleton.
- Embarazo o lactancia conocidos.

10.4 Criterios para la retirada de sujetos

10.4.1 Suspensión del tratamiento

El investigador puede suspender temporal o permanentemente la participación del paciente en el estudio por cualquier razón en el mejor interés para el paciente, particularmente en los casos de EAGs y si existe preocupación por la seguridad o el incumplimiento con los procedimientos del estudio. Si un paciente no responde a la visita de seguimiento, el investigador utilizará todos los medios disponibles para ponerse en contacto con él/ella. Después de tres recordatorios telefónicos semanales, el paciente será registrado como perdido para el seguimiento. El investigador completará el motivo del abandono o finalización anticipada en el cuaderno de recogida de datos.

10.4.2 Retirada de la investigación clínica

Los participantes pueden retirarse de la investigación clínica en cualquier momento y por cualquier motivo. Esta decisión no tiene ningún impacto en su atención médica. En caso de terminación anticipada, el investigador debe documentar las razones de la forma más completa posible.

10.5 Duración total prevista de la investigación clínica

El estudio se llevará a cabo del 01.01.2021 hasta el 31.08.2021. Sería posible una prórroga hasta el 31.10.2021, si a 31.08.2021 faltaran las valoraciones de seguimiento de más del 25% de los participantes del estudio. Posteriormente se realizará un análisis descriptivo con respecto a los objetivos primarios y secundarios del estudio.

10.6 Duración prevista del reclutamiento de pacientes

Los pacientes serán reclutados para participar en el estudio desde el 01.01.2021 hasta tener un máximo de 10 participantes inscritos en el estudio. La fecha límite para la inclusión en el estudio será el 31.06.2021.

10.7 Apoyo médico a los participantes de la investigación clínica

La investigación clínica tiene lugar en un hospital con servicio de rehabilitación especializado en el tratamiento médico y quirúrgico y la rehabilitación integral de personas con lesión medular. Todos los tratamientos médicos necesarios pueden realizarse en el centro de investigación. Durante el examen de seguimiento, se cuestionan los problemas de salud pasados y presentes. En caso de problemas de salud actuales, el centro de investigación dispone de un servicio de atención de urgencias, por lo que los pacientes estarán totalmente atendidos y podrán tener un seguimiento médico.

No hay requisitos especiales de tratamiento o atención médica. Si un paciente experimenta problemas antes o después del examen de seguimiento, puede acudir al departamento ambulatorio del centro de investigación en todo momento. Las historias clínicas de los pacientes contendrán una nota que informará a los médicos de la participación del paciente en la investigación del dispositivo ABLE Exoskeleton.

11. Estadística

11.1 Análisis estadístico previsto

Para todas las características demográficas y clínicas, se utilizará estadística descriptiva. Las variables cuantitativas se resumirán utilizando estadística descriptiva estándar (promedio, desviación estándar, mediana, mínimos y máximos). Las variables cualitativas, incluidas los eventos adversos, se describirán utilizando los tamaños y frecuencias de los grupos. Se presentarán las características de los pacientes en el momento de su inclusión en el estudio.

Los resultados primarios y secundarios se analizan de la siguiente forma:

- Analizando y comparando el intercambio de gases durante la marcha a través de un analizador de gases ($K4b^2$, COSMED Wearable Metabolic Systems) mientras se realizan test clínicos normalizados con el dispositivo ABLE Exoskeleton y con órtesis tipo KAFO.
- Comparando el esfuerzo y fatiga de la marcha mediante test clínicos normalizados con el dispositivo ABLE Exoskeleton y con órtesis tipo KAFO durante las sesiones 5 y 10 del periodo de entrenamiento.
- Comparando el análisis cinemático y parámetros espacio-temporales de la marcha con el dispositivo ABLE Exoskeleton y con órtesis tipo KAFO durante las sesiones 5 y 10 del periodo de entrenamiento.
- Midiendo el LoA y el tiempo necesario para completar las distintas actividades del programa de entrenamiento con el dispositivo ABLE Exoskeleton.
- Comparando la capacidad física al inicio y al final de cada periodo de entrenamiento.
- Comparando los resultados del QUEST 2.0 y el PIADS cuando el periodo de entrenamiento se ha llevado a cabo con el dispositivo ABLE Exoskeleton y con órtesis tipo KAFO.
- Registrando el número y gravedad de los eventos adversos que pudieran ocurrir durante el uso del dispositivo ABLE Exoskeleton.

Las diferencias entre estas medidas de resultados se analizarán utilizando una prueba t de Student apareada o el test no paramétrico de Wilcoxon si no se consigue la normalidad y homogeneidad de varianzas. Los resultados serán considerados como estadísticamente significativos cuando p=< 0,05. Para evaluar el tiempo de utilizar el dispositivo ABLE Exoskeleton, se analizará el tiempo promedio para alcanzar las actividades de terapia específicas, ponderado con los niveles LoA invertidos (100% - LoA).

Los campos de respuesta abierta del QUEST 2.0 y el PIADS serán evaluados usando métodos cualitativos. Con codificaciones, se identificarán y analizarán temas importantes.

Cualquier desviación respecto a los procedimientos mencionados anteriormente será documentada en el informe del estudio.

11.2 Tamaño de muestra

El tamaño de muestra será de 10 participantes con lesión medular.

El tamaño de la muestra (10 pacientes) de la presente investigación clínica se fija teniendo en cuenta los antecedentes de otras investigaciones clínicas con el objetivo de comparar el rendimiento de un exoesqueleto robótico con el dispositivo estándar actual para la asistencia de la marcha en personas con LM (órtesis tipo KAFO)[16,23,31,32], o con el objetivo de medir el coste energético[69–71] o esfuerzo[45] que requiere utilizar un exoesqueleto robótico. Se incluye información detallada sobre estudios similares a esta investigación clínica en la **Sección 4.1**.

Para determinar una mejoría en la marcha con uno de los dispositivos respecto al otro se van a tener en cuenta diferentes variables (cuantitativas, cualitativas y cuestionarios). Sin embargo, la variable principal para valorar la mejoría de la marcha será el consumo de oxígeno medido a través del analizador de gases portátil ($K4b_2$, COSMED Wearable Metabolic Systems). Se utilizarán pruebas estadísticas como la prueba t de Student o el test no paramétrico de Wilcoxon para valorar si la diferencia de consumo de oxígeno entre ambos dispositivos es significativa.

Cabe destacar que el objetivo principal de este estudio es comparar la calidad de la marcha (capacidad de deambulación), usando un dispositivo frente al otro, en pacientes con lesión medular. En ningún caso este estudio pretende demostrar superioridad clínica (cambios clínicamente significativos en la salud de los participantes) de un dispositivo respecto al otro, ya que para ello sería necesario un estudio con cientos de personas.

11.3 Gestión de datos ausentes, inutilizados o falsos

No hay consideración ni atribución de los valores perdidos. Los resultados inesperados o incorrectos serán revisados durante la revisión final antes del cierre de la base de datos.

El análisis estadístico se realizará sobre la población con intención a tratar (AIT). La población del AIT se define como los pacientes inscritos en el estudio, independientemente del tratamiento recibido o sus resultados obtenidos en el estudio.

11.4 Tasas de abandono previstas

Se registrarán las razones de abandono siempre que sea posible. Se estudiarán las características de los pacientes que se pierdan durante el seguimiento. Se espera una tasa de abandono del 20%.

12. Gestión de eventos adversos

12.1 Definiciones

ABLE Human Motion S.L. clasificará cada Evento Adverso de acuerdo con la norma ISO 14155:2011. En caso de que las definiciones nacionales y las regulaciones de notificaciones sean más estrictas que las exigidas por la ISO 14155:2011, la notificación se hará en cumplimiento de las regulaciones específicas del país.

El investigador es responsable de registrar todos los eventos adversos diagnosticados en los exámenes durante el estudio, notificados por el participante o comunicados al investigador por una tercera persona durante el período del estudio.

Evento Adverso, EA (ISO 14155:2011 3.2)

Un Evento Adverso es cualquier episodio médico no deseado, enfermedad o lesión no prevista, o signos clínicos no deseados (incluyendo los hallazgos de laboratorio anormales) en sujetos, usuarios u otras personas, ya estén o no relacionados con el producto en investigación.

NOTA 1 Esta definición incluye los eventos relacionados con los procedimientos utilizados.

NOTA 2 Para los usuarios u otras personas incluyendo los terapeutas, esta definición está restringida a eventos relacionados con el dispositivo médico en investigación.

Una enfermedad o síntoma preexistente no se considerará un evento adverso a menos que haya un cambio adverso en su intensidad, frecuencia o calidad. Este cambio será documentado por un investigador. Los procedimientos quirúrgicos en sí mismos no son EAs, son medidas terapéuticas para las condiciones que requieren cirugía. La condición para la cual se requiere la cirugía puede ser un EA. Todos los EAs (incluyendo los EAGs) serán documentados en un formulario de EAs. Los EA se clasificarán como “no graves” o “graves” (ver detalles más adelante).

Evento adverso grave, EAG (ISO 14155:2011 3.37)

Evento adverso que:

a) Dio lugar a una muerte;

b) Dio lugar a un deterioro grave de la salud del sujeto que:

- Produjo una enfermedad o lesión con riesgo de pérdida de la vida, o
- Produjo una deficiencia permanente de una estructura corporal o una función corporal, o
- Precisó la hospitalización del paciente o la prolongación de la hospitalización existente, o
- Precisó una intervención médica o quirúrgica para impedir una enfermedad con riesgo de pérdida de la vida o lesión o deficiencia permanente de una estructura corporal o una función corporal;

c) Dio lugar a angustia fetal, muerte fetal o a una anomalía congénita o un defecto de nacimiento.

NOTA No se considera un evento adverso grave la hospitalización planificada para una condición preexistente, o un procedimiento requerido por el PIC, sin deterioro grave del estado de salud.

Efecto Adverso del producto, EAP (ISO 14155:2011 3.1)

Evento adverso relacionado con la utilización del producto sanitario en investigación.

NOTA 1 Esta definición incluye eventos adversos que resulten de la insuficiencia o inadecuación de las instrucciones de uso, implementación, implantación, instalación, o utilización, o de cualquier mal funcionamiento del producto sanitario en investigación.

NOTA 2 Esta definición incluye cualquier evento adverso que resulte de un error de utilización o del mal uso intencionado del producto sanitario en investigación.

Efecto adverso grave del producto, EAGP (ISO 14155:2011 3.36)

Efecto adverso del dispositivo que ha resultado en cualquiera de las consecuencias características de un evento adverso grave.

Efecto adverso grave inesperado del producto, EAGIP (ISO 14155:2011 3.42)

Efecto adverso grave del producto que por su naturaleza, incidencia, intensidad o consecuencias no ha sido identificado en la versión actualizada del informe de análisis de riesgo.

NOTA Un efecto adverso grave esperado del producto (EAGEP) es un efecto que por su naturaleza, incidencia, intensidad o consecuencias ha sido identificado en el informe de análisis de riesgos.

Deficiencia del producto (ISO 14155:2011 3.15)

Inadecuación de un producto sanitario con respecto a su identidad, calidad, durabilidad, fiabilidad, seguridad o prestaciones.

NOTA Las deficiencias del producto incluyen los fallos de funcionamiento, los errores de utilización, y la inadecuación del etiquetado.

12.2 Descripción de posibles eventos adversos y efectos adversos del producto en investigación

Ver **Sección 6.2**.

12.3 Detección y registro

El promotor implementará y mantendrá un sistema para asegurar que la notificación de los eventos notificables, tal como se definen en la **Sección 12.4**, sea realizada por el investigador al promotor inmediatamente, no más tarde de 3 días naturales después de que el personal del estudio conozca el evento.

La información de Eventos adversos (EA) será recopilada a lo largo del estudio y notificada a ABLE Human Motion S.L. en el formulario de Eventos adversos del eCRD. Todos los Eventos adversos deben ser documentados. El investigador es el responsable de documentar todos los EA.

Para aquellos EA que requieran notificación inmediata, la notificación inicial puede realizarse por teléfono, correo electrónico (los datos de contacto se proporcionan en el archivo del centro de investigación) o en el eCRD con toda la información disponible. En caso de que el investigador requiera información del promotor en una situación de emergencia, los detalles de contacto para situaciones de emergencia se detallan en el archivo del centro de investigación.

Todos los EA deben ser registrados una sola vez en las páginas apropiadas para EA del eCRD. El investigador deberá completar todos los detalles solicitados, incluidas las fechas de inicio, gravedad, medidas correctivas (terapias correctivas administradas), resultado y relación (opinión sobre si el EA está relacionado con el producto sanitario o no), ver más detalles a continuación. Cada evento debe registrarse por separado.

En el caso de un EAG o deficiencias del producto sanitario en investigación (ver sección 12.4), el investigador debe completar, firmar y fechar las páginas de EAG del eCRD, comprobar que los datos son coherentes y enviar una copia en un plazo de un día hábil al contacto especificado:

ABLE Human Motion S.L.
Diagonal 647, 4ª Planta (CREB), Despacho 4.30
08028 Barcelona (España)
Email: amas@ablehumanmotion.com

Cuando se requiera información adicional de seguimiento, ésta deberá completarse en un formulario de seguimiento del EAG, y se enviará una copia al promotor (por email al contacto indicado arriba) y el original se colocará en la sección de EAG del eCRD.

12.4 Eventos adversos que requieren notificación

Debe ser notificado:

- Cualquier EAG

- Deficiencias del producto que no condujeron a un evento adverso pero que podrían haber llevado a una incidencia médica:
    a) Si no se hubiera tomado ninguna medida adecuada,
    b) Si no se hubiera intervenido, o
    c) Si las circunstancias hubieran sido menos afortunadas.
- Nuevos hallazgos/actualizaciones en relación con eventos ya notificados.
- Todos los eventos que requieren la notificación del promotor a las autoridades competentes.

La notificación se realiza mediante eCRD de Eventos adversos.

12.5 Características de eventos adversos

Cada evento debe ser clasificado teniendo en cuenta las siguientes características:

a) Severidad: El investigador debe evaluar la intensidad del modo siguiente:
    i) Leve: Signos y síntomas que pueden tolerarse fácilmente. Los síntomas son ignorados o desaparecen cuando el sujeto está distraído.
    ii) Moderada: Los síntomas causan malestar, pero son tolerables. No pueden ignorarse y afectan a la actividad normal.
    iii) Severa: Síntomas afectan en gran medida a la actividad normal.
b) Medidas correctivas: Las medidas correctivas aplicadas a un EA se asignan a una de las categorías siguientes:
    i) No: no se llevan a cabo medidas correctivas.
    ii) Sí: Medicamentos recientemente recetados u otras medidas correctivas, por ejemplo, un procedimiento quirúrgico.
c) Resultado:
    i) Recuperado/resuelto: todos los signos y síntomas de un EA han desaparecido sin dejar secuelas en la última visita.
    ii) No recuperado/no resuelto: Los signos y síntomas de un EA no han cambiado o han empeorado en la última visita.
    iii) Recuperado/resuelto con secuelas: Los signos y síntomas del EA han desaparecido, pero existen secuelas relacionadas con el EA.
    iv) Fatal: Causa la muerte. Si hay más de un evento adverso, solamente el evento adverso que ha causado la muerte debe caracterizarse como fatal.
    v) Desconocido: El resultado es desconocido o inverosímil y la información no puede suplementarse o verificarse.
d) Relación: Se evalúa la relación entre en EA y las causas potenciales. Las causas potenciales son:
    i) Producto en investigación.
    ii) Procedimiento médico
    iii) Enfermedad subyacente
    iv) Otros

    La relación debe clasificarse de acuerdo con los criterios siguientes:
    - Relacionado: Hay una posibilidad razonable de que el evento haya sido causado por el producto sanitario en investigación/procedimiento médico. Un determinado evento tiene una fuerte relación temporal y es poco probable que haya una causa alternativa.
    - Probable: Un EA que tiene una posibilidad razonable de haber sido causado por el producto sanitario en investigación/procedimiento médico. El EA tiene una relación oportuna y sigue un patrón de respuesta conocido, pero puede existir una posible causa alternativa.
    - Posible: Un EA que tiene una posibilidad razonable de haber sido causado por el producto sanitario en investigación/procedimiento médico. El EA tiene una relación oportuna con el producto sanitario en investigación/procedimiento médico; sin embargo, el patrón de repuesta

es atípico, y parece más probable una causa alternativa, o existe una incertidumbre significativa sobre la causa del evento.

- Improbable: Sólo existe una conexión remota entre el producto sanitario en investigación/procedimiento médico y el evento adverso reportado. Otras condiciones, como una enfermedad concurrente, la progresión o la expresión del estado de la enfermedad o la reacción de la medicación concomitante, parecen explicar el evento adverso reportado.
- No relacionado: Un EA que no sigue una secuencia temporal razonable relacionada con el producto sanitario en investigación/procedimiento médico y es probable que se haya producto por un estado clínico del sujeto, otros modos de terapia u otra etiología conocida.

La clasificación de la relación será llevada a cabo conjuntamente por el investigador responsable y el promotor.

e) Previsión: La clasificación de las expectativas de ocurrencia debe ser realizada por el promotor de acuerdo con las siguientes definiciones:
   i) Anticipada: Un EA que por su naturaleza, incidencia, gravedad o resultado ha sido identificado en el análisis de riesgo.
   ii) Imprevisto: Un evento inesperado es aquel que por su naturaleza, incidencia, gravedad o resultado no ha sido identificado en la versión actual del análisis de riesgo.

Todos los EAGs serán sujetos a una segunda valoración por una persona designada por el promotor, quien será independiente del investigador responsable.

El segundo asesor, rellenará una segunda hoja de evaluación para cada EAG y la enviará por correo electrónico al promotor (amas@ablehumanmotion.com), en un plazo de 48 horas.

La segunda hoja de evaluación contendrá la siguiente información:

- Evaluación de la relación entre el EAG y el producto sanitario en investigación/procedimiento médico.
- Evaluación de la relación entre el EAG y la enfermedad subyacente.
- Evaluación de la previsión (expectativa de ocurrencia) del EAG (derivada del Manual del Investigador, Instrucciones de Uso, u otro documento apropiado).

La notificación de EAGs a las Autoridades Nacionales Competentes se llevará a cabo por el Promotor de acuerdo con los requerimientos nacionales específicos.

12.6 Notificación del promotor a las autoridades nacionales competentes (ANCs)

El promotor de la investigación clínica debe notificar a la Agencia Española del Medicamento y Producto Sanitario (AEMPS) todos los eventos adversos graves, relacionados o no con el producto sanitario en investigación, tanto si se producen en España como en otros Estados y tanto si han ocurrido en la investigación clínica autorizada o en otras investigaciones clínicas o en un contexto de uso diferente, siempre que estos dispositivos médicos no se comercialicen en España. Las notificaciones se realizarán por correo electrónico (psinvclinic@aemps.es).

El plazo máximo de notificación será de 15 días naturales a partir del momento en que el promotor haya tenido conocimiento del evento adverso grave. Cuando el evento adverso grave haya ocasionado la muerte del sujeto, o puesto en peligro su vida, el promotor informará a la AEMPS en el plazo máximo de 7 días naturales a partir del momento en que el promotor tenga conocimiento del caso. Dicha información deberá ser completada, en lo posible, en los 8 días siguientes.

Cuando los eventos adversos ocurran en un ensayo clínico con doble ciego, se deberá desvelar el código de tratamiento de ese paciente concreto a efectos de notificación. Siempre que sea posible, se mantendrá el carácter ciego para el investigador, y para las personas encargadas del análisis e interpretación de los resultados, así como de la elaboración de las conclusiones del estudio.

El promotor debe implementar y mantener un sistema para garantizar que el investigador notifica al promotor los eventos notificables dentro del plazo de tiempo requerido, no más tarde de 15 días después de tener conocimiento de ellos.

El promotor utilizará esta plantilla para notificar a las autoridades competentes:

- A los órganos competentes de las Comunidades Autónomas en cuyo territorio se esté realizando la investigación, de forma individual y en el plazo máximo de 15 días, todos los eventos adversos graves, tanto si tienen relación con el producto en investigación como si no la tienen y que hayan ocurrido en pacientes seleccionados en sus respectivos ámbitos territoriales. Este plazo máximo será de 7 días cuando se trate de eventos adversos graves que produzcan la muerte o amenacen la vida.

- A los Comités Éticos que hayan autorizado la investigación clínica, de forma individual y en el plazo máximo de 15 días, todos los eventos adversos graves que hayan ocurrido en pacientes seleccionados en sus respectivos ámbitos. Este plazo máximo será de 7 días cuando se trate de eventos adversos graves que produzcan la muerte o amenacen la vida.

El promotor debe notificar cualquier otra información sobre los eventos adversos graves cuando así lo requiera el Comité Ético, el organismo competente de la Comunidad Autónoma o la AEMPS en el momento que se autorice el estudio y, en cualquier caso, si la información da lugar a un cambio significativo en la seguridad del producto investigado.

13. Monitorización

La monitorización del estudio será llevada a cabo por el promotor, ABLE Human Motion S.L. El objetivo de la monitorización es garantizar la supervisión adecuada del estudio, prestando especial atención a la verificación de todos los requisitos clínicos, la adherencia al protocolo, las buenas prácticas clínicas y el cumplimiento de los reglamentos gubernamentales e institucionales aplicables.

La monitorización se realizará mediante visitas in situ y externas, y mediante comunicación frecuente (cartas, teléfono, correo electrónico) por parte de ABLE Human Motion S.L. El monitor (ABLE Human Motion S.L.) visitará el centro de investigación a intervalos regulares, para verificar el cumplimiento del PIC y de los requisitos legales locales, para realizar la verificación de los datos fuente y para dar soporte al investigador en sus actividades relacionadas con el estudio. En total, se prevén cuatro visitas (in situ o remotas, en función de la situación de la pandemia de COVID-19 en ese momento) para cada centro de investigación: una visita de iniciación, una visita después del reclutamiento de los cinco primeros pacientes, una tercera visita poco después de la mitad del ensayo, y una cuarta visita para realizar el cierre de la investigación.

El monitor documentará en el registro de desviaciones del protocolo, como mínimo, las desviaciones del protocolo de los procedimientos del consentimiento informado, las desviaciones en la notificación de los EAGs en los plazos requeridos y la manipulación del producto sanitario en investigación. El registro de desviaciones del protocolo será completado durante el curso de la investigación como apéndice del informe de visita de monitorización.

El monitor es responsable de revisar que los datos estén completos y sean claros. Los detalles se definen en el Plan de Monitorización. Los investigadores se comprometen a permitir que el promotor realice auditorías y a permitir las posibles inspecciones por parte de las autoridades competentes. Todos los datos, documentos e informes pueden ser sujetos a auditorias e inspecciones.

13.1 Acceso directo a datos / documentos fuente

El investigador permitirá la supervisión del estudio, las auditorías, la revisión por parte del Comité de Ética e inspecciones por parte de las autoridades competentes. El investigador proporcionará acceso al promotor, a representantes autorizados del promotor como auditores, y a las autoridades competentes a los datos primarios (por ejemplo, datos fuente) que respalden los datos recogidos en los eCRDs de la investigación clínica, por ejemplo, diagramas de práctica generales, notas del hospital, libros de citas, registros originales de laboratorio.

Cualquier parte que tenga acceso a los registros de la investigación clínica deberá tomar todas las precauciones razonables, de conformidad con los requisitos reglamentarios aplicables, para mantener la confidencialidad de la información de identificación del paciente y la información confidencial y/o propiedad del promotor.

13.2 Documentos Fuente y Datos Fuente (De acuerdo con la norma ISO 14155:2011)

Los documentos Fuente se definen como documentos impresos, ópticos o electrónicos que contienen datos fuente (por ejemplo, registros hospitalarios, notas de laboratorio, registros de dispensación del producto, negativos fotográficos, radiografías, registros mantenidos en el centro de investigación, en los laboratorios y en los departamentos médico-técnicos involucrados en la investigación clínica).

Los datos fuente se definen como toda la información en los registros originales, copias certificadas de los registros originales de los resultados clínicos, observaciones, u otras actividades en una investigación clínica, necesarias para la reconstrucción y evaluación de la investigación clínica.

14. Colaboradores académicos del promotor

El promotor designa a 2 colaboradores académicos para llevar a cabo actividades específicas de la investigación clínica:

- **Laboratorio de Ingeniería Biomecánica (BIOMEC) de la Universitat Politècnica de Catalunya (UPC).** El Laboratorio de Ingeniería Biomecánica (BIOMEC) es un grupo de investigación de la Universitat Politècnica de Catalunya (UPC) y una de las siete áreas del Centro de Investigación en Ingeniería Biomédica (CREB). Cuenta con una amplia experiencia y conocimiento científico en la biomecánica de la marcha, concretamente en el desarrollo de modelos biomecánicos multicuerpo para analizar y simular la dinámica del movimiento humano, para aplicaciones clínicas y deportivas. En la presente investigación clínica, BIOMEC-UPC se encargará de la obtención de datos cinemáticos de la marcha (registro de datos y soporte técnico) en las sesiones de entrenamiento 5 y 10, y de liderar el análisis de los resultados y la redacción de los artículos científicos que se publicaran al finalizar la investigación clínica. BIOMEC-UPC aportará personal experto en el uso del sistema de captura del movimiento y divulgación científica de resultados.
- **Institut Nacional d’Educació Física de Catalunya (INEFC).** INEFC es un centro de enseñanza superior creado por la Generalitat de Catalunya que tiene como misión la formación, especialización y perfeccionamiento de graduados y graduadas en Educación Física y Deporte, así como la investigación científica y la divulgación de sus trabajos y estudios para la formación. En la presente investigación clínica, INEFC se encargará de la obtención de datos del analizador de gases y su posterior análisis (y el soporte técnico que se pudiera necesitar) durante la valoración preliminar, las sesiones de entrenamiento 5 y 10, y la sesión de post-entrenamiento. INEFC aportará personal experto en el uso del analizador de gases para que los datos y su análisis se hagan de forma adecuada.

15. Gestión de datos

15.1 Procedimientos para la recopilación de datos

El centro de investigación es responsable de la recogida de datos personales relacionados con el estudio. Se compromete a cumplir con las normas legales de protección de datos. Para la recolección de datos de los registros médicos, los participantes del estudio aceptan expresamente liberar a sus médicos de sus obligaciones de confidencialidad al respecto.

Los datos recopilados durante el estudio se documentarán en un Cuaderno de Recogida de Datos electrónico (eCRD). Los eCRDs se identificarán únicamente mediante un código (seudonimizados), de manera que no se incluya información que pueda identificar al sujeto. El centro de investigación conservará una lista escrita o electrónica de los datos personales de los participantes relacionados con los códigos de seudonimización. Sólo podrán acceder a ella el investigador principal y el personal del estudio y se almacenará en un lugar protegido del acceso de terceros. Por consiguiente, no se revelará la identidad de un paciente a nadie, salvo en el caso de emergencia sanitaria o un requisito legal.

La gestión de datos se realizará de acuerdo con los procedimientos normalizados de trabajo de ABLE Human Motion S.L. y el plan de gestión de datos para esta investigación clínica.

15.2 Datos fuente

Los investigadores deben mantener registros de la historia clínica de cada sujeto, la exposición al producto sanitario en investigación y el seguimiento clínico. Los documentos fuente incluyen los archivos hospitalarios del sujeto (electrónicos o en papel). El investigador marcará claramente los registros clínicos para indicar que el sujeto está inscrito en esta investigación clínica.

Los datos fuente deben ser mantenidos por los investigadores para respaldar los datos recogidos en los Cuadernos de Recogida de Datos electrónicos. En la siguiente tabla se resumen las características específicas para la recogida de cada tipo de datos fuente.

Tabla 4: Características específicas para la recogida de cada tipo de datos fuente

Variable	Registro	Modo de registro	Propósito	Relaciones esperadas
Consentimiento informado	Selección	Documento para cada participante	CI/CE (1)	No se espera relación
Introducción al exoesqueleto	Selección	Documento para cada participante	CI/CE (1)	No se espera relación
Edad	Preselección	Promedio (DS(2), rango)	CI/CE (1) Demografía	Variable que puede estar relacionada con el progreso.
Género	Preselección	Nº de personas en cada categoría	Demografía	No se espera relación.
Tiempo desde la lesión (meses)	Preselección	Promedio (DS(2), rango)	CI/CE Demografía	Variable que puede estar relacionada con el progreso.

Causa de la Lesión	Preselección	Tipos genéricos, número de personas	CI/CE Demografía	Variable que puede estar relacionada con el progreso.
Altura (cm)	Preselección y selección	Promedio (DS(2), rango)	CI/CE Demografía	Variable que puede estar relacionada con el progreso.
Peso (kg)	Preselección y selección	Promedio (DS(2), rango)	CI/CE Demografía	Variable que puede estar relacionada con el progreso.
Historia médica pasada relevante	Preselección y selección	Tipos genéricos, número de personas	CI/CE Demografía	Variable que puede estar relacionada con el progreso.
Medicaciones relevantes	Selección	Nombres genéricos, número de personas	CI/CE Demografía	Variable que puede estar relacionada con el progreso.
Valoración del riesgo de fracturas por fragilidad	Selección	Documentar el número de factores para cada participante.	CI/CE Demografía	No se espera relación.
ROM para extremidades inferiores	Selección	Documentar para cada participante	CI/CE	No se espera relación.
Valoración de espasticidad (EAM) en extremidades inferiores	Selección	Documentar para cada participante	CI/CE	No se espera relación.
Prueba con el KAFO	Selección	Documentar el resultado por participante	CI/CE	No se espera relación.
Prueba de levantarse con el exoesqueleto ABLE	Selección	Documentar el resultado por participante	CI/CE	No se espera relación.
ISCoS Base de datos internacional de lesión medular 2.0	Selección	Documentar para cada participante.	CI/CE Estado de los participantes	No se espera relación más allá del parámetro ISNCSCI.

Normas Internacionales para la Clasificación Neurológica de la Lesión Medular (ISNCSCI)	Selección, Valoración post-estudio	Documentar para cada participante, documentar cualquier cambio.	CI/CE<br>Estado de los participantes	Mejora potencial al llegar al final de la terapia.
WISCI II	Selección	Documentar para cada participante, documentar cualquier cambio.	CI/CE<br>Demografía<br>Estado de los participantes	Mejora potencial al llegar al final de la terapia.
SCIM III	Valoración preliminar, valoración post-estudio	Documentar para cada participante, documentar cualquier cambio.	CI/CE<br>Estado de los participantes	Mejora potencial al llegar al final de la terapia.
Integridad de la piel (EPUAP)	Selección, cada sesión	Documentar para cada participante, frecuencia y severidad de cambios en la piel.	CI/CE<br>Estado de los participantes	Intacta durante todo el estudio.
Incidencia de caídas o casi-caídas	Cada sesión	Reportar la frecuencia, naturaleza y severidad de las incidencias.	Estado de los participantes	No caídas.
Eventos adversos con necesidad de examen médico	Cada sesión, seguimiento	Reportar el número total de incidencias, frecuencia y severidad.	Estado de los participantes	No incidencias relacionadas con el producto sanitario.
EVA dolor	Cada sesión	Reportar cualquier cambio	Estado de los participantes, seguimiento de la respuesta	Sin cambios.
EVA fatiga	Valoración preliminar, sesiones 5 y 10, valoración post-entrenamiento	Reportar cualquier cambio. Promedio (DS[2], rango) para cada variable.	Estado de los participantes, seguimiento de la respuesta	Mejora potencial al llegar al final de la terapia.

Presión sanguínea y síntomas de hipotensión ortostática	Valoración preliminar, sesiones 5 y 10, valoración post-entrenamiento. Cada sesión de si se indica	Promedio (DS(2), rango) para cada variable.	Estado de los participantes, seguimiento de la respuesta	Mejora potencial al llegar al final de la terapia.
Frecuencia cardíaca	Valoración preliminar, sesiones 5 y 10, valoración post-entrenamiento	Promedio (DS(2), rango) para cada variable.	Estado de los participantes, seguimiento de la respuesta	Mejora potencial al llegar al final de la terapia.
LoA y tiempo para ponerse/quitarse el dispositivo	Cada sesión	Promedio (DS(2), rango) del tiempo necesario. Categorizar LoA en las sesiones.	Progresión	Reducción de LoA y tiempo al llegar a la sesión final.
LoA para las actividades de “Uso del dispositivo”	Cada sesión	Informe del logro de cada marcador en los bloques de entrenamiento.	Progresión	Reducción de LoA al llegar a la sesión final.
GXT	Valoración preliminar y valoración post-entrenamiento	Promedio (DS(2), rango) de las métricas obtenidas	Progresión y monitorizar la respuesta	Aumento del estado físico al final del periodo de entrenamiento y mayor eficiencia energética cuando se utiliza el dispositivo ABLE Exoskeleton
10MWT	Sesiones 5 y 10 del periodo de entrenamiento	Promedio (DS(2), rango) del tiempo necesario para todos los participantes.	Progresión	Menor tiempo necesario al llegar al final de la terapia y menor tiempo cuando se utiliza el dispositivo ABLE Exoskeleton

6MWT	Sesiones 5 y 10 del periodo de entrenamiento	Promedio (DS[(2)], rango) de la distancia caminada por todos los participantes.	Progresión	Incremento de la distancia al llegar al final de la terapia y mayor distancia cuando se utiliza el dispositivo ABLE Exoskeleton
TUG	Sesiones 5 y 10 del periodo de entrenamiento	Promedio (DS[(2)], rango) del tiempo necesario para todos los participantes.	Progresión	Menor tiempo necesario al llegar al final de la terapia y menor tiempo cuando se utiliza el dispositivo ABLE Exoskeleton
RPE	Valoración preliminar, sesiones 5 y 10 del periodo de entrenamiento y valoración post-entrenamiento	Promedio (DS[(2)], rango) de la puntuación de todos los participantes.	Progresión	Puntuación mejorada al llegar al final del entrenamiento y menor puntuación cuando se utiliza el dispositivo ABLE Exoskeleton
QUEST 2.0	Valoración post-entrenamiento y medidas finales	Promedio (DS[(2)], rango) de las puntuaciones reportadas.	Monitorizar la respuesta	Mejorado al llegar al final del estudio y mejor cuahndo se utiliza el dispositivo ABLE Exoskeleton
PIADS	Valoración post-entrenamiento, seguimiento	Promedio (DS[(2)], rango) de la puntuación de todos los participantes.	Monitorizar la respuesta	Opinión positiva reportada y más positiva cuando se utiliza el dispositivo ABLE Exoskeleton

1) CI= Criterios de inclusión. CE= Criterios de exclusión
2) SD= Desviación estándar

15.3 Cuadernos de Recogida de Datos (CRDs)

El Investigador Principal o su representante firmará los eCRDs completos. Los eCRDs serán monitorizados de acuerdo con las normas de Buena Práctica Clínica (BPC).

15.4 Revisión y procesamiento de datos

Los datos recogidos se examinarán a través de la monitorización para comprobar que sean completos, correctos y coherentes. El monitor pedirá al investigador que complete, corrija o comente los datos si fuera necesario.

Tanto el centro de investigación como el promotor son responsables del tratamiento de los datos personales y se comprometen a cumplir la normativa vigente sobre protección de datos, en concreto el Reglamento (RE) 2016/679 del Parlamento Europeo y del Consejo de 27 de abril de 2016 relativo a la protección de las personas físicas en lo que respecta al tratamiento de datos personales y a la libre circulación de estos datos (Reglamento General de Protección de Datos, RGPD) y la Ley Orgánica 3/2018, de 5 de diciembre, de Protección de Datos Personales y garantía de los derechos digitales.

Las obligaciones de la coadministración en materia de protección de datos se establecen de forma transparente en un acuerdo de conformidad con el Art. 26 DEL REGLAMENTO SOBRE LA PROTECCIÓN DE DATOS.

Los datos importantes para el estudio serán almacenados, evaluados y, si es necesario, transferidos a ABLE Human Motion S.L. y a los editores en una forma pseudonimizada, posiblemente también a países en los cuales los requisitos de protección de datos son más bajos que en la Unión Europea. Los datos personales proporcionados a estos terceros incluyen el código de pseudonimización, año de nacimiento, número de semanas después de la lesión y datos clínico-neurológicos (por ejemplo, nivel de la lesión, fuerza de los músculos de los brazos y piernas y severidad de la LM), datos funcionales sobre la independencia general y la capacidad de caminar, cuestionarios sobre la satisfacción con la vida y la usabilidad del exoesqueleto o la órtesis tipo KAFO, y datos sobre la utilización del exoesqueleto registrados por el dispositivo ABLE Exoskeleton (métricas de marcha, progresión, datos de uso de sensores y actuadores). Los participantes en el estudio serán informados acerca de esto por escrito antes de participar en el estudio y están explícitamente de acuerdo con estas regulaciones.

Los datos se utilizarán para los fines de este estudio y para futuras investigaciones sobre lesión medular.

15.5 Periodo de retención previsto

Los documentos esenciales de la investigación clínica, tal y como se definen en la norma ISO 14155:2011, deben ser conservados por el investigador durante la investigación clínica y durante el período que sea más largo entre el requerido por los requisitos reglamentarios aplicables o durante al menos 10 años después de la finalización prematura o la terminación de la investigación clínica. Sin embargo, el investigador deberá ponerse en contacto con el promotor antes de destruir cualquier registro o informe relativo a la investigación clínica para asegurarse de que ya no es necesario conservarlos. Además, se deberá contactar con el promotor si el investigador tiene previsto abandonar el centro para que se pueda gestionar la transferencia de los registros.

Los expedientes médicos de los pacientes en estudio deben conservarse de acuerdo con la legislación local y con el periodo máximo permitido por el hospital, institución o centro privado.

El promotor y el investigador principal deben tomar medidas para prevenir la destrucción accidental o prematura de estos documentos. El investigador principal o el promotor pueden transferir la custodia de los registros a terceras partes y documentar la transferencia en el centro de investigación o en las instalaciones del promotor.

15.6 Derechos de los pacientes en materia de protección de datos

De acuerdo con la normativa legal, los participantes pueden ejercer sus derechos de acceso, modificación, oposición y cancelación de datos, así como el tratamiento de datos incorrectos, solicitar una copia de sus datos o solicitar la transferencia de sus datos a un tercero.

No obstante, el consentimiento para la recopilación y el tratamiento de datos personales, en particular la información sobre la salud, es irrevocable. En caso de revocación para participar en el ensayo clínico, los datos almacenados hasta ese momento podrán seguir utilizándose en la medida en que sea necesario para:

a) lograr o no comprometer seriamente los objetivos de la investigación clínica; o

b) garantizar que los intereses de los participantes dignos de protección no se vean afectados negativamente.

Si un participante revoca su consentimiento para participar en el estudio, todos los organismos que hayan almacenado los datos personales, en particular los relativos a la salud, deberán comprobar inmediatamente en qué medida los datos almacenados siguen siendo necesarios para los fines indicados en los apartados a) y b). Los datos que ya no se necesiten deberán ser eliminados inmediatamente.

16. Documentación y administración

16.1 Manual del investigador (MI)

El investigador principal y personal clínico involucrado en la investigación deben ser informados, a través del Manual del investigador, sobre las investigaciones preclínicas o clínicas en relación al Producto Sanitario en Investigación. El manual del investigador se debe actualizar durante el transcurso de la investigación clínica conforme se obtenga información nueva disponible.

16.2 Informe final

Se preparará un informe final de la investigación clínica, aunque la investigación se termine de forma prematura, siguiendo las directrices del Anexo D de la norma ISO 14155:2011.

El informe final se preparará después del análisis estadístico, y deberá ser firmado por el investigador coordinador del estudio, los investigadores principales y el promotor.

16.3 Desviaciones del PIC

El investigador no está autorizado a desviarse del protocolo de la investigación clínica. Debe informar inmediatamente al promotor y al comité de ética correspondiente de cualquier desviación que afecte al bienestar del sujeto.

Siempre se debe obtener la aprobación de ABLE Human Motion S.L. antes de cualquier desviación del PIC. Si la desviación afecta la solidez científica de la investigación clínica, o los derechos del paciente, la seguridad o el bienestar del sujeto y no se trata de una emergencia, debe obtenerse la aprobación previa del Comité de Ética y la Autoridad Nacional Competente correspondiente.

Las desviaciones fuera del control del investigador (como por ejemplo un sujeto que no se presenta a la visita de seguimiento) o a las desviaciones que no afectan la solidez científica del estudio clínico o los derechos, la seguridad o el bienestar del sujeto y no sean una emergencia, deben ser presentadas a medida que sean identificadas por el personal del centro de investigación o de ABLE Human Motion S.L.

16.4 Enmiendas al PIC

Un investigador o un miembro del equipo de la investigación puede proponer cualquier modificación/es que considere apropiada del Plan de Investigación Clínica o del uso del producto sanitario en investigación. ABLE Human Motion S.L. revisará la propuesta y decidirá si la(s) modificación(es) será(n) implementada(s) de acuerdo con los procedimientos escritos para el control de documentos y control de cambios.

ABLE Human Motion S.L. puede decidir revisar el PIC basándose en la nueva información y presentará cualquier enmienda significativa al PIC, incluyendo una justificación para esta enmienda, a las autoridades competentes apropiadas, Comités Éticos y los investigadores. El investigador sólo implementará la enmienda después de la aprobación del Comité Ético, la autoridad competente apropiada y el promotor. Además, los investigadores deberán firmar cualquier enmienda aprobada para su aceptación. Las únicas excepciones son cuando sea necesario eliminar un peligro inmediato para la seguridad de los participantes en el estudio, o

cuando los cambios involucren solamente aspectos administrativos de la investigación clínica (por ejemplo, cambio de número(s) de teléfono).

En el caso de ser necesaria la actualización o modificación del PIC durante la realización de la investigación clínica, ésta se realizará con la presentación de la solicitud de evaluación de enmienda al Comité Ético de Investigación con medicamentos (CEIm) y a la AEMPS según proceda. En ella se registrará la versión y fecha de la nueva versión que reemplazará a la previamente autorizada, según establece la normativa vigente.

17. Suspensión, Interrupción y Finalización de la Investigación Clínica

17.1 Suspensión, interrupción o finalización de un centro de investigación

ABLE Human Motion S.L. se reserva el derecho de suspender al centro de investigación por cualquier de las siguientes razones:

- Fracaso en garantizar el Consentimiento Informado de un paciente inscrito en la investigación.
- Repetidas desviaciones del protocolo.
- Incumplimiento reiterado al completar los CRDs de manera oportuna.
- El investigador solicita la suspensión.

Si el estudio se finaliza o se suspende prematuramente por cualquier motivo, los investigadores informarán sin demora a los participantes del estudio y, cuando así lo exijan los requisitos reglamentarios aplicables, se informará a las autoridades competentes pertinentes. Se informará de forma inmediata a los comités de ética y se les proporcionará una explicación detallada por escrito de la finalización o suspensión.

17.2 Suspensión o interrupción anticipada de la Investigación Clínica

Si la investigación clínica es suspendida o finalizada de forma prematura, el promotor informará inmediatamente al centro de investigación, y si aplica, a las autoridades competentes de la interrupción o suspensión y las razones para ello. Los comités de ética serán informados de forma inmediata y se les proporcionará una explicación detallada de la finalización o suspensión, por el promotor, o por el investigador principal/centro de investigación, tal y como se especifica en los requerimientos regulatorios aplicables.

Algunas de las razones para la suspensión o interrupción anticipada de la investigación son:

- La seguridad de los participantes está comprometida (más de 1 EAG en promedio por paciente).
- Menos de 5 sujetos reclutados hasta 31.06.2021.

17.3 Requisitos para el seguimiento de los pacientes

En caso de suspensión o interrupción anticipada de la investigación clínica, el centro de investigación contactará a los participantes del estudio para realizar el seguimiento.

18. Consideraciones éticas

18.1 Comité de Ética independiente

La versión final del PIC, incluyendo la versión final de la Hoja de Información al Paciente y el Consentimiento Informado (HIP/CI), debe ser aprobada o recibir una opinión favorable por escrito de un Comité de Ética antes del reclutamiento de cualquier sujeto en la investigación clínica.

18.2 Cumplimiento de normativa

Se seguirán de forma estricta las directrices de la Declaración de Helsinki de la Asociación Médica Mundial, las normas de Buena Práctica Clínica y la norma ISO 14155:2011, así como las leyes y reglamentos de los países en que se realice la investigación clínica, incluyendo las leyes de protección de datos y otros requisitos reglamentarios aplicables.

Los investigadores deben cumplir con todos los requisitos establecidos en la ISO 14155:2011 y la legislación nacional. Los investigadores son responsables de la realización de la investigación clínica y del bienestar clínico de los pacientes involucrados. No se permite al investigador(es) desviarse del PIC. La solicitud de cambios debe ser discutida primero con el promotor que, si es necesario, solicitará el permiso al Comité de Ética correspondiente. En el caso de que no sea posible omitir la desviación (por ejemplo, para proteger los derechos, la seguridad y el bienestar de los pacientes), es necesario documentarla y notificarla.

El promotor debe cumplir con los requisitos y responsabilidades establecidos en la norma ISO 14155:2011 y la legislación nacional.

18.3 Hoja de Información al Paciente y el Consentimiento Informado (HIP/CI)

El investigador o su designado autorizado debe obtener consentimiento informado por escrito antes de que se lleve a cabo cualquier actividad relacionada con la investigación clínica.

Se debe entregar a los sujetos la Hoja de Información al Paciente y el Consentimiento Informado (HIP/CI) aprobado por el Comité ético. El investigador o su designado autorizado debe informar al paciente de todos los aspectos de la investigación clínica que son relevantes para tomar la decisión de participar en la investigación clínica. El lenguaje (lenguaje local) utilizado debe ser no técnico y comprensible por el paciente y testigos imparciales, si aplica.

El paciente debe tener el tiempo suficiente para leer y entender el HIP/CI, para pedir detalles sobre la investigación clínica, y para decidir si participar o no en la investigación clínica. Todas las preguntas referentes a la investigación clínica deben ser respondidas a satisfacción de los pacientes. Ni el investigador ni el personal del ensayo deben coaccionar o influir indebidamente en un sujeto para que participe o siga participando en un ensayo. El proceso de consentimiento informado no implica la renuncia de los derechos del paciente.

Si se dispone de nueva información que puede ser relevante para el consentimiento del sujeto, el consentimiento informado escrito y cualquier otra información escrita que se proporcione a los sujetos deberá ser revisada. El sujeto debe ser informado de manera oportuna si hay nueva información disponible que pueda ser relevante para voluntad del sujeto de continuar su participación en el estudio. La comunicación de esta información debe ser documentada.

Si el paciente decide participar en el estudio, el consentimiento informado debe ser firmado y fechado personalmente por el paciente y el investigador o la persona autorizada designada.

Después de que todas las personas hayan firmado y fechado el consentimiento informado, el investigador debe proporcionar al paciente una copia de la hoja de información del paciente y del consentimiento informado firmado y fechado.

Se pondrá a disposición del paciente una tarjeta de contacto.

18.4 Póliza de seguro

Se contratará una póliza de seguro para la validación clínica para cada centro de investigación, de acuerdo con las leyes locales/nacionales. La información básica sobre el seguro se incluirá en la hoja de información al

paciente y se entregará una copia del contrato del seguro a los participantes del estudioen la firma del Consentimiento Informado.

18.5 Confidencialidad

Se informará al paciente de que su inscripción en el estudio clínico será tratada con el mismo grado de confidencialidad que su historial clínico, pero que, de ser necesario, los miembros del Comité Ético y/o el inspector de las autoridades competentes podrán tener acceso a él.

En el cuaderno de recogida de datos electrónico (eCRD), el paciente se identificará únicamente mediante un código de paciente. Todos los datos identificables del paciente serán eliminados de cualquier publicación o comunicación de los resultados del estudio clínico.

El reclutamiento del paciente en la investigación clínica se registrará en su historial clínico.

El investigador completará una lista con los nombres de los sujetos inscritos en el estudio.

19. Divulgación de datos y política de publicación

De acuerdo con la Declaración de Helsinki, después del reclutamiento del primer sujeto se registrará una descripción de la investigación clínica en una base de datos de acceso público.

No se revelará ninguna información confidencial sin obtener el consentimiento previo por escrito del promotor.

Si se publican los datos, se realizará especificando intervalos para, por ejemplo, la edad, el tiempo transcurrido después de la lesión, el nivel de la lesión y los valores de fuerza muscular, manteniendo el riesgo de re-identificación lo más bajo posible.

Los investigadores pueden publicar los resultados de esta investigación; sin embargo, como parte de la información relacionada con el dispositivo de investigación puede ser confidencial, se debe dar primero la oportunidad al promotor de revisar cualquier manuscrito de la publicación antes de su presentación a revistas, reuniones o conferencias.

El promotor designa como colaborador académico al Laboratorio de Ingeniería Biomecánica (BIOMEC), grupo acreditado del Centro de Investigación en Ingeniería Biomédica (CREB) de la Universitat Politècnica de Catalunya (UPC), para liderar el análisis de los resultados y parte de la redacción de los artículos científicos que se publicaran al finalizar la investigación clínica.

20. Referencias

1. Craven, B., Robertson, L., McGillivary, C. & Adachi, J. Detection and Treatment of Sublesional Osteoporosis Among Patients with Chronic Spinal Cord Injury: Proposed Paradigms. *Top Spinal Cord Inj Rehabil* **4**, 1–22 (2009).

2. McIntosh K, R, C., Y, B., U, B. & C., H. The Safety and Feasibility of Exoskeletal-Assisted Walking in Acute Rehabilitation After Spinal Cord Injury. *Arch Phys Med Rehabil* **101**, 113–120 (2020).

3. C, T. *et al.* Initial Outcomes from a Multicenter Study Utilizing the Indego Powered Exoskeleton in Spinal Cord Injury. *Top. Spinal Cord Inj. Rehabil.* **24**, 78–85 (2018).

4. Singh A, Tetreault L, Kalsi-Ryan S, Nouri A, F. M. Global prevalence and incidence of traumatic spinal cord injury. *Clin Epidemiol.* **6**, 309–331 (2014).

5. Pérez, K., Novoa, A. & Santamariña-Rubio E, et al. Working Group for Study of Injuries of Spanish Society of Epidemiology Incidence trends of traumatic spinal cord injury and traumatic brain injury in Spain, 2000–2009. *Accid Anal Prev.* **46**, 37–44 (2012).

6. Martins, F., Freitas, F., Martins, L., Dartigues, J. & Barat, M. Spinal cord injuries – epidemiology in Portugal's central region. *Spinal Cord* **36**, 574–578 (1998).

7. Bach Baunsgaard, C., Vig Nissen, U., Katrin Brust, A. & Al., E. Gait training after spinal cord injury: safety, feasibility and gait function following 8 weeks of training with the exoskeletons from Ekso Bionics. *Spinal Cord* **56**, 106–116 (2018).

8. Harvey, L. Standing and walking with lower limb paralysis. in *Management of Spinal Cord Injuries: A guide for Physiotherapists* 107–136 (2008).

9. Lavis, T. D. & Codamon, L. *23 – Lower Limb Orthoses for Persons With Spinal Cord Injury*. *Atlas of Orthoses and Assistive Devices* (Elsevier Inc., 2018). doi:10.1016/B978-0-323-48323-0.00023-8.

10. Hong, C., Luis, E. B. S. & Chung, S. *Follow-up Study on the Use of Leg Braces Issued to Spinal Cord Injury Patients**. *Parapkgia* vol. 28 (1990).

11. Sykes, L., Edwards, J., Powell, E. S. & Ross, E. R. S. The reciprocating gait orthosis: Long-term usage patterns. *Arch. Phys. Med. Rehabil.* **76**, 779–783 (1995).

12. Franceschini, M., Baratta, S., Zampolini, M., Loria, D. & Lotta, S. Reciprocating gait orthoses: A multicenter study of their use by spinal cord injured patients. *Arch. Phys. Med. Rehabil.* **78**, 582–586 (1997).

13. M., B. *et al.* The efficiency of walking of paraplegic patients using a reciprocating gait orthosis. *Paraplegia* **33**, 409–415 (1995).

14. Kawashima, N., Sone, Y., Nakazawa, K., Akai, M. & Yano, H. Energy expenditure during walking with weight-bearing control (WBC) orthosis in thoracic level of paraplegic patients. *Spinal Cord* **41**, 506–510 (2003).

15. Curt, A., Van Hedel, H. J. A., Klaus, D. & Dietz, V. Recovery from a spinal cord injury: Significance of compensation, neural plasticity, and repair. *J. Neurotrauma* **25**, 677–685 (2008).

16. Arazpour, M., Bani, M. A., Hutchins, S. W. & Jones, R. K. The physiological cost index of walking with mechanical and powered gait orthosis in patients with spinal cord injury. *Spinal Cord* **51**, 356–359 (2013).

17. Guan, X., Kuai, S., Ji, L., Wang, R. & Ji, R. Trunk muscle activity patterns and motion patterns of patients with motor complete spinal cord injury at T8 and T10 walking with different un-powered exoskeletons. *J. Spinal Cord Med.* **40**, 463–470 (2017).

18. Bowker, P., Condie, D., Bader, B., Pratt, D. & Wallace, W. *Biomechanical Basis of Orthotic Management*. (Butterworth-Heinemann Ltd: Oxford, 1993).

19. Kerrigan, D. C., Frates, E. P., Rogan, S. & Riley, P. O. Hip hiking and circumduction: Quantitative definitions. *Am. J. Phys. Med. Rehabil.* **79**, 247–252 (2000).

20. Jeong, W. K., Lee, B. S., Kim, Y. H., Kim, S. K. & Choi, Y. N. Standing and Gait of the Paraplegics by Spinal Cord Injury after Discharge.

21. Mokhtar Arazpour, Monireh Ahmadi Bani, Mohammad Ebrahim Mousavi, M. B. and M. A. M. Orthoses for Spinal Cord Injury Patients. *Intech open* (2016) doi:10.5772/64092.

22. Miller, L., Zimmermann, A. & Herbert, W. Clinical effectiveness and safety of powered exoskeleton-assisted walking in patients with spinal cord injury: systematic review with meta-analysis. *Med. Devices* **9**, 455–466 (2016).

23. Farris, R. J. *et al.* A preliminary assessment of legged mobility provided by a lower limb exoskeleton for persons with paraplegia. *IEEE Trans. Neural Syst. Rehabil. Eng.* **22**, 482–490 (2014).

24. Arazpour, M., Sharifi, G., Mousavi, M. E. & Maleki, M. Role of Gait Training in Recovery of Standing and Walking in Subjects with Spinal Cord Injury. in *Essentials of Spinal Cord Injury Medicine* (InTech, 2018). doi:10.5772/intechopen.71312.

25. Chen, B. *et al.* Recent developments and challenges of lower extremity exoskeletons. *J. Orthop. Transl.* **5**, 26–37 (2016).

26. Sale, P. *et al.* Effects on mobility training and de-adaptations in subjects with Spinal Cord Injury due to a Wearable Robot: a preliminary report. *BMC Neurol.* **16**, 12 (2016).

27. Juszczak, M., Gallo, E. & Bushnik, T. Examining the effects of a powered exoskeleton on quality of life and secondary impairments in people living with spinal cord injury. *Top. Spinal Cord Inj. Rehabil.* (2018) doi:10.1310/sci17-00055.

28. Contreras-Vidal, J., Bhagat, N., Brantley, J., Cruz-Garza, J. & Al., E. Powered exoskeletons for bipedal locomotion after spinal cord injury. *J. Neural Eng.* **13**, 031001 (2016).

29. He, Y., Eguren, D., Luu, T. & Contreras-Vidal, J. Risk management and regulations for lower limb medical exoskeletons: a review. *Med. Devices* **10**, 89–107 (2017).

30. Dijkers, M., Akers, K., Dieffenbach, S. & Galen, S. Systematic Reviews of Clinical Benefits of Exoskeleton Use for Gait and Mobility in Neurologic Disorders: A Tertiary Study. *Arch Phys Med Rehabil* (2019).

31. Yatsuya, K. *et al.* Comparison of energy efficiency between Wearable Power-Assist Locomotor (WPAL) and two types of knee-ankle-foot orthoses with a medial single hip joint (MSH-KAFO). doi:10.1080/10790268.2016.1226701.

32. Hyun Kwon, S. *et al.* Energy Efficiency and Patient Satisfaction of Gait With Knee-Ankle-Foot Orthosis and Robot (ReWalk)-Assisted Gait in Patients With Spinal Cord Injury. *Ann. Rehabil. Med. Orig. Artic. Ann Rehabil Med* **44**, 131–141 (2020).

33. De Sanidad, M. & Consumo, Y. *GUÍA DESCRIPTIVA DE ORTOPRÓTESIS TOMO II ORTESIS DE MIEMBRO SUPERIOR Y MIEMBRO INFERIOR GUÍA DESCRIPTIVA DE ORTOPRÓTESIS CONSEJO INTERTERRITORIAL DEL SISTEMA NACIONAL DE SALUD ,! 7 I I 4 H 6-h a g b i d !*

34. Font-Llagunes, J. M., Lugrís, U., Clos, D., Alonso, F. J. & Cuadrado, J. Design, Control, and Pilot Study of a Lightweight and Modular Robotic Exoskeleton for Walking Assistance After Spinal Cord Injury. *J. Mech. Robot.* **12**, (2020).

35. Harvey, L. Cardiovascular fitness training. in *Management of Spinal Cord Injuries: A guide for Physiotherapists* 227–241 (2008).

36. Kolakowsky-Hayner, SA, Crew, J., Moran, S. & Shah, A. Safety and Feasibility of using the EksoTM Bionic Exoskeleton to Aid Ambulation after Spinal Cord Injury. *J Spine* **S4**, 003 (2013).

37. Gagnon, D., Escalona, M., Vermette, M. & Al., E. Locomotor training using an overground robotic exoskeleton in long-term manual wheelchair users with a chronic spinal cord injury living in the community: Lessons learned from a feasibility study in terms of recruitment, attendance, learnability, performa. *J. Neuroeng. Rehabil.* **15**, 12 (2018).

38. Wu, C. *et al.* The effects of gait training using powered lower limb exoskeleton robot on individuals with complete spinal cord injury. *J Neuroeng Rehabil* **15**, 14 (2018).

39. Sale, P. *et al.* Training for mobility with exoskeleton robot in spinal cord injury patients: a pilot study. *Eur J Phys Rehabil Med.* **54**, 745–751 (2018).

40. Sale, P. *et al.* Effects on mobility training and de-adaptations in subjects with Spinal Cord Injury due to a Wearable Robot: a preliminary report. *BMC Neurol* **16**, 12 (2016).

41. Yang, A., Asselin, P., Knezevic, S., Kornfeld, S. & Spungen, A. Assessment of In-Hospital Walking Velocity and Level of Assistance in a Powered Exoskeleton in Persons with Spinal Cord Injury. *Top. Spinal Cord Inj. Rehabil.* **21**, 100–109 (2015).

42. Spungen, A., Asselin, P., Fineberg, D., Kornfeld, S. & Harel, N. Exoskeletal-assisted walking for persons with motor-complete paraplegia. *Pap. Present. NATO Sci. Technol. Organ.* (2013).

43. Delgado, A. *et al.* Safety and feasibility of exoskeleton-assisted walking during acute/sub-acute SCI in an inpatient rehabilitation facility: A single-group preliminary study. *J Spinal Cord Med* **11**, 1–10 (2019).

44. Aach, M., Cruciger, O., Sczesny-Kaiser, M. & Al., E. Voluntary driven exoskeleton as a new tool for rehabilitation in chronic spinal cord injury: a pilot study. *Spine J* **14**, 2847–53 (2014).

45. Kozlowski, A., Bryce, T. & Dijkers, M. Time and Effort Required by Persons with Spinal Cord Injury to Learn to Use a Powered Exoskeleton for Assisted Walking. *Top Spinal Cord Inj Rehabil* **21**, 110–121 (2015).

46. Del-Ama, A., Gil-Agudo, Á., Pons, J. & Moreno, J. Hybrid gait training with an overground robot for people with incomplete spinal cord injury: a pilot study. *Front. Hum. Neurosci* **8**, 298 (2014).

47. Khan, A. S., Livingstone, D. C., Hurd, C. L. & Al., E. Retraining walking over ground in a powered exoskeleton after spinal cord injury: a prospective cohort study to examine functional gains and neuroplasticity. *J NeuroEngineering Rehabil* **16**, 145 (2019).

48. Hartigan, C. *et al.* Mobility Outcomes Following Five Training Sessions with a Powered Exoskeleton. *Top Spinal Cord Inj Rehabil* **21**, 93–9 (2015).

49. Platz, T., Gillner, A., Borgwaldt, N., Kroll, S. & Roschka, S. Device-Training for Individuals with Thoracic and Lumbar Spinal Cord Injury Using a Powered Exoskeleton for Technically Assisted Mobility: Achievements and User Satisfaction. *Biomed Res Int.* **8459018**, (2016).

50. Xiang, X., Ding, M., Zong, H. & Al., E. The safety and feasibility of a new rehabilitation robotic exoskeleton for assisting individuals with lower extremity motor complete lesions following spinal cord injury (SCI): an observational study. *Spinal Cord.* (2020).

51. van Herpen, F. *et al.* Case Report: Description of two fractures during the use of a powered exoskeleton. *Spinal Cord Ser. Cases* **5**, 99 (2019).

52. Kendall, M. G. *A Million Random Digits with 100,000 Normal Deviates. Economica* vol. 22 (RAND, 1955).

53. Martel, G., Noreau, L. & Jobin, J. Physiological responses to maximal exercise on arm cranking and wheelchair ergometer with paraplegics. *Paraplegia* **29**, 447–456 (1991).

54. Paré, G., Noreau, L. & Simard, C. Prediction of maximal aerobic power from a submaximal exercise test performed by paraplegics on a wheelchair ergometer. *Paraplegia* **31**, 584–592 (1993).

55. Al-Rahamneh, H. Q. & Eston, R. G. The validity of predicting peak oxygen uptake from a perceptually guided graded exercise test during arm exercise in paraplegic individuals. *Spinal Cord* **49**, 430–434 (2011).

56. Al-Rahamneh, H. Q. & Eston, R. G. Prediction of peak oxygen consumption from the ratings of perceived exertion during a graded exercise test and ramp exercise test in able-bodied participants and paraplegic persons. *Arch. Phys. Med. Rehabil.* **92**, 277–283 (2011).

57. Biering-Sørensen, F., DeVivo, M., Charlifue, S. & Al., E. International Spinal Cord Injury Core Data Set (version 2.0) — including standardization of reporting. *Spinal Cord* **55**, 759–764 (2017).

58. Betz, R., Biering-Sørensen, F., Burns, S. & Al, E. The 2019 revision of the International Standards for Neurological Classification of Spinal Cord Injury (ISNCSCI)—What's new? *Spinal Cord* **57**, 815–817 (2019).

59. Craven, B. & A., M. Modified Ashworth scale reliability for measurement of lower extremity spasticity among patients with SCI. *Spinal Cord* **48**, 207–213 (2010).

60. Poncumhak, P., Saengsuwan, J., Kamruecha, W. & Amatachaya, S. Reliability and validity of three functional tests in ambulatory patients with spinal cord injury. *Spinal Cord* **51**, 214–7 (2012).

61. Alexander, M., Anderson, K., Biering-Sorensen, F. & Al., E. Outcome measures in spinal cord injury: recent assessments and recommendations for future directions. *Spinal Cord* **47**, 582–591 (2009).

62. Scivoletto, G., Tamburella, F., Laurenza, L. & Al., E. Validity and reliability of the 10-m walk test and the 6-min walk test in spinal cord injury patients. *Spinal Cord* **49**, 736–740 (2011).

63. BORG, G. Psychophysical scaling with applications in physical work and the perception of exertion. *Scand J Work Env. Heal.* **16**, 55–8 (1990).

64. Bergstrom, A. & Samuelsson, K. Evaluation of manual wheelchairs by individuals with spinal cord injuries. *Disabil Rehabil Assist Technol* **1**, 175–182 (2006).

65. Demers, L., Weiss-Lambrou, R. & Ska, B. The Quebec User Evaluation of Satisfaction with Assistive Technology (QUEST 2.0): An overview and recent progress. *Technol. Disabil.* **14**, 101–105 (2002).

66. Jeffrey, J. & Day, H. Psychosocial Impact of Assistive Devices Scale (PIADS). *Technol. Disabil.* **14**, 107–111 (2002).

67. Fundarò, C. *et al.* Motor and psychosocial impact of robot-assisted gait training in a real-world rehabilitation setting: A pilot study. *PLoS One* **13**, e0191894 (2018).

68. *Prevention and Treatment of Pressure Ulcers/Injuries: Quick Reference Guide*. (European Pressure Ulcer Advisory Panel, National Pressure Injury Advisory Panel and Pan Pacific Pressure Injury Alliance., 2019).

69. Kressler, J. & Domingo, A. Cardiometabolic Challenges Provided by Variable Assisted Exoskeletal Versus Overground Walking in Chronic Motor-incomplete Paraplegia: A Case Series. *J. Neurol. Phys. Ther.* **43**, 128–135 (2019).

70. Evans, N., Hartigan, C., Kandilakis, C., Pharo, E. & Clesson, I. Acute Cardiorespiratory and Metabolic Responses During Exoskeleton-Assisted Walking Overground Among Persons with Chronic Spinal Cord Injury. *Top. Spinal Cord Inj. Rehabil.* **21**, 122–132 (2015).

71. Asselin, P. *et al.* Heart rate and oxygen demand of powered exoskeleton-assisted walking in persons with paraplegia. **52**, 147–158 (2015).
